# Genetic heterogeneity and prognostic impact of recurrent *ANK2* and *TP53* mutations in mantle cell lymphoma: a multi-centre cohort study

**DOI:** 10.1038/s41598-020-70310-9

**Published:** 2020-08-07

**Authors:** Seri Jeong, Yu Jin Park, Woobin Yun, Seung-Tae Lee, Jong Rak Choi, Cheolwon Suh, Jae-Cheol Jo, Hee Jeong Cha, Jee-Yeong Jeong, HeeKyung Chang, Yoon Jin Cha, Hyerim Kim, Min-Jeong Park, Wonkeun Song, Eun-Hae Cho, Eun-Goo Jeong, Junnam Lee, Yongmin Park, Yong Seok Lee, Da Jung Kim, Ho Sup Lee

**Affiliations:** 1 Department of Laboratory Medicine, Kangnam Sacred Heart Hospital, Hallym University College of Medicine, Seoul, 07441 South Korea; 2 Department of Laboratory Medicine, Brain Korea 21 PLUS Project for Medical Science, Yonsei University College of Medicine, Seoul, 03722 South Korea; 3 Department of Oncology, Asan Medical Center, University of Ulsan College of Medicine, Ulsan, 05505 South Korea; 4 Department of Haematology and Oncology, Ulsan University Hospital, University of Ulsan College of Medicine, Ulsan, 44033 South Korea; 5 Department of Pathology, Ulsan University Hospital, University of Ulsan College of Medicine, Ulsan, 44033 South Korea; 6 Department of Biochemistry, Kosin University College of Medicine, Busan, 49267 South Korea; 7 Department of Pathology, Kosin University Gospel Hospital, University of Kosin College of Medicine, Busan, 49267 South Korea; 8 Department of Pathology, Gangnam Severance Hospital, Yonsei University College of Medicine, Seoul, 06273 South Korea; 9 Department of Laboratory Medicine, Pusan National University Hospital, Busan, 49241 Korea; 10 Department of Genomics, Green Cross Genome, Yongin, 16924 South Korea; 11 Department of Bioinformatics, Samsung SDS, Seoul, 05510 South Korea; 12 Division of Haematology-Oncology, Department of Internal Medicine, Kosin University College of Medicine, 262 Gamcheon-ro, Seo-gu, 49267 Busan, South Korea

**Keywords:** Molecular medicine, Cancer genetics, Haematological cancer

## Abstract

The molecular features of mantle cell lymphoma (MCL), including its increased incidence, and complex therapies have not been investigated in detail, particularly in East Asian populations. In this study, we performed targeted panel sequencing (TPS) and whole-exome sequencing (WES) to investigate the genetic alterations in Korean MCL patients. We obtained a total of 53 samples from MCL patients from five Korean university hospitals between 2009 and 2016. We identified the recurrently mutated genes such as *SYNE1*, *ATM*, *KMT2D*, *CARD11*, *ANK2*, *KMT2C*, and *TP53*, which included some known drivers of MCL. The mutational profiles of our cohort indicated genetic heterogeneity. The significantly enriched pathways were mainly involved in gene expression, cell cycle, and programmed cell death. Multivariate analysis revealed that *ANK2* mutations impacted the unfavourable overall survival (hazard ratio [HR] 3.126; *P* = 0.032). Furthermore, *TP53* mutations were related to worse progression-free survival (HR 7.813; *P* = 0.043). Among the recurrently mutated genes with more than 15.0% frequency, discrepancies were found in only 5 genes from 4 patients, suggesting comparability of the TPS to WES in practical laboratory settings. We provide the unbiased genetic landscape that might contribute to MCL pathogenesis and recurrent genes conferring unfavourable outcomes.

## Introduction

Mantle cell lymphoma (MCL) is a mature B-cell non-Hodgkin’s lymphoma (NHL) accounting for 5–7% of all NHLs^1^. Although MCL is uncommon (0.64/100,000 person-years), there has been a 130.9% rise in its incidence in recent years^2^. Higher incidence rates have been seen in Caucasians (153.0%), Asians (96.4%), and the elderly (429.2%). Patients with MCL treated with standard therapies have a limited duration of remission, and also show frequent relapses, resulting in a short median overall survival (OS) of 4–5 years^3^.

Classical MCL expresses the hallmark t(11:14)(q13;q32) chromosomal translocation^4^. However, the pathogenesis of MCL is complex and the alterations in the genes involved in DNA damage response (ataxia telangiectasia mutated [*ATM*], checkpoint kinase 2 [*CHK2*], and *TP53*) and cell survival signalling pathways (Bruton’s tyrosine kinase [BTK], mammalian target of rapamycin [mTOR], nuclear factor-κB [NF-κB], tumour necrosis factor [TNF], and NOTCH) also play crucial roles^5^. Moreover, MCL patients exhibit diverse responses to therapies and clinical courses^6,7^, which has not been completely incorporated by current risk stratification tools. Therefore, it is essential to investigate the genetic landscape of this heterogeneous disease to gain insight into the molecular pathogenesis and to predict the prognosis of MCL patients.

Targeted panel sequencing (TPS) and whole-exome sequencing (WES) have been used as influential tools for discovering the molecular mechanisms of haematologic malignancies^8-12^. Differences in genomic signatures according to ethnicity have been found in previous MCL studies^8-16^. However, there has been no report of the genetic landscape in Korean or East-Asian MCL patients, which are rarely reported as study populations. Therefore, we aimed to investigate the genetic alterations in MCL patients in Korea using both TPS and WES. Prognostic factors including clinical parameters and gene mutations were also analysed. Furthermore, we conducted a comparative analysis between TPS and WES to determine the ideal prediction tool for MCL patients.

## Results

### Clinical characteristics

The clinical data of the 50 MCL patients enrolled in this study are summarized in Table 1. The median age was 62.5 years. This cohort predominantly consisted of men (70.0%) and patients with advanced stages (stage IV, 68.0%). The typical t(11;14)(q13;q32) translocation was found in 6 patients (12.0%). One patient had +8,t(8;14)(q24;q32) chromosomal aberration. These chromosomal abnormalities were identified by classical cytogenetics, and not by fluorescence in situ hybridization (FISH). Eleven patients (22.0%) belonged to the high risk group according to mantle cell lymphoma international prognostic index (MIPI). Most patients (82.0%) received rituximab combined chemotherapy such as R-CHOP and R-hyper-CVAD (Supplementary Table S1). The complete and partial remission rates were 44.0% and 22.0%, respectively. The estimated OS rates were 62.1% for 3-year, and 55.2% for 5-year.

**Table 1:** Clinical data and sample information of patients with mantle cell lymphoma.

Characteristics	Value*	Missing*
Age (years)	62.5 (56.9–70.0)	0 (0.0)
Sex, male	35 (70.0)	0 (0.0)
β2-MG, elevated (≥ 254.5 nmol/L)	24 (48.0)	0 (0.0)
LDH, elevated (≥ 4.67 ukat/L)	22 (44.0)	0 (0.0)
Ki-67, high (≥ 30%)	21 (42.0)	17 (34.0)
Cytogenetic aberration	6 (12.0)	0 (0.0)
Stage IV	34 (68.0)	2 (4.0)
MIPI		12 (24.0)
Low	5 (10.0)	
Intermediate	22 (44.0)	
High	11 (22.0)	
IPI		8 (16.0)
Low	7 (14.0)	
Low-intermediate	13 (26.0)	
High-intermediate	18 (36.0)	
High	4 (8.0)	
R-Chemotherapy^a^	41 (82.0)	0 (0.0)
ASCT	9 (18.0)	0 (0.0)
Radiotherapy	5 (10.0)	0 (0.0)
Efficacy		10 (20.0)
Complete remission	22 (44.0)	
Partial remission	11 (22.0)	
Stable disease	2 (4.0)	
Progressive disease	5 (10.0)	
Sample status, relapse	19 (38.0)	18 (36.0)
OS (months)	33.0 (12.8–56.0)	
PFS (months)	19.5 (9.9–37.7)	32 (64.0)

ASCT, autologous stem cell transplantation; CR, complete remission; IPI, international prognostic index; LDH, lactate dehydrogenase; MG, microglobulin; MIPI, mantle cell lymphoma international prognostic index; OS, overall survival; PD, progressive disease; PFS, progression free survival; PR, partial remission; SD, stable disease. *Data are expressed as the median (1st and 3rd quartiles) or number (percentage).

^a^Rituximab combined chemotherapy.

### MCL mutational landscape

**Figure 1:**
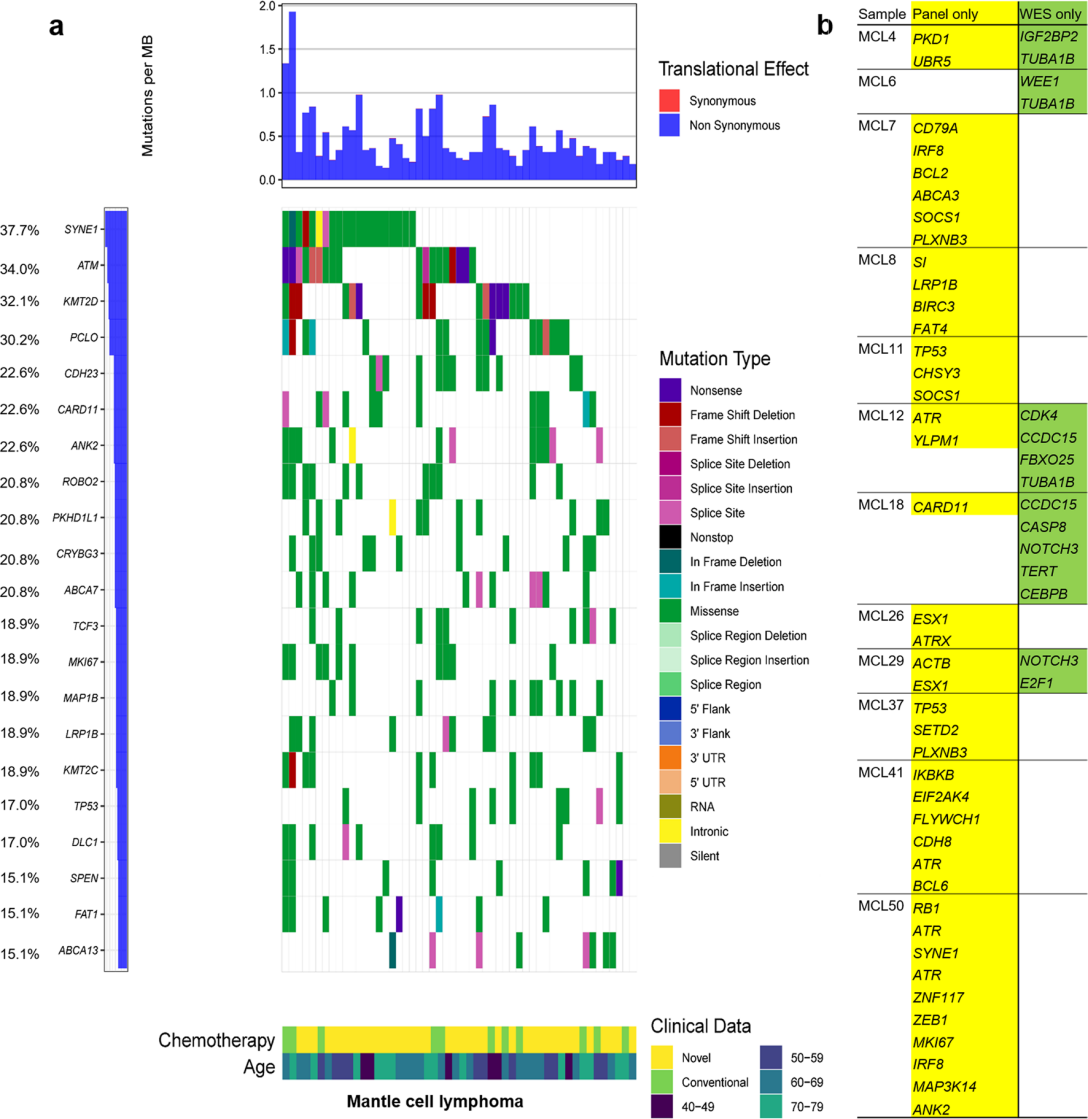
The mutational spectrum of 53 patients with mantle cell lymphoma. (**a**) Mutational spectrum with frequencies and clinical data. (**b**) Genes identified in the lymphoma panel only (left) or by whole-exome sequencing (WES) only (right).

A lymphoma panel was performed on 53 formalin-fixed paraffin-embedded (FFPE) samples derived from MCL patients. Among them, WES was conducted on 16 FFPE samples (4 saliva samples for germline control). The mutational profiles of 53 MCL patients are presented in Fig. 1a, showing genetic heterogeneity. *SYNE1*, *ATM*, and *KMT2D* were the most three common genes having mutations in our MCL cohort (69.8%). We analysed these genes using cBioPortal software (https://www.cbioportal.org/index.do)17. The *SYNE1* and *KMT2D* mutations were frequently found in cutaneous squamous cell carcinoma (cSCC), and have also been reported in previous studies on MCL^8,18^. The frequencies of *SYNE1* (37.7%), and *KMT2D* mutations (32.1%) in our cohort were higher than those in other MCL cohort studies (6.9% for *SYNE1*^8^, and 12% to 23% for *KMT2D*^8–10,12,18^). *ATM* mutations showed a frequency of 34.0% in our cohort, and previous MCL studies showed that this gene is consistently mutated with a high frequency (15% to 56%^8-16^). *ANK2* mutations, detected in cSCC and NHL, were present at a higher frequency (22.6%) in our study than in previous MCL cohort studies^12^. *ANK2* encodes ankyrin-2, which plays a crucial role in cell development^12^. *TP53* mutations have been detected in most studies on genomic alterations in MCL^11,12^, and this mutation was also reported in our cohort (17.0%). Samples obtained from patients who relapsed showed a higher frequency of *TP53* mutation (26.3%) than primary samples (15.4%). However, the difference was not significant (*P* = 0.463). Among the frequently mutated genes associated with MCL, only *SYNE1* (*P* = 0.020 for Chi-square test, and hazard ratio [HR] 0.167; *P* = 0.025 for binary logistic regression) was significantly related to primary samples, when compared to relapsed samples. Regarding to mean variant allele frequency, the values of relapsed samples were significantly higher than those of primary samples for *SYNE1* (32.4% vs. 60.2%; *P* = 0.013), *ANK2* (35.0% vs. 55.7%; *P* = 0.042), and *TP53* (30.1% vs. 72.4%; *P* = 0.024) mutations. Ten out of 53 patients harboured *KMT2C* and *MAP1B* mutations, which were frequently found in patients with small cell lung cancer^19^ and NHL ^18,20-23^. The frequencies of recurrently mutated genes including *ATM*, *BIRC3*, *CCND1*, *KMT2D*, *NOTCH1*, *TP53*, *TRAF2*, *UBR5*, and *WHSC1* in other MCL studies compared to those in our study are presented in Supplementary Table S2.

Genes that were detected only in one of the lymphoma panel or WES are presented in Fig. 1b. Among the recurrently mutated genes with more than 15.0% frequency, discrepancies were found in only 5 genes from 4 patients, which showed the comparability of the two methods.

### Prognostic risk factors

Univariate and multivariate analyses were performed to investigate the risk factor for the prognosis of MCL patients (Table 2). OS and progression-free survival (PFS) were assigned as independent factors for prognosis, while clinical and genetic mutations were considered as co-variates. The factor that significantly correlated with worse OS was old age (HR 1.086; *P* = 0.009). High tumour proliferation index (Ki-67 ≥ 30%) and high risk group of MIPI, known as factors of poor prognosis, showed 5.372 and 2.341 of HR for OS, respectively. However, their *P*-values could not reach the predefined 0.05. The recurrently mutated genes with a frequency of > 15.0% were also included in our prognostic analysis. *ANK2* (HR 3.403; *P* = 0.011) and *KMT2C* (HR 4.305; *P* = 0.002) mutations were related to worse OS based on our univariate analysis. In multivariate analysis, *ANK2* was determined as an independent predictor (HR 3.126; *P* = 0.032). Age, and *KMT2C* showing *P*-values less than 0.05 in univariate analysis were co-variates for the multivariate analysis for *ANK2* gene. The patients with *ANK2* mutations showed significantly shorter median survival time (26.3 months) than those without (124.9 months) (*P* = 0.010) according to survival analysis.

**Table 2:** Univariate and multivariate analyses for overall survival and progression-free survival in mantle cell lymphoma patients.

Factors	Overall survival				Progression-free survival			
	Univariate		Multivariate		Univariate		Multivariate	
	HR (95% CI)	*P* value	HR (95% CI)	*P* value	HR (95% CI)	*P* value	HR (95% CI)	*P* value
Age	1.086 (1.019–1.157)	0.009	1.075 (1.002–1.154)	0.044	1.066 (0.975–1.165)	0.153		
Sex, male	0.399 (0.147–1.085)	0.063			0.694 (0.165–2.924)	0.617		
β2-MG, elevated (≥ 3.0 µg/mL)	2.411 (0.855–6.801)	0.087			2.367 (0.563–9.956)	0.226		
LDH, elevated (≥ 280 U/L)	1.490 (0.546–4.069)	0.434			1.655 (0.348–7.873)	0.523		
Ki-67, high (≥ 30%)	5.372 (0.666–43.311)	0.077			NA			
Cytogenetic aberration	0.609 (0.077–4.487)	0.605			4.500 (0.466–43.482)	0.154		
Stage IV	0.722 (0.273–1.910)	0.510			2.680 (0.532–13.512)	0.217		
MIPI, high versus low and intermediate	2.341 (0.744–7.369)	0.135			3.311 (0.383–28.611)	0.249		
IPI, high versus low	1.203 (0.420–3.448)	0.731			0.818 (0.197–3.404)	0.782		
Rituximab combined chemotherapy	0.423 (0.154–1.167)	0.088			0.844 (0.215–3.305)	0.807		
ASCT	0.219 (0.029–1.673)	0.109			1.006 (0.121–8.385)	0.996		
Radiotherapy	0.634 (0.139–2.896)	0.553			0.967 (0.198–4.717)	0.967		
Genetic mutations*								
*SYNE1*	0.754 (0.261–2.181)	0.601			0.537 (0.065–4.406)	0.556		
*ATM*	0.905 (0.328–2.497)	0.847			0.733 (0.148–3.626)	0.702		
*KMT2D*	1.255 (0.458–3.434)	0.658			0.602 (0.125–2.910)	0.524		
*PCLO*	2.380 (0.910–6.224)	0.069			1.873 (0.499–7.028)	0.345		
***ANK2***^a^	3.403 (1.255–9.229)	0.011	3.126 (1.105–8.845)	0.032	1.562 (0.387–6.304)	0.528		
*CARD11*	2.554 (0.952–6.855)	0.054			0.863 (0.177–4.198)	0.855		
*CDH23*	0.779 (0.220–2.765)	0.699			0.240 (0.028–2.053)	0.164		
*ABCA7*	0.520 (0.180–1.504)	0.219			0.800 (0.191–3.349)	0.759		
*CRYBG3*	1.348 (0.468–3.881)	0.579			1.514 (0.370–6.190)	0.561		
*PKHD1L1*	0.323 (0.073–1.431)	0.118			0.643 (0.133–3.113)	0.580		
*ROBO2*	2.135 (0.767–5.945)	0.137			1.898 (0.313–11.504)	0.479		
***KMT2C***	4.305 (1.592–11.639)	0.002	2.751 (0.969–7.813)	0.057	6.116 (1.009–37.064)	0.025	3.482 (0.311–39.008)	0.311
*LRP1B*	1.317 (0.419–4.145)	0.637			0.614 (0.118–3.196)	0.559		
***MAP1B***	1.727 (0.550–5.418)	0.343			15.392 (1.333–177.654)	0.004	12.403 (0.732–210.025)	0.081
*MKI67*	1.492 (0.480–4.641)	0.486			0.630 (0.077–5.174)	0.665		
*TCF3*	1.099 (0.352–3.429)	0.871			5.604 (0.682–9.942)	0.147		
***TP53***^a^	1.121 (0.315–3.989)	0.859			9.300 (1.490–58.049)	0.004	7.813 (1.065–57.293)	0.043
*DLC1*	2.081 (0.705–6.137)	0.175			2.580 (0.429–15.531)	0.283		

ASCT, autologous stem cell transplantation; CI, confidence interval; HR, hazard ratio; IPI, international prognostic index; LDH, lactate dehydrogenase; MG, macroglobulin; MIPI, mantle cell lymphoma international prognostic index; NA, not applicable due to the paucity of positive or negative data.

*Genes with a frequency of more than 17.0% and *P* < 0.05 in univariate analyses for overall survival or progression-free survival are indicated in bold.

^a^*P* < 0.05 in multivariate analyses for overall survival or progression-free survival.

**Figure 2:**
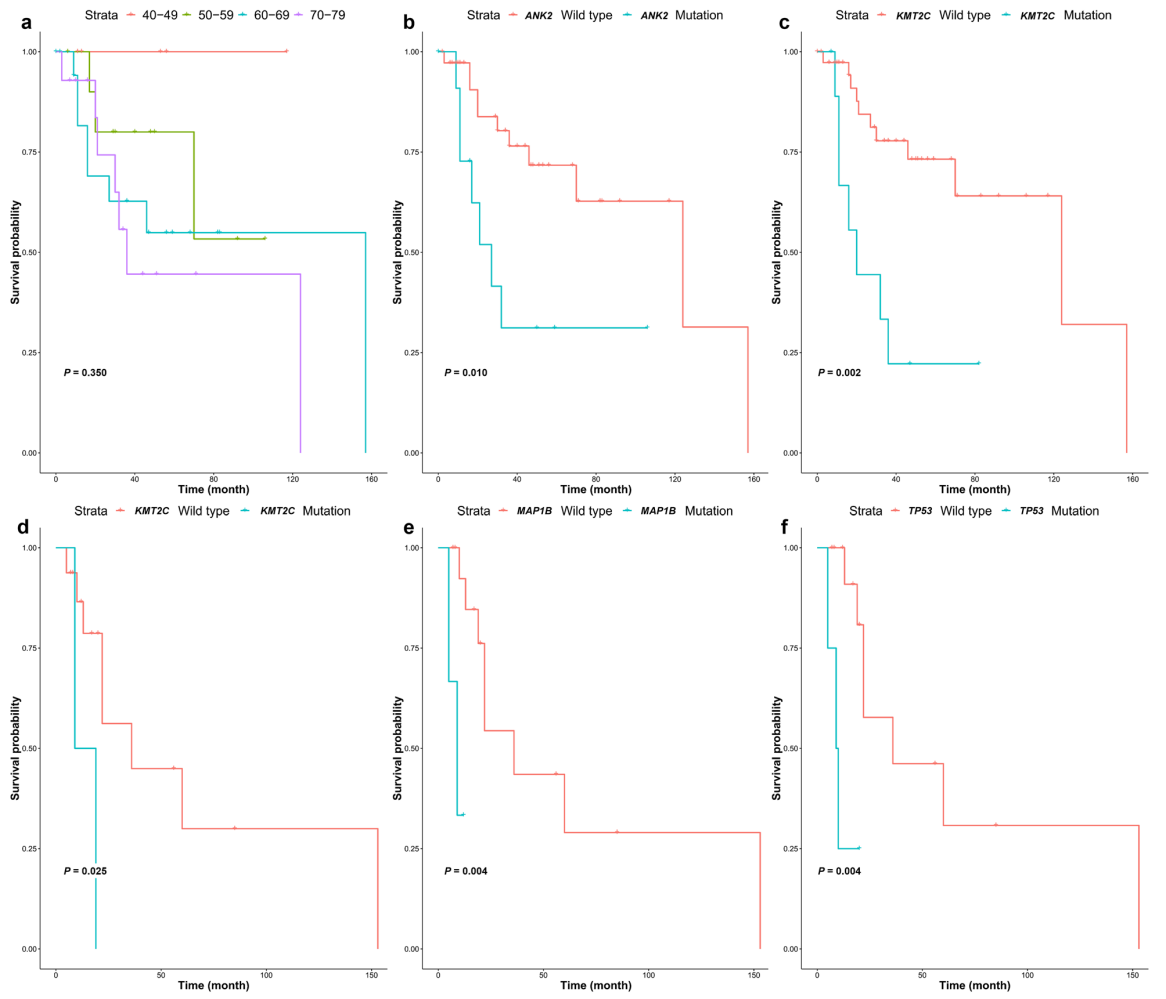
Kaplan–Meier curves for overall survival and progression-free survival of patients with mantle cell lymphoma. Kaplan–Meier curves of (**a**) age classes for overall survival, (**b**) *ANK2* mutation for overall survival, (**c**) *KMT2C* mutation for overall survival, (**d**) *KMT2C* mutation for progression-free survival, (**e**) *MAP1B* mutation for progression-free survival, and (**f**) *TP53* mutation for progression-free survival. The presented *P*-values were calculated using the log-rank test.

Regarding to PFS, *TP53* (HR 9.300; *P* = 0.004), *KMT2C* (HR 6.116; *P* = 0.025), and *MAP1B* (HR 15.392; *P* = 0.004) were associated based on univariate analysis. Among them, *TP53* was a significant predictor of PFS according to multivariate analysis (HR 7.813; *P* = 0.043). Patients with *TP53* mutations had significantly shorter median PFS time (8.0 months) than those without (47.5 months) (*P* = 0.004). The survival curves calculated based on patient age and the presence of mutated genes using the Kaplan–Meier method are illustrated in Fig. 2a-f.

### Pathways affected by mutations

**Figure 3:**
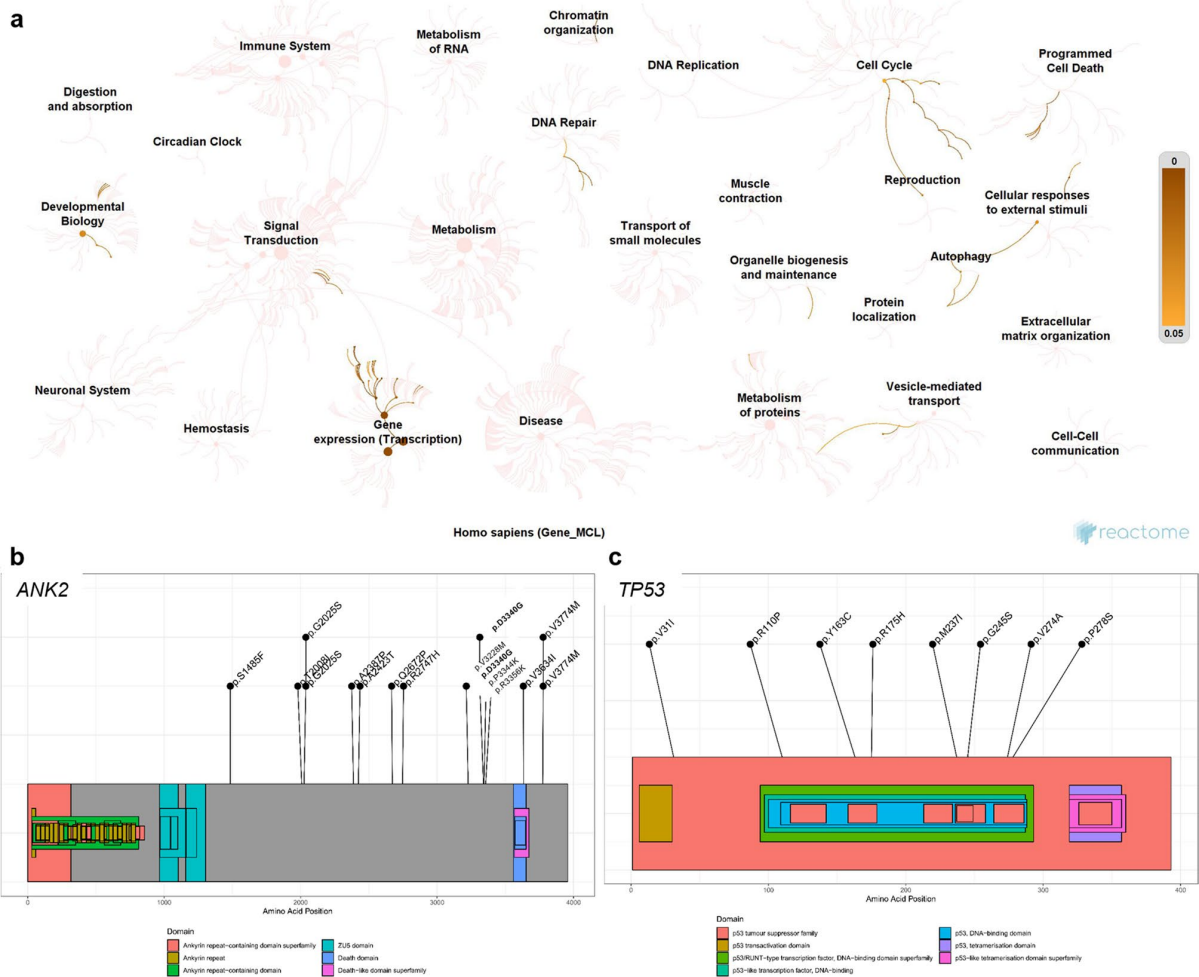
Pathway analysis and amino acid changes resulting from the determined *ANK2* and *TP53* mutations. (**a**) Pathway analysis of mutational genes with a frequency of more than 15.0% in mantle cell lymphoma samples. (**b**) The location of amino acid changes resulting from *ANK2* and (**c**) *TP53* mutations.

We conducted functional enrichment analysis of the mutated genes in our MCL cohort (Fig. 3a). The specific names of the 25 most significant pathways, which mainly consisted of pathways related to gene expression, cell cycle, and programmed cell death, are presented in Supplementary Table S3. *TP53*, *ATM*, and *KMT2D* are mainly involved in gene expression and cell cycle pathways such as regulation of TP53 expression and auto-degradation of the E3 ubiquitin ligase COP1. Meanwhile, *ANK2* was found to be involved in pathways related to developmental biology and vesicle-mediated transport. The focused locations of amino acid changes on *ANK2* and *TP53* mutations in this cohort are illustrated in Fig. 3b and c, respectively. The changes of p.D3340G and p.3774 M were presented recurrently in MCL patients with *ANK2* mutations.

## Discussion

To the best of our knowledge, this is the first study reporting the TPS and WES for comprehensive genomic investigation of MCL patients in Korea. We have shown the heterogeneous spectrum of the genetic mutations of this form of lymphoma. We have also discovered several molecular mechanisms contributing to its pathogenesis. Furthermore, we analysed the impact of recurrent *ANK2* and *TP53* mutations for clinical prognosis including OS and PFS. We also investigated the comparison between TPS and WES to suggest more appropriate laboratory settings.

Among the commonly mutated genes in our study, *SYNE1*, *ATM*, *KMT2D*, *CARD11*, *ANK2*, *ROBO2*, *CRYBG3*, *KMT2C*, *TP53*, and *DLC1* were previously identified in MCL studies^1,8,11,12,18^. The mutational profiles shown in our cohort including the detected genes, variations, and frequencies showed substantial genetic heterogeneity, consistent with previous studies. Mutations in *CDH23*, *ABCA7*, *LRP1B*, *MAP1B*, *MKI67*, *TCF3*, and *ABCA13*, rarely reported in previous MCL studies, presented more than 15.0% in our study. Ethnic variations, specimen status, the applied reagents and equipment, sequence depth, and the coverage of the target genes may have influenced these differences.

In this study, we most commonly identified *SYNE1* mutations (37.7%), which were detected at a rate of 6.9% in a previous MCL cohort study^8^. The *SYNE1* was listed as one of the recurrently mutated genes in MCL and diffuse large B-cell lymphoma literatures^1^. Furthermore, a study for frequent mutation of histone modifying genes in NHL also reported *SYNE1* mutation^24^. This gene, located on 6q25.1–25.2, encodes nesprin-1, which is involved in communication between the nuclear lamina and the cytoskeleton^25^. Nesprin-1 is critical for nuclear positioning and anchorage. This protein has been correlated in dilated cardiomyopathy^26^. In cancers, *SYNE1* showed medium and high expression in lymphoma patients and likely drive transformation of lymphoma^27^. Regarding to the relapsed status, the frequency of *SYNE1* mutations was lower than that of primary samples. However, the mean allele frequency of *SYNE1* mutations was significantly increased in relapsed samples compared to initial samples. In a previous study, *SYNE1* mutations were reported as relapse-associated mutations in paediatric acute lymphoblastic leukaemia^28^. A minor clone with a *SYNE1* mutation was identified at the first relapse, and it became dominant at the second relapse. Moreover, Shah et al.^29^ suggested a plausible role of CD44v-SYNE1-miRNA34a axis as biomarkers to diagnose oral cancer at an early stage and predict the early onset of metastasis. Although the underlying mechanism of *SYNE1* gene is unraveled and the relation to tumourigenesis is controversial, *SYNE1* mutation might be used at least as a biomarker for relapsed status, considering the mean allele frequencies of our cohort. Further investigating these results on a larger sample set is necessary.

The second most frequently detected mutation found in our study was in *ATM* (34.0%), which has been a previously identified MCL driver. *ATM* mutations have been found in 41.4% of Western patients and 37.5% of Chinese patients^8,11^. The *ATM* mutations included nonsense and frameshift mutations, similar to those observed in previous MCL studies. We found that various mutated forms of *ATM* were involved in gene expression and cell cycle pathways. In particular, this gene encodes a tumour suppressor involved in DNA damage response^30^. Additionally, *ATM* mutations are related to the inactivation of the ARF-TP53 tumour suppressor pathway^31^. These forms have also been reported in different subtypes of lymphoid malignancies^32,33^.

*KMT2D* showed a mutation rate of 32.1% in this study compared to 12–23% in previously reported MCL studies^18^. The epigenetic modifier, *KMT2D*, has been identified as an early MCL driver^34^. We also frequently detected *KMT2D* nonsense and frameshift mutations in our cohort, supporting its contribution to lymphomagenesis. Somatic mutations leading to inactivation of the KMT2D methyltransferase perturb germinal center B cell development and promote lymphomagenesis by remodeling the epigenetic landscape of the cancer precursor cells^35^.

*ANK2* and *TP53* were significantly related to OS (HR 3.126) and PFS (HR 7.813), respectively. A higher mutation frequency (22.6%) was observed in *ANK2* encoding ankyrin-2^12,36^. This protein is crucial for the localization and membrane stabilization of ion transporters and ion channels, especially in cardiomyocytes. Therefore, ankyrin mutations are generally associated with cardiac arrhythmia and sudden cardiac death^37^. In terms of malignancies, the silencing of *ANK2* expression reduced the growth and invasion of pancreatic cancer cells, indicating its potential as a target for therapy^38^. Further, *ANK2* expression levels had a significant correlation with the clinical outcome in gastrointestinal cancer^39^. Regarding to MCL, 5.6% (3/56) of *ANK2* mutations were identified from the lymph node samples of Caucasian male patients in a previous study^12^. The described results analyzed by PolyPhen-2 included damaging (1.0), and possibly damaging (0.7) predictions. Similar to this study, our cohort also revealed damaging and possibly damaging predictions based on the scores analyzed by SIFT (0.001 to 0.034) and PolyPhen-2 (0.627 to 0.999). *ANK2* encodes Ankyrin-2, which belongs to a family of cytoskeletal proteins mediating linkage of integral membrane proteins with the spectrin-actin based skeleton. Ankyrin-2 is involved in pathways associated with a variety of biological activities such as cell motility, activation, proliferation, contact, and the maintenance of specialized membrane domains^36^. Ankyrin repeat domain, a highly conserved membrane-binding domain shared by ankyrin encoding genes, is seemed to be directly associated with the binding of ankyrins to various types of proteins. In particular, binding to CD44, which is a transmembrane glycoprotein mediating lots of important activities of tumour cells, has been reported. This interaction was responsible for a more severe malignant phenotype in cancer cells^40^. Regarding to cancers, ankyrins influence on signal transduction, cell adhesion, membrane transport, cell growth, migration and metastasis of cancer cells^38,40^. In terms of relapse, some breast cancer samples in the poor prognosis group revealed significantly higher ANK2 expression, indicating that ANK2 may be related to personalized relapse mechanism^41^. Cao et al.^42^ demonstrated that signaling pathways including ANK2 were modulated by miR-647 and mediated the proliferation and metastasis of gastric cancer cells. In our study, the mean allele frequencies of *ANK2* mutations were significantly increased in relapsed samples, suggesting its oncogenic function in lymphomagenesis. However, further investigations are required with large sample sizes to validate the impact of *ANK2* mutations in MCL patients.

With respect to PFS, we found that *TP53* was found to be an efficient predictor based on our multivariate analysis. Several studies into MCL have demonstrated the association of *TP5*3 mutations with poor clinical outcomes^11,43^. *TP53* alterations were previously associated with a poor prognosis in MCL patients treated with standard treatment modalities^44,45^. A recent study suggested that allogeneic hematopoietic cell transplantation may be a beneficial treatment option for patients with *TP53* mutations^46^. For the younger MCL patients receiving cytarabine-containing chemotherapy and autologous stem cell transplantation, Ferrero et al.^9^ developed the MIPI-genetic index (MIPI-g), which is a prognostic model integrating MIPI-c prognostic index with genetic data (*TP53*, and *KMT2D* mutations). Despite being a different study population, we applied the MIPI-g to our cohort and found that the high risk patients were significantly related to worse PFS (HR 20.601; *P* = 0.025) (Supplementary Fig. S1). Based on these findings, we propose that *TP53* should be routinely assessed as a molecular marker to determine the prognosis, as well as to guide treatment decisions. In respect of relapse, a study on younger MCL patients showing 11% of mutation frequency and 26.9% of mean allele frequency for *TP53* mutations demonstrated the independent prognostic impact of *TP53* mutations on time to relapse^47^.

To the best of our knowledge, there has been no previous report investigating TPS and WES concurrently using the same lymphoma samples. We compared TPS and WES for the first time in this study to improve the practical laboratory settings for the MCL patients. Among the recurrently mutated genes with more than 15.0% frequency, we found discrepancies in only 5 genes (*TP53*, *CARD11*, *SYNE1*, *MKI67*, and *ANK2*) from 4 patients, which shows the comparability of these two settings. However, utilized platforms, the read depth and coverage of the target (365.9X for TPS, and 144.0X for WES) may influence on these results. A previous comparison study, which applied TPS and WES to patients with inherited retinal dystrophy, demonstrated that TPS including 291 genes could be used as a first-tier test^48^.

This study had several limitations. The study population was relatively small, reflecting the uncommon incidence of MCL in Korea. Moreover, the samples with unknown state for disease course lessened the available analysis, and statistical power. Further study with a much larger number of patients might enable subgroup analysis and demonstrate stronger statistical relationship between genetic mutations and prognosis. Additional confirmation using Sanger sequencing could not be conducted for mutations with low read depth due to lack of remaining DNA samples. Performing this analysis would serve to rule out false positives, and should therefore be performed in future studies to confirm the current results. In addition, fresh samples rather than FFPE specimens, which were available in this multicenter study, might provide better quality of sequencing data.

In conclusion, we performed TPS and WES for the comprehensive genomic investigation of MCL patients using samples from five university hospitals in Korea, a racially homogeneous country of East Asia for the first time. This study has revealed the heterogeneous spectrum in the genetic alterations of MCL. We not only identified several mutated genes such as *SYNE1*, *ATM*, and *KMT2D*, which contribute to pathogenesis, but also showed that recurrent *ANK2* and *TP53* mutations had negative impacts on the OS and PFS, respectively. In particular, our study suggests that TPS may be comparable to WES in practical laboratory settings. In the future, the sequencing of these identified genes will benefit MCL patients by improving the prognosis and the choice of therapeutic interventions.

## Methods

### Patients and sample preparation

A total of 53 samples were collected from MCL patients from five university hospitals in Korea between March 2009 and October 2016. The FFPE specimens that were stored at the Kosin University Gospel Hospital (n = 26), Asan Medical center (n = 11), Ulsan University Hospital (n = 8), Gangnam Severance Hospital (n = 6), and Pusan National University Hospital (n = 2) were obtained. The MCL samples were confirmed after a diagnosis was made based on the criteria established by the World Health Organization classification with mantle zone B cell phenotypes^49^. As the chromosomal aberrations were identified by classical cytogenetics, it might have been affected by laboratory settings such as sample state, resolution of the image, and interpretation, additional pathological confirmation by pathologists from the Kosin University Gospel Hospital was conducted. Further, pathologists also confirmed that all samples were at least positive for cyclin D1. The tested samples were lymph nodes containing more than 80% tumour cells, as confirmed by the pathologists.

Records of medical visits, demographic information, and clinical data were collected and reviewed for all patients diagnosed with MCL. The sample selection procedure used for evaluating the genetic and prognostic parameters is shown in Supplementary Fig. S2. This study was approved by the independent Institutional Review Board of Kosin University Gospel Hospital (KUGH 2017-02-011) and conducted in accordance with the Declaration of Helsinki. We obtained informed consents from all the MCL patients and personal information was protected and kept anonymous.

### TPS and WES

A total of 588 genes known to be related to lymphoma were included in our gene panel; the genes are listed in Supplementary Table S4. The targeted lymphoma panel consisted of frequently mutated genes in the previously reported 26 studies of lymphoma including particularly MCL^1,8,10-12,18,21,50-68^. The mutated genes in lymphoma samples deposited in databases such as Catalogue of Somatic Mutations in Cancer (COSMIC), ClinVar, and Human Gene Mutation Database (HGMD) were also selected for broader range of targets of TPS. Briefly, the sequencing data were analysed as follows. Genomic DNA was extracted from 55 FFPE tissue samples using a deparaffinization solution and the QIAamp Blood DNA mini kit (Qiagen, Hilden, Germany). We constructed genomic DNA libraries and captured the custom lymphoma panel using the Library Preparation Kit (Celemics, Seoul, Korea). The pooled libraries were sequenced using a NextSeq sequencer (Illumina, San Diego, CA, USA) and the NextSeq Reagent Kit v2 (500 cycles). The average read depth of 53 samples was 365.9X. The read depths and coverages per samples are presented in Supplementary Table S5.

The Agilent SureSelect Human All Exon platform (Agilent Technologies Inc., Santa Clara, CA, USA) was used for WES to capture the target DNA samples and generate standard exome libraries. The entire exome regions for 12 FFPE tumour tissues and 4 saliva samples from 12 MCL patients were sequenced using the HiSeq 2500 platform with a paired-end read protocol (Illumina). The saliva samples were prepared using AccuSaliva collection kits (AccuGene Inc., Incheon, Korea) for normal control. All tumour and normal samples were sequenced with an average read depth of 144.0X. The read depths and coverages per samples are shown in Supplementary Table S6.

The filtered reads were aligned to the reference assembly of hg19 reference sequence using BWA software (version 0.7.5). Indel realignment and base quality score recalibration were performed using MuTect2 in GATK software (version 3.7) to identify somatic single-nucleotide variants (SNVs) based on the Catalogue of Somatic Mutations in Cancer (COSMIC) database^69^. Short indels were identified using VarScan (version 2.3). The mutations (SNVs and indels) found in each sample were annotated using ANNOVAR software. The quality of the mutations in the sequenced BAM files were manually reviewed using Integrated Genomics Viewer (Broad Institute., Cambridge, MA, USA) to filter out false positives. The mean numbers of mutations detected by TPS and WES were 9184.9, and 1023.5, respectively. Among the mutations identified by TPS and WES, the numbers of filtered and reported mutations were 698 for TPS, and 291 for WES. The threshold for total read depth of the reported mutations was 10X. All single nucleotide polymorphisms with a frequency of > 1% in the Korean Variant Archive, 1000 Genomes Project, esp6500 database, and EXAC database were removed. We found no significant differences between the mutations that were adjusted with the variations of normal control and those without. The variants that were determined to be pathogenic or likely pathogenic based on the American College of Medical Genetics and Genomics, COSMIC, and the Association for Molecular Pathology classification^70^ were considered causative mutations for MCL. Subsequently, genotype–phenotype correlations were discussed by clinical pathologists and haemato-oncologists. The significant mutational profiles of 53 MCL patients including variant allele frequency, read depth, and SIFT and PolyPhen-2 predictions are presented in Supplementary Table S7. The presented variant of allele frequency was more than 5%.

### Pathway and survival statistical analysis

Functional enrichment analysis of the recurrently mutated genes was performed using the Reactome tool^71^. Descriptive statistics were used for the characteristics and sample information of the MCL patients. Multivariate Cox proportional-hazards regression models were used to examine the factors correlated with OS or PFS. OS was determined from the date of diagnosis to the date of death from any causes (event), or the last follow up (censoring). PFS was calculated from the date of treatment to the date of disease progression (event), death from any causes (event), or the last follow up (censoring)^72^ for the MCL patients including primary and relapsed patients in our cohort as post relapse survival might lead to biased estimates based on the report of García-Albéniz et al.^73^. The Kaplan–Meier method with log-rank test was used to estimate the survival curves. Statistical analyses were performed using RStudio statistical software (version 3.6.0; R Foundation for Statistical Computing, Vienna, Austria) and SPSS (version 24.0; IBM, Armonk, NY, USA). Values of *P* < 0.05 were considered statistically significant.

## Supplementary information

Supplementary Information.

## Data Availability

All data generated or analyzed during this study are included in this published article (Tables, Figures, and Supplementary information) and available from the corresponding author on reasonable request. The data discussed in this study have been deposited in NCBI’s Sequence Read Archive and are accessible through accession number SRR10572983.

## References

[1] Wu C (2016). Genetic heterogeneity in primary and relapsed mantle cell lymphomas: Impact of recurrent CARD11 mutations. Oncotarget.

[2] Aschebrook-Kilfoy B, Caces DB, Ollberding NJ, Smith SM, Chiu BC (2013). An upward trend in the age-specific incidence patterns for mantle cell lymphoma in the USA. Leuk Lymphoma.

[3] Kang BW (2014). Clinical features and treatment outcomes in patients with mantle cell lymphoma in Korea: study by the Consortium for Improving Survival of Lymphoma. Blood Res..

[4] Perez-Galan P, Dreyling M, Wiestner A (2011). Mantle cell lymphoma: biology, pathogenesis, and the molecular basis of treatment in the genomic era. Blood.

[5] Jares P, Campo E (2008). Advances in the understanding of mantle cell lymphoma. Br. J. Haematol..

[6] Choi YJ (2019). Efficacy of the novel CDK7 inhibitor QS1189 in mantle cell lymphoma. Sci. Rep..

[7] Ratnasingam S (2019). Cytarabine-based induction immunochemotherapy in the front-line treatment of older patients with mantle cell lymphoma. Sci. Rep..

[8] Bea S (2013). Landscape of somatic mutations and clonal evolution in mantle cell lymphoma. Proc. Natl. Acad. Sci. USA.

[9] Ferrero S (2019). KMT2D mutations and TP53 disruptions are poor prognostic biomarkers in mantle cell lymphoma receiving high-dose therapy: a FIL study. Haematologica.

[10] Rossi D, Ciardullo C, Gaidano G (2013). Genetic aberrations of signaling pathways in lymphomagenesis: revelations from next generation sequencing studies. Semin. Cancer Biol..

[11] Yang P (2018). Genomic landscape and prognostic analysis of mantle cell lymphoma. Cancer Gene Ther..

[12] Zhang J (2014). The genomic landscape of mantle cell lymphoma is related to the epigenetically determined chromatin state of normal B cells. Blood.

[13] Greiner TC (2006). Mutation and genomic deletion status of ataxia telangiectasia mutated (ATM) and p53 confer specific gene expression profiles in mantle cell lymphoma. Proc. Natl. Acad. Sci. USA.

[14] Kridel R (2012). Whole transcriptome sequencing reveals recurrent NOTCH1 mutations in mantle cell lymphoma. Blood.

[15] Meissner B (2013). The E3 ubiquitin ligase UBR5 is recurrently mutated in mantle cell lymphoma. Blood.

[16] Rahal R (2014). Pharmacological and genomic profiling identifies NF-kappaB-targeted treatment strategies for mantle cell lymphoma. Nat Med.

[17] Gao J (2013). Integrative analysis of complex cancer genomics and clinical profiles using the cBioPortal. Sci. Signal.

[18] Rosenquist R, Bea S, Du MQ, Nadel B, Pan-Hammarstrom Q (2017). Genetic landscape and deregulated pathways in B-cell lymphoid malignancies. J. Intern. Med..

[19] Gardner EE (2017). Chemosensitive relapse in small cell lung cancer proceeds through an EZH2-SLFN11 Axis. Cancer Cell.

[20] Chapuy B (2018). Molecular subtypes of diffuse large B cell lymphoma are associated with distinct pathogenic mechanisms and outcomes. Nat Med.

[21] Morin RD, Gascoyne RD (2013). Newly identified mechanisms in B-cell non-Hodgkin lymphomas uncovered by next-generation sequencing. Semin. Hematol..

[22] Reddy A (2017). Genetic and functional drivers of diffuse large B cell lymphoma. Cell.

[23] Roschewski M (2015). Circulating tumour DNA and CT monitoring in patients with untreated diffuse large B-cell lymphoma: a correlative biomarker study. Lancet Oncol..

[24] Morin RD (2011). Frequent mutation of histone-modifying genes in non-Hodgkin lymphoma. Nature.

[25] Taranum S (2012). Cytoskeletal interactions at the nuclear envelope mediated by nesprins. Int. J. Cell Biol..

[26] Zhou C (2017). Novel nesprin-1 mutations associated with dilated cardiomyopathy cause nuclear envelope disruption and defects in myogenesis. Hum. Mol. Genet..

[27] Bouska A (2017). Combined copy number and mutation analysis identifies oncogenic pathways associated with transformation of follicular lymphoma. Leukemia.

[28] Lindqvist CM (2016). Deep targeted sequencing in pediatric acute lymphoblastic leukemia unveils distinct mutational patterns between genetic subtypes and novel relapse-associated genes. Oncotarget.

[29] Shah K, Patel S, Modi B, Shah F, Rawal R (2018). Uncovering the potential of CD44v/SYNE1/miR34a axis in salivary fluids of oral cancer patients. J. Oral Pathol. Med..

[30] Klener P (2019). Advances in molecular biology and targeted therapy of mantle cell lymphoma. Int. J. Mol. Sci..

[31] Gronbaek K (2002). ATM mutations are associated with inactivation of the ARF-TP53 tumor suppressor pathway in diffuse large B-cell lymphoma. Blood.

[32] de Miranda NF (2014). Exome sequencing reveals novel mutation targets in diffuse large B-cell lymphomas derived from Chinese patients. Blood.

[33] Rossi D (2012). Disruption of BIRC3 associates with fludarabine chemorefractoriness in TP53 wild-type chronic lymphocytic leukemia. Blood.

[34] Jiang Y (2014). Deep sequencing reveals clonal evolution patterns and mutation events associated with relapse in B-cell lymphomas. Genome Biol..

[35] Zhang J (2015). Disruption of KMT2D perturbs germinal center B cell development and promotes lymphomagenesis. Nat. Med..

[36] Cunha SR, Mohler PJ (2009). Ankyrin protein networks in membrane formation and stabilization. J. Cell Mol. Med..

[37] Mohler PJ (2003). Ankyrin-B mutation causes type 4 long-QT cardiac arrhythmia and sudden cardiac death. Nature.

[38] Chen Y, Lohr M, Jesnowski R (2010). Inhibition of ankyrin-B expression reduces growth and invasion of human pancreatic ductal adenocarcinoma. Pancreatology.

[39] Hu W, Yang Y, Ge W, Zheng S (2019). Deciphering molecular properties of hypermutated gastrointestinal cancer. J. Cell Mol. Med..

[40] Zhu D, Bourguignon LY (2000). Interaction between CD44 and the repeat domain of ankyrin promotes hyaluronic acid-mediated ovarian tumor cell migration. J. Cell. Physiol..

[41] Chen X (2017). Identification of breast cancer recurrence risk factors based on functional pathways in tumor and normal tissues. Oncotarget.

[42] Cao W (2017). Role of miR-647 in human gastric cancer suppression. Oncol. Rep..

[43] Nordstrom L (2014). SOX11 and TP53 add prognostic information to MIPI in a homogenously treated cohort of mantle cell lymphoma–a Nordic Lymphoma Group study. Br. J. Haematol..

[44] Delfau-Larue MH (2015). High-dose cytarabine does not overcome the adverse prognostic value of CDKN2A and TP53 deletions in mantle cell lymphoma. Blood.

[45] Eskelund CW (2018). Lenalidomide plus bendamustine-rituximab does not overcome the adverse impact of TP53 mutations in mantle cell lymphoma. Haematologica.

[46] Lin RJ (2019). Allogeneic haematopoietic cell transplantation impacts on outcomes of mantle cell lymphoma with TP53 alterations. Br. J. Haematol..

[47] Eskelund CW (2017). TP53 mutations identify younger mantle cell lymphoma patients who do not benefit from intensive chemoimmunotherapy. Blood.

[48] Wang L (2018). Application of whole exome and targeted panel sequencing in the clinical molecular diagnosis of 319 Chinese Families with inherited retinal dystrophy and comparison study. Genes (Basel).

[49] Gao J, Peterson L, Nelson B, Goolsby C, Chen YH (2009). Immunophenotypic variations in mantle cell lymphoma. Am. J. Clin. Pathol..

[50] Ahmed M, Zhang L, Nomie K, Lam L, Wang M (2016). Gene mutations and actionable genetic lesions in mantle cell lymphoma. Oncotarget.

[51] Braggio E, Egan JB, Fonseca R, Stewart AK (2013). Lessons from next-generation sequencing analysis in hematological malignancies. Blood Cancer J..

[52] Blenk S (2008). Explorative data analysis of MCL reveals gene expression networks implicated in survival and prognosis supported by explorative CGH analysis. BMC Cancer.

[53] Chiron D (2014). Cell-cycle reprogramming for PI3K inhibition overrides a relapse-specific C481S BTK mutation revealed by longitudinal functional genomics in mantle cell lymphoma. Cancer Discov..

[54] Inamdar AA (2016). Mantle cell lymphoma in the era of precision medicine-diagnosis, biomarkers and therapeutic agents. Oncotarget.

[55] Reis-Sobreiro M (2013). Lipid raft-mediated Akt signaling as a therapeutic target in mantle cell lymphoma. Blood Cancer J..

[56] Sanchez-Tillo E (2014). The EMT activator ZEB1 promotes tumor growth and determines differential response to chemotherapy in mantle cell lymphoma. Cell Death Differ..

[57] Zhong W (2017). Increased expression of IRF8 in tumor cells inhibits the generation of Th17 cells and predicts unfavorable survival of diffuse large B cell lymphoma patients. Oncotarget.

[58] Desai S (2010). PRDM1 is required for mantle cell lymphoma response to bortezomib. Mol. Cancer Res..

[59] Ghobrial IM (2005). Proteomic analysis of mantle-cell lymphoma by protein microarray. Blood.

[60] Kurtova AV, Tamayo AT, Ford RJ, Burger JA (2009). Mantle cell lymphoma cells express high levels of CXCR4, CXCR5, and VLA-4 (CD49d): importance for interactions with the stromal microenvironment and specific targeting. Blood.

[61] Liu H (2008). Transvection mediated by the translocated cyclin D1 locus in mantle cell lymphoma. J. Exp. Med..

[62] Martin-Moreno AM (2015). CSF1R protein expression in reactive lymphoid tissues and lymphoma: its relevance in classical hodgkin lymphoma. PLoS ONE.

[63] Morimoto K (2016). LRRK1 is critical in the regulation of B-cell responses and CARMA1-dependent NF-kappaB activation. Sci. Rep..

[64] Pathak P, Li Y, Gray BA, May WS, Markham MJ (2017). Synchronous occurrence of chronic myeloid leukemia and mantle cell lymphoma. Case Rep. Hematol..

[65] Rinaldi A (2006). Genomic and expression profiling identifies the B-cell associated tyrosine kinase Syk as a possible therapeutic target in mantle cell lymphoma. Br. J. Haematol..

[66] Sayan AE (2014). Tumour-promoting role of EMT-inducing transcription factor ZEB1 in mantle cell lymphoma. Cell Death Differ..

[67] Smith SM (2012). Targeting mTOR in mantle cell lymphoma: current and future directions. Best Pract. Res. Clin. Haematol..

[68] Ye H (2000). BCL10 expression in normal and neoplastic lymphoid tissue. Nuclear localization in MALT lymphoma. Am. J. Pathol..

[69] Cibulskis K (2013). Sensitive detection of somatic point mutations in impure and heterogeneous cancer samples. Nat. Biotechnol..

[70] https://www.nature.com/articles/nbt.2514#supplementary-information (2013).

[71] Li MM (2017). Standards and guidelines for the interpretation and reporting of sequence variants in cancer: a Joint Consensus Recommendation of the Association for Molecular Pathology, American Society of Clinical Oncology, and College of American Pathologists. J Mol Diagn.

[72] Fabregat A (2017). Reactome pathway analysis: a high-performance in-memory approach. BMC Bioinform..

[73] Cheson BD (2007). Revised response criteria for malignant lymphoma. J. Clin. Oncol..

[74] Garcia-Albeniz X, Maurel J, Hernan MA (2015). Why post-progression survival and post-relapse survival are not appropriate measures of efficacy in cancer randomized clinical trials. Int. J. Cancer.

