# Supplementary material for: Genetic heterogeneity and prognostic impact of recurrent ANK2 and TP53 mutations in mantle cell lymphoma: a multi-centre cohort study
Source: Sci Rep. 2020 Aug 7;10:13359. doi: 10.1038/s41598-020-70310-9 (PMC7414214; doi:10.1038/s41598-020-70310-9)
Supplement: Supplementary file 1 — Supplementary Information. [file 41598_2020_70310_MOESM1_ESM.pdf]

Supplementary Information

Genetic heterogeneity and prognostic impact of recurrent *ANK2* and *TP53* mutations in mantle cell lymphoma: A multi-centre cohort study

Seri Jeong[1,13], Yu Jin Park[2,13], Woobin Yun[2], Seung-Tae Lee[2], Jong Rak Choi[2], Cheolwon Suh[3], Jae-Cheol Jo[4], Hee Jeong Cha[5], Jee-Yeong Jeong[6], HeeKyung Chang[7], Yoon Jin Cha[8], Hyerim Kim[9], Min-Jeong Park[1], Wonkeun Song[1], Eun-Hae Cho[10], Eun-Goo Jeong[10], Junnam Lee[10], Yongmin Park[11], Yong Seok Lee[11], Da Jung Kim[12] & Ho Sup Lee[12*]

[1]Department of Laboratory Medicine, Kangnam Sacred Heart Hospital, Hallym University College of Medicine, Seoul, 07441, South Korea. [2]Department of Laboratory Medicine, Yonsei University College of Medicine, Seoul, 03722, South Korea. [3]Department of Oncology, Asan Medical Center, University of Ulsan College of Medicine, Ulsan, 05505, South Korea. [4]Department of Haematology and Oncology, Ulsan University Hospital, University of Ulsan College of Medicine, Ulsan, 44033, South Korea. [5]Department of Pathology, Ulsan University Hospital, University of Ulsan College of Medicine, Ulsan, 44033, South Korea. [6]Department of Biochemistry, Kosin University College of Medicine, Busan, 49267, South Korea. [7]Department of Pathology, Kosin University Gospel Hospital, University of Kosin College of Medicine, Busan, 49267, South Korea. [8]Department of Pathology, Gangnam Severance Hospital, Yonsei University College of Medicine, Seoul, 06273, South Korea. [9]Department of Laboratory Medicine, Pusan National University

Hospital, Busan, 49241, Korea. [10]Department of Genomics, Green Cross Genome, Yongin, 16924, South Korea. [11]Department of Bioinformatics, Samsung SDS, Seoul, 05510, South Korea. [12]Division of Haematology-Oncology, Department of Internal Medicine, Kosin University College of Medicine, Busan, 49267, South Korea. [13]These authors contributed equally: Seri Jeong and Yu Jin Park.

* Corresponding author: Ho Sup Lee

Division of Haematology-Oncology, Department of Internal Medicine, Kosin University College of Medicine, 262 Gamcheon-ro, Seo-gu, 49267, Busan, South Korea.

Tel: (+82) 51-990-6363, Fax: (+82) 51-990-5820

E-mail: hs52silver@gmail.com

Supplemental table and figure legends

Supplementary Figure S1. Kaplan-Meier curves classified by MIPI-genetic index for overall survival and progression-free survival of patients with mantle cell lymphoma. Kaplan-Meier curves of (a) classes for overall survival, (b) classes for progression-free survival. **Supplementary Figure S2.** Flow chart of sample selection.

Supplementary Table S1. Clinical data and sample information of mantle cell lymphoma patients in our cohort.

Supplementary Table S2. Comparison with other mantle cell lymphoma studies for recurrently mutated genes.

Supplementary Table S3. Most significant 25 pathways sorted by *P*-values.

Supplementary Table S4. The 588 gene list of targeted lymphoma panel set used for next-generation sequencing experiments.

Supplementary Table S5. Read depth and coverage of targeted lymphoma panel sequencing.

Supplementary Table S6. Read depth and coverage of whole-exome sequencing.

Supplementary Table S7. Genetic variants identified in 53 patients with mantle cell lymphoma.

Supplementary Figure S1.

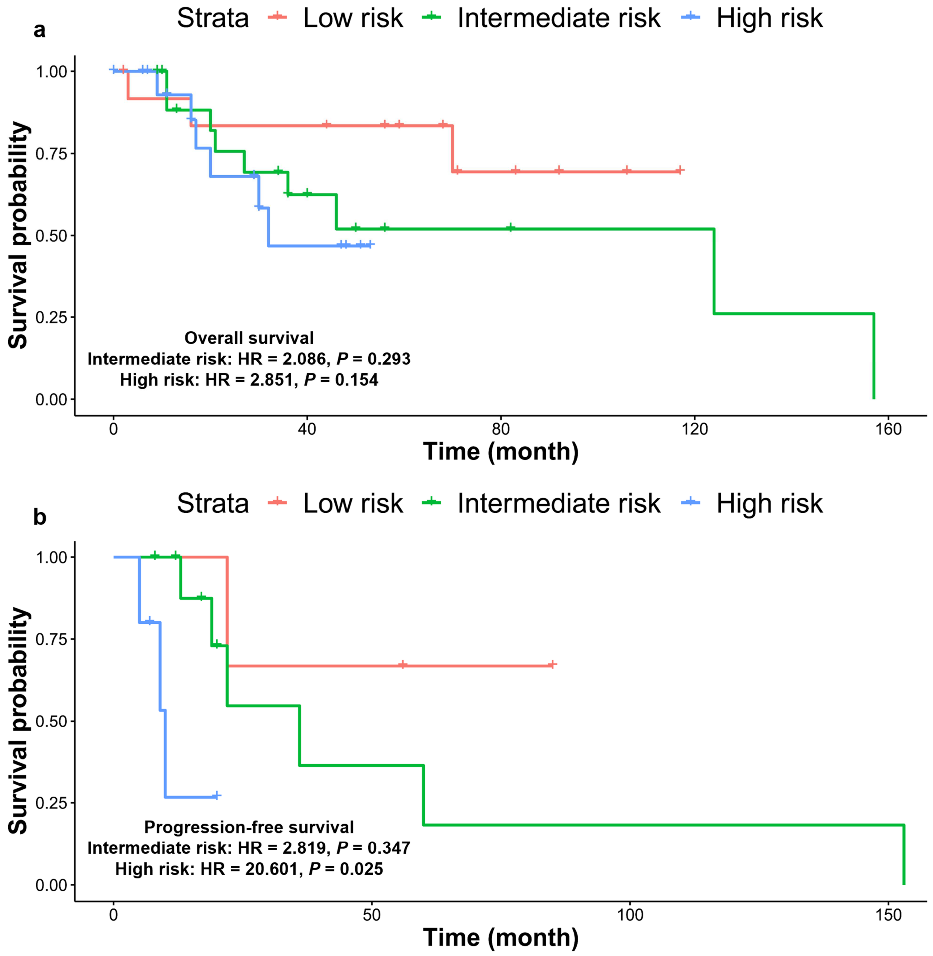

Supplementary Figure S2.

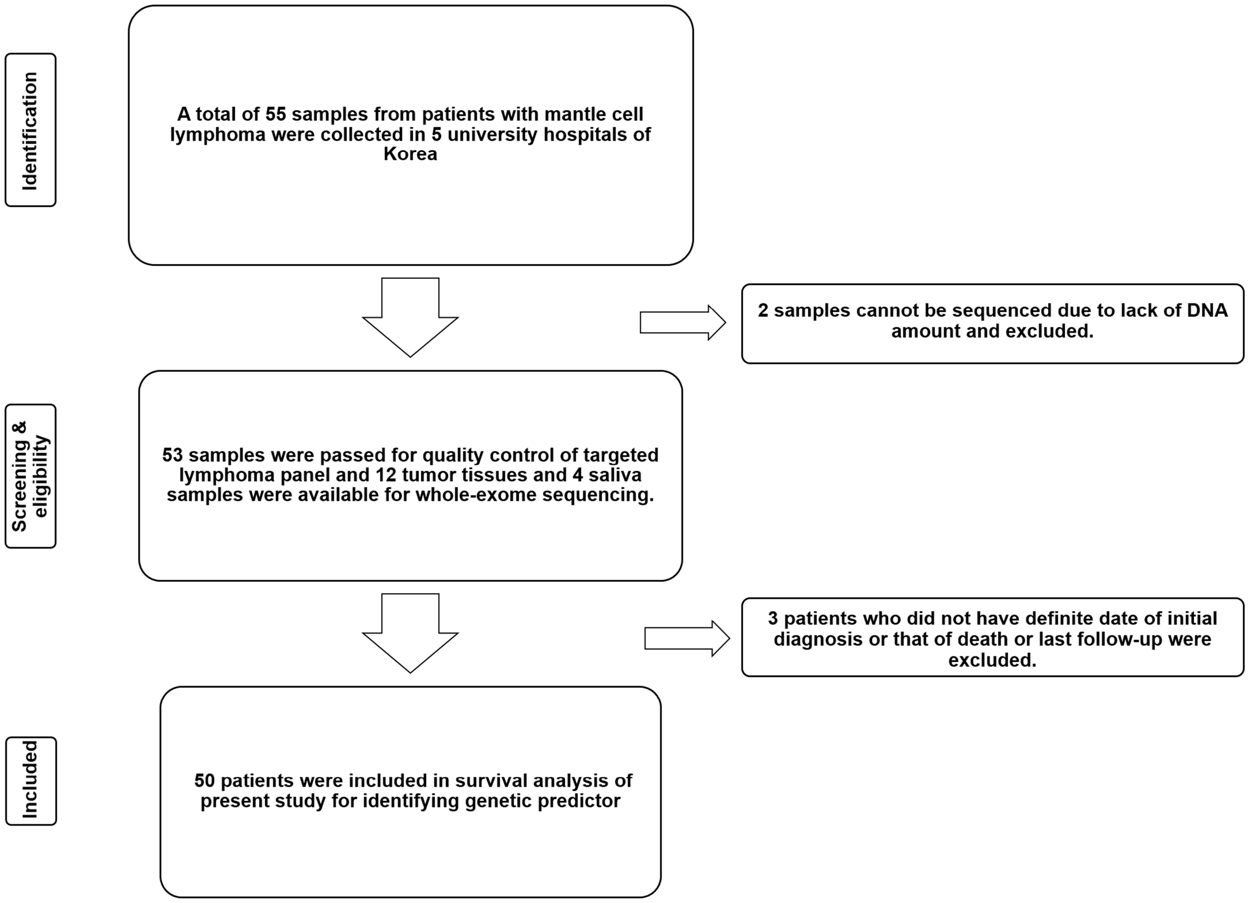

Patient	Age	Sex	Ki-67 (%)	β2-MG	LDH	Cytogenetic aberration	MIPI	IPI	Initial treatment*	ASCT	Radiotherapy	Efficacy	Sample status	OS (months)	PFS (months)	Cause of death
MCL2	40	M	70	Elevated	Elevated	Normal	4	4	R-CHOP	Yes	No	PR	Relapse	11	7	NA
MCL3	46	M	NA	Normal	Normal	Normal	1	2	R-CHOP	No	Yes	PR	Initial	117	NA	NA
MCL4	70	M	NA	Elevated	Normal	Normal	3	3	R-CHOP	No	No	NA	NA	36	NA	Pneumonia
MCL5	63	M	NA	Elevated	Normal	Normal	NA	2	R-FC	No	No	PR	NA	16	NA	Pneumonia
MCL6	64	M	60	Elevated	Elevated	Normal	7	3	R-hyper-CVAD	No	No	CR	Initial	82	NA	NA
MCL7	41	M	50	Elevated	Elevated	Normal	2	0	R-hyper-CVAD	No	No	CR	Initial	53	NA	NA
MCL8	51	M	NA	Normal	Elevated	Normal	NA	2	R-hyper-CVAD	Yes	No	PR	Initial	48	NA	NA
MCL9	68	F	NA	Normal	Normal	Normal	4	2	R-CHOP	No	No	NA	NA	2	NA	NA
MCL10	74	F	NA	Elevated	Normal	Normal	5	3	R-CHOP	No	No	NA	Initial	3	NA	Pneumonia
MCL11	56	M	40	Normal	Normal	Normal	6.5	5	R-CHOP	Yes	No	CR	Initial	30	NA	NA
MCL12	72	F	40	Elevated	Normal	Normal	7	2	CHOP	No	No	PR	Initial	32	NA	Respiratory failure
MCL13	70	M	NA	Elevated	Normal	Normal	8.6	2	R-CHOP	No	Yes	PR	Relapse	30	10	General weakness
MCL14	63	F	70	Elevated	Elevated	Normal	7.3	3	R-CHOP	No	Yes	CR	Relapse	27	22	Plasma cell myeloma
MCL15	76	M	NA	Normal	Normal	Normal	NA	3	CVP	No	No	PD	Relapse	124	60	NA
MCL16	62	M	NA	Normal	Normal	Normal	8.4	1	R-CHOP	No	Yes	CR	Relapse	157	153	Gallbladder cancer with hepatic metastasis
MCL17	55	M	NA	Elevated	Elevated	Normal	NA	2	R-hyper-CVAD	No	No	PD	Relapse	17	5	NA
MCL18	68	M	10	Normal	Elevated	Normal	NA	NA	CVP	No	No	NA	NA	9	NA	Liver cirrhosis and gastric varix with bleeding
MCL20	50	M	NA	Normal	Normal	Normal	3	1	R-hyper-CVAD	Yes	No	CR	Initial	106	NA	NA
MCL21	67	F	50	Normal	Normal	Normal	6.6	1	DHAP	No	No	CR	Relapse	16	9	Pneumonia
MCL22	67	F	NA	Elevated	Elevated	Normal	5	4	R-CHOP	No	No	PD	Relapse	9	8	NA
MCL23	69	F	90	Normal	Elevated	Normal	5	NA	R-CHOP	No	No	CR	Initial	11	NA	Pneumonia

MCL24	49	M	20	Normal	Elevated	Normal	NA	NA	R-CHOP	No	No	SD	Relapse	56	20	NA
MCL25	62	M	5	Elevated	Elevated	t(11;14)<br>(q13;q3+8;t(8;14))	NA	NA	R-CHOP	No	No	SD	Relapse	47	NA	NA
MCL26	75	M	NA	Elevated	Elevated	Normal	9.3	4	R-CHOP	No	No	PD	Relapse	20	19	Recurrent pleural effusion
MCL28	58	M	60	Normal	Elevated	Normal	7.1	0	CHOP	No	No	CR	Initial	40	NA	NA
MCL29	76	M	70	Normal	Normal	t(11;14)<br>(q13;q3+8;t(8;14))	7.2	4	FCM	No	No	PD	Relapse	21	13	Pneumonia aggravation
MCL30	49	M	60	Elevated	Normal	Normal	NA	NA	R-CHOP	No	No	CR	Relapse	13	12	NA
MCL31	40	M	10	Normal	Elevated	+8;t(8;14)	NA	NA	No	No	No	NA	NA	0	NA	NA
MCL32	61	M	NA	Normal	Normal	Normal	NA	2	CHOP	No	No	CR	Relapse	59	85	NA
MCL33	69	F	90	Elevated	Elevated	Normal	5	3	R-CHOP	No	No	NA	Initial	11	NA	Pneumonia
MCL34	71	M	NA	Elevated	Elevated	Normal	5	3	R-CEOP	No	No	NA	NA	7	NA	NA
MCL35	69	F	NA	Elevated	Normal	Normal	5	2	CHOP	No	No	PR	Relapse	46	36	Septic shock
MCL36	58	M	30	Normal	Normal	Normal	NA	NA	R-hyper-CVAD	Yes	No	CR	Relapse	70	22	NA
MCL37	59	F	40	Normal	Elevated	Normal	4	3	R-CHOP	No	No	CR	Relapse	29	20	NA
MCL38	77	M	70	Elevated	Normal	t(11;14)<br>(q13;q3+8;t(8;14))	5.9	3	VR-CAP	No	No	CR	NA	10	NA	NA
MCL39	77	F	20	Normal	Normal	Normal	6.7	NA	R-CHOP	No	No	CR	NA	34	NA	NA
MCL40	55	M	35	Elevated	Elevated	Normal	5.6	2	R-CHOP	Yes	No	PR	NA	6	NA	NA
MCL41	62	F	30	Normal	Elevated	Normal	2	3	R-hyper-CVAD	Yes	No	CR	Relapse	36	17	NA
MCL42	68	M	25	Normal	Normal	Normal	4	3	R-CHOP	No	No	NA	NA	0	NA	NA
MCL43	72	M	2	Normal	Normal	Normal	5.9	3	R-CHOP	No	Yes	CR	NA	71	NA	NA
MCL44	61	M	10	Elevated	Elevated	t(11;14)<br>(q13;q3+8;t(8;14))	6.3	3	R-hyper-CVAD	No	No	CR	NA	36	NA	NA
MCL45	62	M	15	Normal	Normal	Normal	5.6	3	R-CHOP	Yes	No	PR	NA	83	NA	NA

MCL46	70	M	10	Elevated	Normal	t(11;14)(q13;q3+8;t(8;14))	5.7	3	R-CHOP	No	No	PR	NA	44	NA	NA
MCL47	62	F	2	Normal	Normal	Normal	5	2	B-R	No	No	CR	NA	56	NA	NA
MCL48	57	M	80	Normal	Elevated	Normal	5	1	R-hyper-CVAD	No	No	CR	Initial	20	NA	Liver failure
MCL49	60	M	NA	Elevated	Normal	Normal	NA	3	R-CHOP	No	No	NA	Relapse	68	56	NA
MCL50	73	F	60	Normal	Elevated	Normal	5	3	R-CHOP	No	No	NA	Initial	16	NA	NA
MCL51	54	F	40	Normal	Normal	Normal	5.3	1	R-CHOP	Yes	No	CR	NA	92	NA	NA
MCL52	70	M	50	Elevated	Normal	Normal	5.7	2	B-R	No	No	CR	NA	51	NA	NA
MCL53	58	M	10	Elevated	Normal	Normal	5.4	2	R-hyper-CVAD	No	No	PR	NA	50	NA	NA

Supplementary Table S1. Clinical data and sample information of mantle cell lymphoma patients in our cohort. *B-R, bendamustine, and rituximab; CHOP, cyclophosphamide, doxorubicin, vincristine, and prednisolone; CVP, cyclophosphamide, vincristine, and prednisolone; DHAP, dexamethasone, high-dose ara-C cytarabine, and cisplatine; FCM, fludarabine, cyclophosphamide, and mitoxantrone; R-CEOP, rituximab, cyclophosphamide, etoposide, and prednisolone; R-CHOP, rituximab, cyclophosphamide, doxorubicin, vincristine, and prednisolone; R-hyper CVAD rituximab, cyclophosphamide, doxorubicin, vincristine, and dexamethasone alternating with rituximab plus high-dose methotrexate and cytarabine; VR-CAP, rituximab, bortezomib, cyclophosphamide, vincristine, and prednisone. ASCT, autologous stem cell transplantation; CR, complete remission; IPI, international prognostic index; LDH, lactate dehydrogenase; MG, macroglobulin; MIPI, mantle cell lymphoma international prognostic index; NA, not applicable; OS, overall survival; PD, progressive disease; PFS, progression free survival; PR, partial remission; SD, stable disease.

Variable	Kridel et al. 2012	Beà et al. 2013	Greiner et al. 2006	Zhang et al. 2014	Rahal et al. 2014	Meissner et al. 2013	Rossi et al. 2013	Yang et al. 2018	Ferrero et al. 2019	Our study
Studied number	108	29/172	92	56	165	102	151	16	300	53
Age, mean years	65	67/66	NA	NA	NA	64	< 65	63	18-65	63
Treatment with rituximab (%)	46	73/40	NA	NA	NA	NA	100	81	100	82
Studied country	Canada	Germany and Spain	Norway, Spain, Canada, and USA	USA	Germany, Switzerland, Canada, and USA	Canada	Italy	China	Denmark, Italy, Norway, Portugal, Sweden, and Switzerland	Korea
Mutation frequency (%)										
ATM	15	41	56	42	50	50	42	38	42	34
BIRC3		6		9	8		5		6	11
CCND1	19	35		16	19	19	14	19	12	13
KMT2D		14		20			12		12	32
NOTCH1	12	5		14	14	14	6		8	11
TP53	25		26	20	14	14	7	31	8	17
TRAF2					7		1		1	6
UBR5				7	18	18				9
WHSC1		10	7				13	31	17	13

Supplementary Table S2. Comparison with other mantle cell lymphoma studies for recurrently mutated genes.

Pathway name	Entities				Reactions	
	Found	Ratio	*P*-value	FDR	Found	Ratio
Regulation of TP53 expression	2/4	<0.001	<0.001	0.003	5/5	<0.001
TP53 regulates transcription of DNA repair genes	4/89	0.006	<0.001	0.003	15/17	0.001
Regulation of TP53 expression and degradation	3/46	0.003	<0.001	0.008	19/35	0.003
Transcriptional regulation by TP53	6/485	0.034	<0.001	0.019	177/259	0.021
Regulation of TP53 activity	4/178	0.013	<0.001	0.019	68/98	0.008
Generic transcription pathway	10/1,524	0.108	<0.001	0.020	196/811	0.066
TP53 regulates transcription of caspase activators and caspases	2/20	0.001	<0.001	0.023	13/17	0.001
Regulation of TP53 activity through phosphorylation	3/95	0.007	<0.001	0.023	24/26	0.002
Regulation of TP53 activity through methylation	2/23	0.002	0.001	0.023	9/12	<0.001
RNA polymerase II transcription	10/1,663	0.117	0.001	0.024	196/872	0.071
TP53 regulates transcription of genes involved in cytochrome C release	2/33	0.002	0.002	0.038	21/25	0.002
Gene expression (trnascription)	10/1,821	0.129	0.002	0.038	196/983	0.081
Activation of BH3-only proteins	2/36	0.003	0.003	0.038	5/19	0.002
Regulation of TP53 degradation	2/43	0.003	0.004	0.048	14/30	0.002
Deactivation of the beta-catenin transactivating complex	2/44	0.003	0.004	0.048	3/14	0.001
PKMTs methylate histone lysines	2/49	0.003	0.005	0.055	3/22	0.002
Autodegradation of the E3 ubiquitin ligase COP1	2/54	0.004	0.006	0.065	2/5	<0.001
Stabilization of p53	2/59	0.004	0.007	0.065	10/15	0.001
Intrinsic pathway for apoptosis	2/59	0.004	0.007	0.065	5/60	0.005

Recruitment and ATM-mediated phosphorylation of repair and signaling proteins at DNA double strand breaks	2/66	0.005	0.008	0.067	31/35	0.003
Regulation of cortical dendrite branching	1/4	<0.001	0.008	0.067	2/2	<0.001
Formation of the beta-catenin:TCF transactivating complex	2/67	0.005	0.008	0.067	13/13	0.001
p53-dependent G1 DNA damage response	2/70	0.005	0.009	0.067	13/22	0.002
p53-dependent G1/S DNA damage checkpoint	2/70	0.005	0.009	0.067	13/22	0.002
DNA damage/telomere stress induced senescence	2/71	0.005	0.009	0.067	11/18	0.001

Supplementary Table S3. Most significant 25 pathways sorted by *P*-values. FDR, false discovery rate.

Gene name											
ABCA13	*BCL2L11*	*CEBPA*	*DNAJC6**	*FBXW7*	*IDH2*	*LGALS16*	*MYO18A*	*PDLIM3**	*RELN**	*SLC3A2*	*TNFSF9*
ABCA3	*BCL6**	*CHD1*	*DNMT3A*	*FES*	*IFNA14*	*LIFR*	*MYOM2*	*PDZD7*	*RERE*	*SLITRK2*	*TNRC6B**
ABCA6	*BCL7A*	*CHD2*	*DNMT3B*	*FGFR1*	*IFNA4*	*LMCD1*	*NAV3*	*PER1*	*REV3L*	*SLITRK3*	*TOP2A*
*ABCA7**	*BCL9*	*CHD8*	*DOCK4*	*FGL2*	*IGLL5**	*LRIG3*	*NBN*	*PHF6*	*RFTN1*	*SLITRK5*	*TP53**
*ABCC9**	*BCOR*	*CHEK1**	*DOCK8*	*FIGN*	*IKBKB**	*LRP1B**	*NCOR1*	*PHLPP1*	*RGS4**	*SLITRK6*	*TRAF2**
*ABL1**	*BCORL1*	*CHEK2**	*DPCR1*	*FLG*	*IKZF1*	*LRRK1**	*NCOR2**	*PIAS2*	*RGS7*	*SMARCA2*	*TRAF3**
ACAD9	*BCR*	*CHKB*	*DPPA2*	*FLT3*	*IKZF3*	*LRRN3*	*NELL2*	*PIGN*	*RHBDD1*	*SMARCA4**	*TRIML2*
*ACTB**	*BIRC3**	*CHMP4C*	*DSC1*	*FLT4*	*IL2RB*	*LRTM1**	*NF1**	*PIK3CB*	*RHOA*	*SMC1A*	*TRPM6*
ACTG1	*BLM*	*CHPF2*	*DSEL*	*FLYWCH1*	*IL6*	*LUZP4*	*NFKBIA*	*PIK3CD*	*RIMS2**	*SMC3*	*TRRAP**
ACTN2	*BRAF*	*CHST9*	*DST*	*FOXO1*	*IL6R*	*LYN*	*NFKBIE**	*PIK3R1*	*RIPK1*	*SMYD1*	*TSHZ2*
ACTN3	*BRCA2**	*CHSY3**	*DTX1*	*FOXP1*	*IL7R*	*MAGT1*	*NFKBIZ*	*PIM1*	*RIPK2*	*SNX29*	*TTC27*
ACTN4	*BRCC3*	*CIITA*	*DUSP2*	*FTCD*	*IQCG*	*MAML1*	*NIN**	*PKD1**	*RNF213*	*SOCS1*	*TUBB*
ADD2	*BRD2*	*CLCN6*	*DYNC1H1*	*FUBP1*	*IRF2BP1*	*MAP1B**	*NIPBL*	*PKHD1L1**	*RNF40*	*SOHLH2*	*TYK2*
ADGRL2	*BRD4*	*CLGN*	*DYRK1A*	*FYB*	*IRF4*	*MAP2*	*NLRC5*	*PKM*	*ROBO2**	*SP140**	*TYRP1*
ADGRV1	*BRINP2*	*CMYA5*	*EBF1**	*GATA1*	*IRF8*	*MAP2K1*	*NOA1*	*PLCG1*	*RP1*	*SPEN**	*U2AF1*
AFAP1L2	*BRIP1*	*CNKSR2*	*ECT2L*	*GATA2*	*ITK*	*MAP3K14**	*NOL9*	*PLCG2*	*RP1L1*	*SPINK13*	*U2AF2**
AFF2	*BTG1*	*COL11A1**	*EEF1A1*	*GLI3*	*ITPKB*	*MAPK1*	*NOTCH1**	*PLEKHG1*	*RPS15*	*SRRM2*	*UBE2A*
AKT1	*BTG2*	*COL11A2*	*EEF1E1*	*GLIS3*	*ITPR1*	*MBD1*	*NOTCH2**	*PLEKHG5*	*RPS6KA1*	*SRSF2*	*UBR5**
ALDH7A1	*BTK*	*COL14A1**	*EGR2*	*GNA13*	*ITPR2*	*MCRS1*	*NPAT*	*PLXNA1*	*RRAGC*	*SRSF8*	*ULK4*
ALMS1	*CA4*	*COL16A1**	*EIF2AK4*	*GNAS*	*JAK1*	*MED12*	*NPM1*	*PLXNB3**	*RUBCN*	*STAG2*	*USH1C*
ALPK2	*CACNA1E*	*COL4A2*	*EIF4A1*	*GNAT1*	*JAK2*	*MED23*	*NPTX2*	*PMS2*	*RUNX1*	*STARD8*	*USP25*

ANK2*	CACNA2D3	COL6A3*	EIF4A2	GNB1	JAK3	MEF2B*	NRAS	POLG	RYR1*	STAT3	VAPA
ANKRD17*	CAD*	CPA4	ELF4	GON4L*	KANK2	MEF2C	NRXN3	POP1	S1PR1*	STAT5B	VPS13A
ANKRD44	CAMTA1	CREBBP*	ELP2	GPR37	KAT6B	MEIS1	NSD1	POSTN	S1PR2	STAT6*	WAC
AOC2	CAPN1	CREG2	EMC8	GPR65	KCNC2*	MFHAS1	NXF1	POT1	SALL3	STXBP2	WAS
APC*	CARD11*	CRYBG3*	EP300	GPS2	KCNH2	MGA	OCEL1	PPIG	SAMD9	SUZ12	WDR66
AQP2	CARD6*	CSF1R	EPHA3	GRIA2	KCNH6	MGAM	OGDHL	PPM1D	SAMHD1	SWAP70	WDR70
ARHGAP28	CASP10	CSF3R	EPOR	GRIN2A*	KCNQ3	MIB1	OR10A2	PPP1R9A	SBDS	SYK	WDR90
ARHGAP32	CBL	CTCF	ERAP1	GTSE1	KDM4C*	MIR142	OR1S2	PRAME	SCG3	SYNE1*	WHAMM
ARID1A*	CCM2L	CTNNA2	ERG	HDAC6	KDM5C	MKI67*	OR5H1	PRDM1*	SCRIB	TAF1	WHSC1*
ARID1B*	CCND1*	CUL1	ESF1	HEATR1	KDM6A	MKLN1	P2RY8	PRDM15	SENP6	TBC1D26*	WT1
ARID2	CCND3*	CUL9	ESX1	HEPH*	KDM6B	MLH1	PABPC1	PRDM2*	SETBP1	TBL1XR1*	XPO1
ARID5B*	CCR4	CXCR4	ETS1	HERC1	KIAA0355	MMP8	PABPC5	PRF1	SETD1A	TBX3	XPO4
ASXL1	CD14	DAP3	ETV6	HFE2	KIAA1671	MPDZ	PALLD	PRKCB	SETD1B	TCF12	YLPM1*
ATAD2B	CD27	DCAF4L2	EVI2A	HIST1H1B	KIR2DL4	MPEG1	PAMR1	PRKD3	SETD2*	TCF3*	YY1AP1
ATM*	CD274	DCDC2B	EWSR1	HIST1H1C	KIR3DL1	MPL	PASD1	PROS1	SETD6	TCTN2	ZBTB9
ATMIN	CD28	DCDC5	EXOSC6	HIST1H1D	KIT	MRGPRF	PASK	PRPF40B	SF1	TET1*	ZEB1
ATP10B*	CD40LG	DCP1B	EZH2*	HIST1H1E	KLF2	MSH2	PAX6	PTEN	SF3A1	TET2	ZFHX3*
ATP11C	CD58	DDX3X	FAM205A	HIST1H2AC	KLHDC8B	MSH6	PBXIP1	PTGFRN	SF3B1	TFCP2	ZFP36L1
ATP6AP1	CD70	DGKB*	FAM46C	HIST1H2AG	KLHL14	MSL2	PCBP1	PTPN11	SGK1	TGM7*	ZMYM3
ATP6V1B2	CD79A	DHCR7*	FAM50A	HIST1H2BC	KLHL6	MSN	PCDHB2	PTPN14	SH2B3	THBS1	ZNF117*
ATR	CD79B	DHDH	FANCD2	HIST1H2BD	KMT2A	MTOR*	PCDHGA12	PTPRCAP	SH2D1A	TLN2	ZNF296
ATRX*	CD83	DIS3	FAS*	HIST1H2BG	KMT2C*	MUC16*	PCLO*	PTPRD	SI*	TLR2	ZNF429
B2M	CDC123	DLC1*	FASLG	HIST1H2BK	KMT2D*	MUM1	PCSK2	PTPRN	SIN3A	TMSB4X	ZNF521
BARD1	CDH11	DLEU2	FAT1*	HIVEP2	KRAS	MYBBP1A	PDCD11	RAD21	SLC13A3	TMTC2	ZNF608

BAZ2A	*CDH23*[*]	*DLGAP2*	*FAT4*[*]	*HOXB2*[*]	*LAMA2*	*MYC*	*PDCD1LG2*	*RAG2*	*SLC16A7*	*TNFAIP3*[*]	*ZNF708*
BCL10[*]	*CDH8*[*]	*DMD*	*FBXO11*	*HUWE1*	*LAMB4*	*MYD88*	*PDCL*	*RB1*[*]	*SLC17A6*	*TNFRSF13B*	*ZNF85*
BCL11B	*CDKN2A*	*DMXL1*	*FBXO21*	*ID3*	*LCN2*	*MYH11*	*PDGFC*	*RBM23*	*SLC29A2*[*]	*TNFRSF14*[*]	*ZNF90*
BCL2[*]	*CDKN2B*	*DNAH5*	*FBXO42*	*IDH1*	*LEP*	*MYH9*	*PDGFRB*	*RBMXL3*	*SLC30A6*	*TNFRSF1B*	*ZRSR2*[*]

Supplementary Table S4. The 588 gene list of targeted lymphoma panel set used for next-generation sequencing experiments. [*]Genes identified by targeted panel sequencing and whole-exome sequencing concurrently.

Sample	Average read depth	Coverage of target, >30X, %
MCL1	86.8	96.7
MCL2	378	99.8
MCL3	259	99.7
MCL4	316.9	99.8
MCL5	123.5	97.7
MCL6	718	99.9
MCL7	507.4	99.9
MCL8	384.2	99.9
MCL9	381	97.3
MCL10	745.8	99.9
MCL11	119.8	95.0
MCL12	145.4	99.2
MCL13	365	99.7
MCL14	231.8	99.1
MCL15	197.9	98.1
MCL16	79.8	95.7
MCL17	126.6	96.5
MCL18	92.6	85.8
MCL19	145.1	93.2
MCL20	392.5	99.8
MCL21	93.6	94.2

MCL22	181.1	98.7
MCL23	547.9	99.9
MCL24	401.7	95.8
MCL25	393.8	96.5
MCL26	391.1	95.8
MCL27	277.7	93
MCL28	527.9	99.9
MCL29	568.5	99.7
MCL30	272.7	82.3
MCL31	724	96.7
MCL32	451.8	99.8
MCL33	152.8	99.7
MCL34	122.5	99.2
MCL35	76.9	93.2
MCL36	72.4	94.2
MCL37	269.7	99.9
MCL38	184.2	99.7
MCL39	270.4	99.8
MCL40	121.2	97.7
MCL41	111.2	89.7
MCL42	845.7	99.9
MCL43	810.8	99.9
MCL44	1000.5	99.9
MCL45	844	99.9

MCL46	712.9	99.9
MCL47	322	99.8
MCL48	458.2	99.9
MCL49	121.2	99.6
MCL50	539.8	96.7
MCL51	584	99.9
MCL52	704.1	99.9
MCL53	441.5	99.9

Supplementary Table S5. Read depth and coverage of targeted lymphoma panel sequencing.

Sample	Average read depth	Coverage of target, >30X, %
MCL8	145.2	40.7
MCL8C	159.9	43.7
MCL41	151.7	26.3
MCL41C	146.5	45.5
MCL50	154.4	40.1
MCL50C	132.4	45.2
MCL18	131.0	27.0
MCL12	142.8	24.9
MCL6	137.9	37.5
MCL4	139.7	34.8
MCL26	136.0	33.5
MCL7	144.8	32.7
MCL29	159.7	31.3
MCL37	133.9	39.3
MCL11	125.5	42.3
MCL49C	161.8	47.5

Supplementary Table S6. Read depth and coverage of whole-exome sequencing.

Sample	Gene	HGVS cDNA	HGVS protein	VAF	Total depth	Variant depth	SIFT_prediction	Polyphen2_prediction	Method
MCL1	*CXCR4*	c.1034_1035delAG	p.Glu345ValfsTer12	0.400	55	22			TPS
MCL1	*KDM6A*	c.514C>T	p.Arg172Ter	0.148	54	8			TPS
MCL1	*ACTB*	c.826G>A	p.Glu276Lys	0.214	28	6		B	TPS
MCL1	*ARID2*	c.2428G>C	p.Ala810Pro	0.545	55	30	D	B	TPS
MCL1	*EZH2*	c.638G>A	p.Arg213His	0.500	134	67	T	B	TPS
MCL1	*FOXP1*	c.174_176delGCA	p.Gln60del	0.100	90	9			TPS
MCL1	*ABCA13*	c.12242_12244delAGG	p.Glu4081del	0.114	35	4			TPS
MCL1	*DNAJC6*	c.473C>T	p.Ser158Leu	0.163	43	7	D	D	TPS
MCL1	*LRP1B*	c.3799C>T	p.Arg1267Cys	0.143	56	8	D	D	TPS
MCL1	*SETD2*	c.4997A>G	p.Tyr1666Cys	0.622	90	56	D	D	TPS
MCL1	*CTNNA2*	c.2397G>T	p.Lys799Asn	0.403	72	29	D	B	TPS
MCL1	*COL11A2*	c.2650C>T	p.Pro884Ser	0.233	30	7	D	B	TPS
MCL1	*SYNE1*	c.9691C>T	p.His3231Tyr	0.656	64	42	T	B	TPS
MCL1	*SYNE1*	c.5476G>A	p.Glu1826Lys	0.325	77	25	T	P	TPS
MCL1	*SYNE1*	c.1381C>A	p.Gln461Lys	0.111	54	6	T	B	TPS
MCL1	*RIMS2*	c.1693-18G>T		0.154	26	4			TPS
MCL1	*PKHD1L1*	c.9090-3C>T		0.238	21	5			TPS
MCL1	*CREG2*	c.502A>G	p.Thr168Ala	0.496	111	55	D	D	TPS
MCL1	*SYNE1*	c.5585A>T	p.Glu1862Val	0.563	64	36	D	D	TPS
MCL1	*SYNE1*	c.5584G>A	p.Glu1862Lys	0.587	63	37	D	D	TPS
MCL1	*RBMXL3*	c.818G>A	p.Arg273His	0.952	42	40	T	D	TPS
MCL2†	*KMT2D*	c.11713C>T	p.Gln3905Ter	0.185	146	27			TPS
MCL2†	*EZH2*	c.1936T>A	p.Tyr646Asn	0.179	507	91	D	D	TPS

MCL2†	*JAK1*	c.3230dupA	p.Tyr1077Ter	0.171	551	94			TPS
MCL2†	*PRDM1*	c.1551delC	p.Thr518ArgfsTer30	0.201	189	38			TPS
MCL2†	*KCNH6*	c.2314delG	p.Ala772GlnfsTer15	0.424	59	25			TPS
MCL2†	*NCOR2*	c.705+4T>C		0.448	451	202			TPS
MCL2†	*TMSB4X*	c.100+5G>C		0.365	249	91			TPS
MCL2†	*FLYWCH1*	c.49G>A	p.Gly17Ser	0.551	450	248	T	B	TPS
MCL2†	*DHDH*	c.487C>T	p.Arg163Trp	0.525	198	104	D	D	TPS
MCL2†	*IGLL5*	c.115C>G	p.Leu39Val	0.176	238	42	D	B	TPS
MCL2†	*CRYBG3*	c.127G>T	p.Ala43Ser	0.415	323	134		D	TPS
MCL2†	*CRYBG3*	c.1511C>G	p.Thr504Arg	0.559	426	238		D	TPS
MCL2†	*CRYBG3*	c.3268A>C	p.Lys1090Gln	0.507	505	256		B	TPS
MCL2†	*CHSY3*	c.1220A>G	p.Asn407Ser	0.454	205	93	T	B	TPS
MCL2†	*LAMB4*	c.1985C>T	p.Ala662Val	0.485	328	159	T	P	TPS
MCL3*	*TET2*	c.1630C>T	p.Arg544Ter	0.227	357	81			TPS
MCL3*	*TET2*	c.1530delA	p.Glu510AspfsTer23	0.191	236	45			TPS
MCL3*	*BARD1*	c.258T>A	p.Cys86Ter	0.463	229	106			TPS
MCL3*	*COL6A3*	c.5642G>A	p.Arg1881His	0.466	706	329	D	D	TPS
MCL3*	*TET1*	c.3941C>T	p.Ala1314Val	0.426	237	101	D	B	TPS
MCL3*	*KMT2A*	c.137T>G	p.Val46Gly	0.350	20	7	T	B	TPS
MCL3*	*YLPM1*	c.3792G>T	p.Trp1264Cys	0.508	370	188		D	TPS
MCL3*	*COL16A1*	c.3451G>A	p.Asp1151Asn	0.429	77	33	T	D	TPS
MCL3*	*TLN2*	c.161C>T	p.Ser54Leu	0.450	218	98	T	D	TPS
MCL3*	*HERC1*	c.7654G>A	p.Val2552Ile	0.467	368	172	T	B	TPS
MCL3*	*LRRK1*	c.1805C>T	p.Thr602Ile	0.475	221	105	T	B	TPS

MCL3*	*NCOR1*	c.1670C>T	p.Thr557Ile	0.394	378	149	T	B	TPS
MCL3*	*CRYBG3*	c.7542C>G	p.His2514Gln	0.449	325	146		B	TPS
MCL3*	*SYNE1*	c.14669C>T	p.Ala4890Val	0.371	175	65	D	B	TPS
MCL3*	*PCLO*	c.5891C>T	p.Thr1964Met	0.305	154	47	D	P	TPS
MCL4	*KMT2D*	c.4747delC	p.Gln1583SerfsTer10	0.297	583	173			TPS
MCL4	*NFKBIE*	c.759_762delTTAC	p.Tyr254SerfsTer13	0.141	1065	150			TPS
MCL4	*ATM*	c.7336G>A	p.Glu2446Lys	0.365	126	46	T	D	TPS
MCL4	*ATM*	c.7340T>C	p.Leu2447Ser	0.394	132	52	D	D	TPS
MCL4	*TGM7*	c.404G>A	p.Gly135Glu	0.468	77	36	D	D	TPS
MCL4	*PKD1*	c.2102C>A	p.Thr701Asn	0.212	353	75	T	P	TPS
MCL4	*UBR5*	c.8187+3A>T		0.296	135	40			TPS
MCL4	*CAD*	c.2576C>T	p.Pro859Leu	0.483	656	317	D	P	TPS
MCL4	*SLC29A2*	c.1291G>A	p.Gly431Ser	0.431	390	168	D	D	TPS
MCL4	*KMT2D*	c.4682T>G	p.Val1561Gly	0.498	319	159	T	B	TPS
MCL4	*NIN*	c.5039A>C	p.Lys1680Thr	0.582	134	78	D	D	TPS
MCL4	*LRRK1*	c.4332+5G>A		0.292	298	87			TPS
MCL4	*CDH8*	c.2084T>C	p.Leu695Ser	0.538	117	63	T	B	TPS
MCL4	*ANKRD44*	c.1316+4T>C		0.514	210	108			TPS
MCL4	*ABCA13*	c.9200A>G	p.Asp3067Gly	0.518	85	44	D	B	TPS
MCL4	*ATM*	c.7336G>A	p.Glu2446Lys	0.465	43	20	T	D	WES
MCL4	*ATM*	c.7340T>C	p.Leu2447Ser	0.457	46	21	D	D	WES
MCL4	*BCL10*	c.657T>G	p.Ser219Arg	0.143	35	5	D	P	WES
MCL4	*BRCA2*	c.7835C>A	p.Pro2612Gln	0.108	37	4	D	D	WES
MCL4	*CAD*	c.2576C>T	p.Pro859Leu	0.438	258	113	D	P	WES

MCL4	*CDH8*	c.2084T>C	p.Leu695Ser	0.600	45	27	T	B	WES
MCL4	*DLC1*	c.762_763delinsCG	p.GlnAsn254HisAsp	0.417	24	10			WES
MCL4	*IGF2BP2*	c.1201G>A	p.Gly401Arg	0.160	25	4	D	D	WES
MCL4	*KMT2C*	c.943_946delinsAGCT	p.GlyThr315SerSer	0.108	307	33			WES
MCL4	*KMT2D*	c.4747del	p.Gln1583SerfsTer10	0.288	177	51			WES
MCL4	*KMT2D*	c.4682T>G	p.Val1561Gly	0.511	88	45	T	B	WES
MCL4	*LRRK1*	c.5813_5814delinsAA	p.Gly1938Glu	0.419	179	75			WES
MCL4	*MPDZ*	c.2810C>T	p.Ala937Val	0.471	17	8	T	B	WES
MCL4	*NFKBIE*	c.759_762del	p.Tyr254SerfsTer13	0.191	314	60			WES
MCL4	*NIN*	c.5039A>C	p.Lys1680Thr	0.482	85	41	D	D	WES
MCL4	*ROBO2*	c.73_75delinsATC	p.Val25Ile	0.205	88	18			WES
MCL4	*SLC29A2*	c.1291G>A	p.Gly431Ser	0.471	85	40	D	D	WES
MCL4	*SPEN*	c.7135G>A	p.Glu2379Lys	0.103	39	4	D	D	WES
MCL4	*TGM7*	c.404G>A	p.Gly135Glu	0.452	31	14	D	D	WES
MCL4	*TUBA1B*	c.859T>A	p.Ser287Thr	0.236	55	13		B	WES
MCL4	*ZNF117*	c.566A>G	p.Glu189Gly	0.222	18	4	T	B	WES
MCL5	*SAMHD1*	c.490C>T	p.Arg164Ter	0.516	64	33			TPS
MCL5	*KMT2A*	c.5941delC	p.Arg1981GlyfsTer3	0.097	62	6			TPS
MCL5	*HERC1*	c.5526_5527delGG	p.Asp1843SerfsTer4	0.092	65	6			TPS
MCL5	*KDM6B*	c.2281_2287delACCACGG	p.Thr761ProfsTer19	0.092	109	10			TPS
MCL5	*ADGRV1*	c.1525C>T	p.Gln509Ter	0.182	55	10			TPS
MCL5	*PPP1R9A*	c.3920delG	p.Gly1307GlufsTer2	0.140	57	8			TPS
MCL5	*MEF2C*	c.68A>G	p.Lys23Arg	0.487	150	73	D	D	TPS
MCL5	*ATM*	c.6503C>T	p.Ser2168Leu	0.523	86	45	D	D	TPS

MCL5	*BCL11B*	c.578G>T	p.Gly193Val	0.405	568	230	T	B	TPS
MCL5	*FLYWCH1*	c.1354C>T	p.Arg452Trp	0.491	481	236	D	D	TPS
MCL5	*SYNE1*	c.12013-3C>T		0.200	30	6			TPS
MCL5	*CARD11*	c.1941-7G>T		0.424	238	101			TPS
MCL5	*TRPM6*	c.4673-7C>T		0.188	32	6			TPS
MCL5	*FBXO42*	c.1202C>A	p.Ala401Asp	0.568	81	46	T	B	TPS
MCL5	*DNAJC6*	c.1367A>G	p.Asn456Ser	0.584	89	52	T	B	TPS
MCL5	*MKI67*	c.802A>T	p.Thr268Ser	0.549	71	39	T	B	TPS
MCL5	*KCNC2*	c.1829T>A	p.Val610Glu	0.525	61	32	T	B	TPS
MCL5	*ABCA7*	c.1001G>A	p.Arg334Gln	0.569	123	70	T	B	TPS
MCL5	*WHSC1*	c.3295G>A	p.Glu1099Lys	0.327	98	32	D	D	TPS
MCL5	*FAT1*	c.3379A>G	p.Ile1127Val	0.468	47	22	T	B	TPS
MCL5	*ATP10B*	c.1324A>G	p.Lys442Glu	0.596	47	28	D	D	TPS
MCL5	*RIMS2*	c.1625-9C>T		0.524	21	11			TPS
MCL5	*COL14A1*	c.2004+7T>A		0.382	55	21			TPS
MCL5	*RBMXL3*	c.818G>A	p.Arg273His	0.988	81	80	T	D	TPS
MCL6*	*NOTCH2*	c.7198C>T	p.Arg2400Ter	0.342	570	195			TPS
MCL6*	*FOXP1*	c.1A>G	p.Met1?	0.519	751	390	D	P	TPS
MCL6*	*WDR66*	c.25C>T	p.Arg9Ter	0.478	502	240			TPS
MCL6*	*DHCR7*	c.907G>A	p.Gly303Arg	0.566	1152	652	D	D	TPS
MCL6*	*BRIP1*	c.1442G>A	p.Gly481Asp	0.528	951	502	T	D	TPS
MCL6*	*FOXP1*	c.301A>G	p.Met101Val	0.519	751	390	D	P	TPS
MCL6*	*AKT1*	c.1373T>C	p.Met458Thr	0.491	699	343	T	B	TPS
MCL6*	*MTOR*	c.3823G>T	p.Val1275Phe	0.782	925	723	D	D	TPS

MCL6*	*ATM*	c.3878A>G	p.Asn1293Ser	0.761	940	715	T	B	TPS
MCL6*	*ARID1A*	c.2161+35A>G		0.855	413	353			TPS
MCL6*	*TET1*	c.1588A>G	p.Ile530Val	0.466	472	220	T	B	TPS
MCL6*	*CDH11*	c.932A>G	p.Asp311Gly	0.500	662	331	T	D	TPS
MCL6*	*ACTN4*	c.1469A>G	p.Asn490Ser	0.516	820	423	T	B	TPS
MCL6*	*CARD6*	c.157C>A	p.Leu53Ile	0.420	1234	518	T	B	TPS
MCL6*	*PCLO*	c.3539T>C	p.Leu1180Pro	0.547	611	334	T	B	TPS
MCL6*	*PKHD1L1*	c.8877C>A	p.Asp2959Glu	0.510	814	415	D	B	TPS
MCL6*	*ARID1A*	c.5311C>T	p.Pro1771Ser	0.877	65	57	T	B	WES
MCL6*	*ATM*	c.3878A>G	p.Asn1293Ser	0.640	75	48	T	B	WES
MCL6*	*CARD6*	c.157C>A	p.Leu53Ile	0.388	103	40	T	B	WES
MCL6*	*DHCR7*	c.907G>A	p.Gly303Arg	0.586	162	95	D	D	WES
MCL6*	*EZH2*	c.1069C>T	p.Arg357Trp	0.108	37	4	D	D	WES
MCL6*	*KMT2C*	c.943_946delinsTGCT	p.GlyThr315CysSer	0.128	335	43			WES
MCL6*	*MTOR*	c.3823G>T	p.Val1275Phe	0.623	106	66	D	D	WES
MCL6*	*NOTCH2*	c.7198C>T	p.Arg2400Ter	0.386	127	49			WES
MCL6*	*PCDHB2*	c.2279_2280delinsAA	p.Gly760Glu	0.846	13	11			WES
MCL6*	*PCLO*	c.3539T>C	p.Leu1180Pro	0.385	26	10	T	B	WES
MCL6*	*PKHD1L1*	c.8877C>A	p.Asp2959Glu	0.548	135	74	D	B	WES
MCL6*	*ROBO2*	c.73_75delinsATC	p.Val25Ile	0.156	307	48			WES
MCL6*	*SYNE1*	c.12149_12150delinsGT	p.Lys4050Ser	0.455	44	20			WES
MCL6*	*SYNE1*	c.5439del	p.His1813GlnfsTer8	0.118	34	4			WES
MCL6*	*TET1*	c.1588A>G	p.Ile530Val	0.397	63	25	T	B	WES
MCL6*	*TUBA1B*	c.859T>A	p.Ser287Thr	0.139	65	9		B	WES

MCL6*	*WEE1*	c.721A>C	p.Asn241His	0.466	58	27	T	P	WES
MCL6*	*ZNF117*	c.1091G>A	p.Gly364Glu	0.103	145	15	T	B	WES
MCL7*	*KMT2D*	c.12592C>T	p.Arg4198Ter	0.503	914	460			TPS
MCL7*	*EZH2*	c.1936T>A	p.Tyr646Asn	0.418	239	100	D	D	TPS
MCL7*	*CD79A*	c.538delG	p.Asp180MetfsTer12	0.234	64	15			TPS
MCL7*	*EBF1*	c.461T>A	p.Leu154Ter	0.261	115	30			TPS
MCL7*	*LRTM1*	c.1013_1014delAG	p.Glu338AlafsTer3	0.326	519	169			TPS
MCL7*	*COL6A3*	c.466G>T	p.Asp156Tyr	0.434	553	240	D	P	TPS
MCL7*	*STAT6*	c.1256A>G	p.Asp419Gly	0.618	633	391	D	D	TPS
MCL7*	*IRF8*	c.1271T>A	p.Ile424Asn	0.369	1119	413	D	B	TPS
MCL7*	*TNFRSF14*	c.286T>G	p.Cys96Gly	0.727	512	372	D	D	TPS
MCL7*	*SLC29A2*	c.278T>A	p.Val93Asp	0.406	478	194	D	B	TPS
MCL7*	*BCL2*	c.47T>A	p.Met16Lys	0.299	663	198	D	P	TPS
MCL7*	*FAT4*	c.14407C>T	p.Pro4803Ser	0.360	189	68	T	B	TPS
MCL7*	*EBF1*	c.454C>G	p.Arg152Gly	0.250	112	28	D	D	TPS
MCL7*	*TYK2*	c.2102G>C	p.Arg701Thr	0.525	1113	584	D	D	TPS
MCL7*	*GON4L*	c.2150C>T	p.Pro717Leu	0.450	535	241	D	B	TPS
MCL7*	*ABCA3*	c.4421G>A	p.Arg1474Gln	0.483	1786	862	T	P	TPS
MCL7*	*SOCS1*	c.359C>T	p.Ala120Val	0.486	902	438	T	B	TPS
MCL7*	*TRRAP*	c.1445C>G	p.Ala482Gly	0.473	448	212	T	B	TPS
MCL7*	*TRAF2*	c.500G>A	p.Arg167Gln	0.504	2486	1253	T	B	TPS
MCL7*	*PLXNB3*	c.1018G>A	p.Gly340Ser	1.000	2196	2195	T	B	TPS
MCL7*	*COL6A3*	c.466G>T	p.Asp156Tyr	0.550	40	22	D	P	WES
MCL7*	*DLC1*	c.762_763delinsCG	p.GlnAsn254HisAsp	0.576	66	38			WES

MCL7*	*EBF1*	c.454C>G	p.Arg152Gly	0.491	57	28	D	D	WES
MCL7*	*EBF1*	c.461T>A	p.Leu154Ter	0.483	60	29			WES
MCL7*	*EZH2*	c.1921T>A	p.Tyr641Asn	0.392	51	20	D	D	WES
MCL7*	*FAT4*	c.14407C>T	p.Pro4803Ser	0.567	30	17	T	B	WES
MCL7*	*GON4L*	c.2150C>T	p.Pro717Leu	0.447	47	21	D	B	WES
MCL7*	*KMT2D*	c.12592C>T	p.Arg4198Ter	0.483	147	71			WES
MCL7*	*KMT2D*	c.8073_8094dup	p.Thr2699SerfsTer32	0.283	173	49			WES
MCL7*	*KMT2D*	c.13885A>C	p.Thr4629Pro	0.281	32	9	D	D	WES
MCL7*	*LRTM1*	c.1013_1014del	p.Glu338AlafsTer3	0.268	138	37			WES
MCL7*	*PCDHB2*	c.2279_2280delinsAA	p.Gly760Glu	1.000	30	30			WES
MCL7*	*ROBO2*	c.73_75delinsATC	p.Val25Ile	0.286	189	54			WES
MCL7*	*SLC29A2*	c.278T>A	p.Val93Asp	0.442	52	23	D	B	WES
MCL7*	*STAT6*	c.1256A>G	p.Asp419Gly	0.639	72	46	D	D	WES
MCL7*	*STAT6*	c.1936A>C	p.Ile646Leu	0.268	82	22	T	B	WES
MCL7*	*SYNE1*	c.12149_12150delinsGT	p.Lys4050Ser	0.351	37	13			WES
MCL7*	*TNFRSF14*	c.286T>G	p.Cys96Gly	0.588	102	60	D	D	WES
MCL7*	*TRAF2*	c.500G>A	p.Arg167Gln	0.473	277	131	T	B	WES
MCL7*	*TRRAP*	c.1445C>G	p.Ala482Gly	0.485	171	83	T	B	WES
MCL7*	*TRRAP*	c.4874G>T	p.Arg1625Leu	0.445	1314	585	T	P	WES
MCL7*	*TRRAP*	c.203G>A	p.Arg68Gln	0.105	38	4	T	D	WES
MCL8*	*TP53*	c.832C>T	p.Pro278Ser	0.288	431	124	D	D	TPS
MCL8*	*KMT2D*	c.12027_12028delTT	p.Ser4010Ter	0.295	414	122			TPS
MCL8*	*KMT2D*	c.10570C>T	p.Gln3524Ter	0.251	362	91			TPS
MCL8*	*PCLO*	c.6463G>T	p.Glu2155Ter	0.252	298	75			TPS

MCL8*	*KMT2D*	c.11566C>A	p.Gln3856Lys	0.410	366	150	D	D	TPS
MCL8*	*FAS*	c.749G>A	p.Arg250Gln	0.276	199	55	D	D	TPS
MCL8*	*SI*	c.3424-12T>C		0.413	167	69			TPS
MCL8*	*NF1*	c.5608C>T	p.Arg1870Trp	0.241	374	90	D	D	TPS
MCL8*	*DNAJC6*	c.1415A>C	p.Gln472Pro	0.268	392	105	T	B	TPS
MCL8*	*KMT2A*	c.166T>G	p.Ser56Ala	0.333	42	14	D	B	TPS
MCL8*	*TBL1XR1*	c.529T>C	p.Trp177Arg	0.229	454	104	D	D	TPS
MCL8*	*TNFAIP3*	c.979G>C	p.Ala327Pro	0.395	570	225	D	D	TPS
MCL8*	*ABCA7*	c.5609G>T	p.Gly1870Val	0.449	652	293	D	D	TPS
MCL8*	*LRP1B*	c.5734G>A	p.Ala1912Thr	0.421	235	99	D	D	TPS
MCL8*	*BIRC3*	c.403C>T	p.Arg135Cys	0.490	302	148	D	P	TPS
MCL8*	*ABCA7*	c.2892C>G	p.Asp964Glu	0.373	656	245	D	D	TPS
MCL8*	*MUC16*	c.7871C>T	p.Thr2624Met	0.469	292	137	D	D	TPS
MCL8*	*CREG2*	c.502A>G	p.Thr168Ala	0.525	404	212	D	D	TPS
MCL8*	*IGLL5*	c.206+8G>A		0.339	310	105			TPS
MCL8*	*FAT4*	c.1244C>G	p.Pro415Arg	0.481	570	274	D	D	TPS
MCL8*	*MAP1B*	c.4903G>A	p.Val1635Ile	0.196	322	63	D	B	TPS
MCL8*	*RIMS2*	c.376C>T	p.Arg126Cys	0.235	277	65	D	D	TPS
MCL8*	*BCL2*	c.351C>A	p.Ser117Arg	0.197	304	60	T	B	WES
MCL8*	*DNAJC6*	c.1625A>C	p.Gln542Pro	0.286	56	16	T	B	WES
MCL8*	*FAS*	c.749G>A	p.Arg250Gln	0.256	82	21	D	D	WES
MCL8*	*HEPH*	c.1229G>A	p.Arg410Lys	0.108	37	4	T	B	WES
MCL8*	*IGLL5*	c.170G>A	p.Gly57Glu	0.203	69	14	D	B	WES
MCL8*	*KMT2D*	c.10570C>T	p.Gln3524Ter	0.257	140	36			WES

MCL8*	*KMT2D*	c.12027_12028del	p.Ser4010Ter	0.235	136	32			WES
MCL8*	*MAP1B*	c.4903G>A	p.Val1635Ile	0.202	124	25	D	B	WES
MCL8*	*MUC16*	c.36539C>G	p.Thr12180Ser	0.171	111	19	D	B	WES
MCL8*	*NF1*	c.5545C>T	p.Arg1849Trp	0.200	20	4	D	D	WES
MCL8*	*OGDHL*	c.1487G>A	p.Arg496His	0.178	107	19	D	D	WES
MCL8*	*PCLO*	c.6463G>T	p.Glu2155Ter	0.338	68	23			WES
MCL8*	*RIMS2*	c.376C>T	p.Arg126Cys	0.219	105	23	D	D	WES
MCL8*	*TBL1XR1*	c.529T>C	p.Trp177Arg	0.215	65	14	D	D	WES
MCL8*	*TNFAIP3*	c.979G>C	p.Ala327Pro	0.440	157	69	D	D	WES
MCL8*	*TP53*	c.832C>T	p.Pro278Ser	0.207	58	12	D	D	WES
MCL9	*BRAF*	c.1803A>T	p.Lys601Asn	0.534	103	55	D	P	TPS
MCL9	*SLC30A6*	c.786-1G>T		0.206	34	7			TPS
MCL9	*CMYA5*	c.868delC	p.Gln290LysfsTer3	0.108	37	4			TPS
MCL9	*MPEG1*	c.445C>T	p.Arg149Ter	0.597	62	37			TPS
MCL9	*CDH23*	c.8401T>G	p.Phe2801Val	0.559	1009	564	T	B	TPS
MCL9	*ARID1A*	c.6733G>A	p.Asp2245Asn	0.464	1779	825	D	P	TPS
MCL9	*PHLPP1*	c.1111G>A	p.Gly371Ser	0.524	1228	643	D		TPS
MCL9	*ANKRD17*	c.369_374delCGACGA	p.Asp123_Asp124del	0.325	117	38			TPS
MCL9	*APC*	c.204A>C	p.Leu68Phe	0.560	50	28	T	B	TPS
MCL9	*DDX3X*	c.1079A>G	p.Gln360Arg	0.859	71	61	D	D	TPS
MCL9	*TNRC6B*	c.871G>A	p.Asp291Asn	0.513	80	41	T	P	TPS
MCL9	*RP1*	c.2923A>G	p.Ile975Val	0.515	68	35	T	B	TPS
MCL10*	*RB1*	c.2267A>G	p.Tyr756Cys	0.237	523	124	D	D	TPS
MCL10*	*TET1*	c.733C>T	p.Gln245Ter	0.144	584	84			TPS

MCL10*	*B2M*	c.43_44delCT	p.Leu15PhefsTer41	0.213	1113	237			TPS
MCL10*	*BAZ2A*	c.2188_2189delAT	p.Ile730ProfsTer7	0.307	479	147			TPS
MCL10*	*RYR1*	c.9859C>T	p.Arg3287Cys	0.575	426	245	D	D	TPS
MCL10*	*CDH23*	c.7418T>C	p.Leu2473Pro	0.493	832	410	D	D	TPS
MCL10*	*SYNE1*	c.8578A>G	p.Thr2860Ala	0.491	922	453	D	B	TPS
MCL10*	*FOXO1*	c.71C>T	p.Thr24Ile	0.154	1002	154	D	D	TPS
MCL10*	*GON4L*	c.5918C>G	p.Pro1973Arg	0.187	375	70	T	B	TPS
MCL10*	*CARD11*	c.1010G>A	p.Arg337Gln	0.170	406	69	T	D	TPS
MCL10*	*IKBKB*	c.1136G>T	p.Gly379Val	0.409	381	156	T	P	TPS
MCL10*	*NIN*	c.1260-8C>T		0.471	329	155			TPS
MCL10*	*CDH8*	c.1247G>A	p.Arg416His	0.462	695	321	T	B	TPS
MCL10*	*CACNA2D3*	c.2318C>T	p.Ala773Val	0.452	828	374	D	D	TPS
MCL10*	*CRYBG3*	c.899C>A	p.Pro300Gln	0.510	1020	520		B	TPS
MCL10*	*ANKRD17*	c.3931A>G	p.Ile1311Val	0.457	488	223	T	B	TPS
MCL11*	*TP53*	c.733G>A	p.Gly245Ser	0.408	152	62	D	D	TPS
MCL11*	*CCND1*	c.32A>G	p.Glu11Gly	0.536	97	52	T	B	TPS
MCL11*	*CCND1*	c.34A>G	p.Thr12Ala	0.505	105	53	T	B	TPS
MCL11*	*CCND1*	c.127T>C	p.Ser43Pro	0.633	316	200	T	B	TPS
MCL11*	*BIRC3*	c.653A>G	p.Lys218Arg	0.600	85	51	T	P	TPS
MCL11*	*CHSY3*	c.1087-9_1087-7delACT		0.481	54	26			TPS
MCL11*	*ATP10B*	c.1793C>T	p.Thr598Ile	0.597	62	37	D	P	TPS
MCL11*	*RGS4*	c.361G>A	p.Gly121Ser	0.500	180	90	T	D	TPS
MCL11*	*SOCS1*	c.227C>G	p.Ala76Gly	0.434	53	23	T	P	TPS
MCL11*	*SOCS1*	c.149C>T	p.Pro50Leu	0.420	50	21	T	B	TPS

MCL11*	*CDH11*	c.932A>G	p.Asp311Gly	0.412	148	61	T	D	TPS
MCL11*	*SUZ12*	c.275-9A>G		0.360	114	41			TPS
MCL11*	*MAP1B*	c.4069C>T	p.Pro1357Ser	0.543	105	57	T	B	TPS
MCL11*	*PCLO*	c.1124_1125delinsCTCTTGGTCCTGCTACCTCCCTCCAC	p.Gln375delinsProLeuGlyProAlaLysProProAlaGlnHis	0.188	154	29			WES
MCL11*	*ATP10B*	c.1793C>T	p.Thr598Ile	0.381	42	16	D	P	WES
MCL11*	*BIRC3*	c.653A>G	p.Lys218Arg	0.638	47	30	T	P	WES
MCL11*	*CCND1*	c.32_34delinsGAG	p.GluThr11GlyAla	0.558	197	110			WES
MCL11*	*CCND1*	c.127T>C	p.Ser43Pro	0.628	430	270	T	B	WES
MCL11*	*DLC1*	c.762_763delinsCG	p.GlnAsn254HisAsp	0.983	60	59			WES
MCL11*	*IKBKB*	c.1600C>T	p.Arg534Trp	0.452	248	112	D	D	WES
MCL11*	*MAP1B*	c.5512T>C	p.Phe1838Leu	0.419	74	31	D	B	WES
MCL11*	*MAP1B*	c.4069C>T	p.Pro1357Ser	0.396	48	19	T	B	WES
MCL11*	*MPDZ*	c.1285A>G	p.Ile429Val	0.603	136	82	T	B	WES
MCL11*	*RGS4*	c.361G>A	p.Gly121Ser	0.463	54	25	T	D	WES
MCL11*	*ROBO2*	c.73_75delinsATC	p.Val25Ile	0.291	203	59			WES
MCL12*	*ATM*	c.4320_4323delAATA	p.Lys1440AsnfsTer10	0.152	92	14			TPS
MCL12*	*PLCG2*	c.2534T>A	p.Leu845Ter	0.523	111	58			TPS
MCL12*	*FAT1*	c.4358G>A	p.Arg1453His	0.534	148	79	D	D	TPS
MCL12*	*CIITA*	c.3063-12G>T		0.476	82	39			TPS
MCL12*	*ATR*	c.325C>T	p.Arg109Trp	0.335	272	91	D	D	TPS
MCL12*	*UBR5*	c.8304C>G	p.Cys2768Trp	0.290	210	61	D	D	TPS
MCL12*	*SLC3A2*	c.157G>A	p.Ala53Thr	0.490	147	72	T	P	TPS
MCL12*	*BIRC3*	c.1325-3A>G		0.277	112	31			TPS

MCL12*	*ARID5B*	c.3128A>G	p.Lys1043Arg	0.451	142	64	D	D	TPS
MCL12*	*YLPM1*	c.5074C>T	p.Arg1692Cys	0.492	244	120	D	D	TPS
MCL12*	*ABCA6*	c.1678G>T	p.Val560Phe	0.441	247	109	T	B	TPS
MCL12*	*LRP1B*	c.2281A>G	p.Ile761Val	0.509	163	83	T	B	TPS
MCL12*	*ANK2*	c.10067G>A	p.Arg3356Lys	0.114	35	4	T	B	WES
MCL12*	*ANK2*	c.10030_10031delinsAA	p.Pro3344Lys	0.102	49	5			WES
MCL12*	*ANK2*	c.4454C>T	p.Ser1485Phe	0.114	35	4	D	D	WES
MCL12*	*ANKRD17*	c.2218G>A	p.Asp740Asn	0.122	41	5	D	D	WES
MCL12*	*ARID1A*	c.2249G>A	p.Arg750Gln	0.167	24	4	D	D	WES
MCL12*	*ARID5B*	c.2399A>G	p.Lys800Arg	0.291	55	16	D	D	WES
MCL12*	*ATM*	c.667G>A	p.Glu223Lys	0.143	28	4	D	D	WES
MCL12*	*ATM*	c.1537C>T	p.Gln513Ter	0.105	38	4			WES
MCL12*	*ATM*	c.2369G>A	p.Cys790Tyr	0.143	28	4	T	B	WES
MCL12*	*ATM*	c.7183G>A	p.Asp2395Asn	0.128	47	6	D	D	WES
MCL12*	*ATM*	c.3467C>T	p.Thr1156Met	0.129	31	4	T	B	WES
MCL12*	*BCL10*	c.46G>A	p.Val16Met	0.222	18	4	D	D	WES
MCL12*	*BCL6*	c.285_327del	p.Asn96CysfsTer40	0.200	20	4			WES
MCL12*	*BIRC3*	c.1409C>T	p.Thr470Ile	0.118	34	4	T	B	WES
MCL12*	*BRCA2*	c.1208T>A	p.Leu403Gln	0.211	19	4	D	D	WES
MCL12*	*BRCA2*	c.6085G>A	p.Glu2029Lys	0.148	27	4	T	B	WES
MCL12*	*CCDC15*	c.919C>T	p.Pro307Ser	0.125	32	4	D	P	WES
MCL12*	*CCND3*	c.469-60_478dup	p.Arg160HisfsTer106	0.146	41	6			WES
MCL12*	*CDK4*	c.581C>T	p.Pro194Leu	0.106	47	5	D	B	WES
MCL12*	*CHSY3*	c.1393G>A	p.Glu465Lys	0.111	36	4	T	P	WES

MCL12*	*CRYBG3*	c.5879G>A	p.Arg1960Lys	0.200	20	4		B	WES
MCL12*	*DGKB*	c.91G>A	p.Asp31Asn	0.160	25	4	D	B	WES
MCL12*	*DLC1*	c.762_763delinsCG	p.GlnAsn254HisAsp	0.609	46	28			WES
MCL12*	*DNAJC6*	c.2765_2766del	p.Val922GlufsTer15	0.150	40	6			WES
MCL12*	*EZH2*	c.1995del	p.Leu666CysfsTer4	0.108	37	4			WES
MCL12*	*FAT1*	c.4358G>A	p.Arg1453His	0.483	29	14	D	D	WES
MCL12*	*FAT1*	c.7922G>A	p.Ser2641Asn	0.267	15	4	T	B	WES
MCL12*	*FAT4*	c.1958C>T	p.Ala653Val	0.118	34	4	T	P	WES
MCL12*	*FAT4*	c.10654_10704del	p.Gly3552_Thr3568del	0.122	41	5			WES
MCL12*	*FAT4*	c.673_786dup	p.Lys225_Asp262dup	0.105	38	4			WES
MCL12*	*FBXO25*	c.91A>C	p.Lys31Gln	0.275	51	14	T	B	WES
MCL12*	*KMT2C*	c.13627_13628del	p.Glu4543ThrfsTer9	0.109	55	6			WES
MCL12*	*KMT2D*	c.10102G>A	p.Val3368Ile	0.138	29	4	T	B	WES
MCL12*	*KMT2D*	c.12039_12117delinsGTCCATGACCCAGAACCTTCT	p.Ala4014SerfsTer17	0.154	26	4			WES
MCL12*	*KMT2D*	c.16387G>A	p.Ala5463Thr	0.133	30	4	D	D	WES
MCL12*	*KMT2D*	c.1087G>A	p.Glu363Lys	0.111	36	4	T	B	WES
MCL12*	*KMT2D*	c.12296G>A	p.Gly4099Glu	0.250	16	4	D	D	WES
MCL12*	*KMT2D*	c.10154T>C	p.Met3385Thr	0.133	30	4	D	B	WES
MCL12*	*KMT2D*	c.5792_5829del	p.Pro1931LeufsTer13	0.111	36	4			WES
MCL12*	*KMT2D*	c.7142C>T	p.Pro2381Leu	0.182	22	4	T	D	WES
MCL12*	*KMT2D*	c.9677C>T	p.Pro3226Leu	0.103	39	4	T	B	WES
MCL12*	*LRP1B*	c.12391G>A	p.Ala4131Thr	0.108	37	4	T	B	WES
MCL12*	*LRP1B*	c.2281A>G	p.Ile761Val	0.273	66	18	T	B	WES
MCL12*	*MKI67*	c.4547_4550delinsAGCT	p.ArgPro1516GlnLeu	0.426	101	43			WES

MCL12*	*MKI67*	c.2260C>T	p.Pro754Ser	0.121	33	4	T	B	WES
MCL12*	*MTOR*	c.3679G>A	p.Glu1227Lys	0.129	31	4	T	P	WES
MCL12*	*NCOR2*	c.2762C>T	p.Ser921Phe	0.133	30	4	D	D	WES
MCL12*	*NCOR2*	c.6628G>A	p.Glu2210Lys	0.105	38	4	D	D	WES
MCL12*	*NCOR2*	c.194G>A	p.Arg65Lys	0.133	60	8	D	P	WES
MCL12*	*NOTCH2*	c.2491C>T	p.Gln831Ter	0.118	34	4			WES
MCL12*	*OGDHL*	c.1220C>T	p.Ala407Val	0.102	59	6	D	P	WES
MCL12*	*PCLO*	c.5116G>A	p.Asp1706Asn	0.105	38	4	T	D	WES
MCL12*	*PCLO*	c.910_989del	p.Pro304AlafsTer40	0.119	42	5			WES
MCL12*	*PDLIM3*	c.29C>T	p.Pro10Leu	0.344	32	11	D	D	WES
MCL12*	*RB1*	c.536G>A	p.Ser179Asn	0.108	37	4	T	B	WES
MCL12*	*RELN*	c.4231C>T	p.Pro1411Ser	0.148	27	4	T	D	WES
MCL12*	*RIMS2*	c.196G>A	p.Glu66Lys	0.143	28	4	D	P	WES
MCL12*	*RIMS2*	c.1422G>A	p.Met474Ile	0.121	58	7	D	P	WES
MCL12*	*ROBO2*	c.73_75delinsATC	p.Val25Ile	0.146	130	19			WES
MCL12*	*SETD2*	c.1276C>T	p.His426Tyr	0.138	29	4	D	P	WES
MCL12*	*SETD2*	c.3790C>T	p.Gln1264Ter	0.121	33	4			WES
MCL12*	*SF3B1*	c.2431C>T	p.Leu811Phe	0.109	55	6	D	D	WES
MCL12*	*SGK1*	c.136_138delinsT	p.Thr46TrpfsTer25	0.121	33	4			WES
MCL12*	*SI*	c.691_692inv	p.Thr231Val	0.105	38	4			WES
MCL12*	*SPEN*	c.374G>A	p.Arg125Gln	0.147	34	5	D	D	WES
MCL12*	*SYNE1*	c.13147G>A	p.Glu4383Lys	0.129	31	4	T	D	WES
MCL12*	*SYNE1*	c.18751G>A	p.Glu6251Lys	0.154	39	6	T	B	WES
MCL12*	*SYNE1*	c.772G>A	p.Gly258Arg	0.143	28	4	D	P	WES

MCL12*	*SYNE1*	c.16199_16228del	p.Gly5400_Asp5409del	0.109	55	6			WES
MCL12*	*TUBA1B*	c.859T>A	p.Ser287Thr	0.182	22	4		B	WES
MCL12*	*UBR5*	c.8304C>G	p.Cys2768Trp	0.228	92	21	D	D	WES
MCL12*	*UBR5*	c.1051C>A	p.Gln351Lys	0.114	35	4	T	B	WES
MCL12*	*UBR5*	c.2327G>A	p.Gly776Glu	0.235	17	4	D	D	WES
MCL13†	*SP140*	c.646delC	p.Leu216TyrfsTer15	0.421	468	197			TPS
MCL13†	*TP53*	c.329G>C	p.Arg110Pro	0.801	146	117	D	D	TPS
MCL13†	*TP53*	XR_243565.1:n.468G>C		0.801	146	117	D	D	TPS
MCL13†	*NOTCH2*	c.5621A>G	p.Asp1874Gly	0.440	175	77	D	D	TPS
MCL13†	*DYNC1H1*	c.6529G>A	p.Ala2177Thr	0.923	207	191	T	B	TPS
MCL13†	*FLYWCH1*	c.1775A>T	p.Asp592Val	0.390	77	30	D	B	TPS
MCL13†	*NSD1*	c.487G>T	p.Asp163Tyr	0.520	743	386	D	P	TPS
MCL13†	*KMT2A*	c.218G>A	p.Gly73Glu	0.455	143	65	D	B	TPS
MCL13†	*S1PR2*	c.17C>T	p.Ser6Leu	0.428	159	68		P	TPS
MCL13†	*ANKRD44*	c.1766C>T	p.Ser589Leu	0.551	363	200	T	P	TPS
MCL13†	*CASP10*	c.814-10A>G		0.475	217	103			TPS
MCL13†	*CRYBG3*	c.7542C>G	p.His2514Gln	0.533	548	292		B	TPS
MCL13†	*PLXNA1*	c.5627C>T	p.Ala1876Val	0.436	335	146	D	P	TPS
MCL13†	*DGKB*	c.1744A>C	p.Ile582Leu	0.311	837	260	D	D	TPS
MCL13†	*PCLO*	c.8509A>G	p.Arg2837Gly	0.445	292	130	D	P	TPS
MCL13†	*PCLO*	c.5891C>T	p.Thr1964Met	0.432	463	200	D	P	TPS
MCL14†	*PCLO*	c.1123_1124insCT	p.Gln375ProfsTer18	0.121	33	4			TPS
MCL14†	*PCLO*	c.1124_1126delAGA	p.Gln375_Thr376delinsPro	0.111	36	4			TPS
MCL14†	*NF1*	c.3859T>C	p.Phe1287Leu	0.218	500	109	D	D	TPS

MCL14†	*CCND1*	c.39C>G	p.Ile13Met	0.818	538	440	T	B	TPS
MCL14†	*FAT1*	c.2114A>C	p.Asp705Ala	0.447	226	101	D	D	TPS
MCL14†	*MEF2C*	c.349T>C	p.Tyr117His	0.330	349	115	D	D	TPS
MCL14†	*ABCA7*	c.2540A>G	p.Lys847Arg	0.440	116	51	D	D	TPS
MCL14†	*RGS4*	c.706C>T	p.Arg236Trp	0.461	206	95	D	P	TPS
MCL14†	*NAV3*	c.4586G>A	p.Arg1529His	0.469	209	98	D	D	TPS
MCL14†	*TRAF3*	c.139G>A	p.Val47Met	0.329	456	150	T	D	TPS
MCL14†	*MAP2*	c.4585-1138A>G		0.614	197	121			TPS
MCL14†	*ANK2*	c.9682G>A	p.Val3228Met	0.531	143	76	T	B	TPS
MCL14†	*CD83*	c.220C>T	p.Arg74Cys	0.518	363	188	D	B	TPS
MCL14†	*CARD11*	c.2801C>T	p.Ser934Leu	0.554	83	46	D	B	TPS
MCL15†	*CARD11*	c.692_694dupAGC	p.Gln231dup	0.183	82	15			TPS
MCL15†	*LRP1B*	c.268T>A	p.Cys90Ser	0.286	154	44	D	D	TPS
MCL15†	*ABCA6*	c.940T>G	p.Leu314Val	0.550	80	44	T	B	TPS
MCL15†	*TCF3*	c.1162G>A	p.Gly388Ser	0.418	677	283	T	B	TPS
MCL15†	*CRYBG3*	c.4303C>T	p.Arg1435Trp	0.615	104	64			TPS
MCL15†	*ANKRD17*	c.4918G>T	p.Ala1640Ser	0.386	176	68	D	B	TPS
MCL15†	*SI*	c.749G>A	p.Arg250His	0.552	143	79	D	D	TPS
MCL15†	*SPEN*	c.8386C>G	p.Arg2796Gly	0.462	1079	499	D	P	TPS
MCL15†	*COL16A1*	c.739-3C>T		0.580	69	40			TPS
MCL15†	*DTX1*	c.1792G>A	p.Ala598Thr	0.292	665	194	D	B	TPS
MCL15†	*ABCA3*	c.3613G>A	p.Gly1205Arg	0.322	146	47	T	B	TPS
MCL15†	*ABCA7*	c.1001G>A	p.Arg334Gln	0.417	307	128	T	B	TPS
MCL15†	*SETD2*	c.5306C>A	p.Ser1769Tyr	0.610	195	119	T	P	TPS

MCL15†	*EPHA3*	c.407G>A	p.Arg136Gln	0.394	132	52	T	B	TPS
MCL15†	*PLXNA1*	c.5627C>T	p.Ala1876Val	0.351	1315	461	D	P	TPS
MCL15†	*ABCA13*	c.9200A>G	p.Asp3067Gly	0.477	44	21	D	B	TPS
MCL15†	*PLXNB3*	c.3037G>A	p.Ala1013Thr	1.000	281	281	T	B	TPS
MCL16†	*ABCC9*	c.3589C>T	p.Arg1197Cys	0.513	78	40	D	D	TPS
MCL16†	*KMT2D*	c.7036G>A	p.Gly2346Ser	0.571	35	20	T	P	TPS
MCL16†	*MEF2C*	c.259-99C>T		0.538	93	50			TPS
MCL16†	*CDH23*	c.9047G>A	p.Arg3016His	0.538	52	28	T	P	TPS
MCL16†	*ABCA7*	c.67-3delC		0.485	33	16			TPS
MCL16†	*MUC16*	c.2609C>T	p.Ser870Leu	0.458	59	27	T	B	TPS
MCL16†	*ABCA13*	c.12070+5G>A		0.167	24	4			TPS
MCL16†	*ATP11C*	c.3329C>T	p.Ser1110Phe	0.250	24	6	D	B	TPS
MCL16†	*ATRX*	c.3086C>T	p.Pro1029Leu	1.000	29	29	D	B	TPS
MCL16†	*FAT1*	c.11251A>G	p.Lys3751Glu	0.470	66	31	T	B	TPS
MCL16†	*ATP10B*	c.3971G>A	p.Gly1324Glu	0.476	63	30	T	P	TPS
MCL16†	*PCLO*	c.9015T>G	p.Phe3005Leu	0.277	65	18	T	B	TPS
MCL16†	*PKHD1L1*	c.6536C>T	p.Ala2179Val	0.478	69	33	T	B	TPS
MCL16†	*JAK2*	c.227-7T>C		0.512	41	21			TPS
MCL17†	*TP53*	c.994-2A>G		0.484	62	30			TPS
MCL17†	*ASXL1*	c.2694G>A	p.Trp898Ter	0.188	96	18			TPS
MCL17†	*BRCC3*	c.241delC	p.Arg81AspfsTer10	0.215	65	14			TPS
MCL17†	*PKHD1L1*	c.11354C>A	p.Pro3785Gln	0.317	123	39	D	D	TPS
MCL17†	*SLC3A2*	c.113-806A>G		0.191	47	9	T	B	TPS
MCL17†	*MAP1B*	c.6110C>T	p.Thr2037Ile	0.469	177	83	D	B	TPS

MCL17†	*ANK2*	c.11320G>A	p.Val3774Met	0.488	43	21	D	D	TPS
MCL17†	*PKHD1L1*	c.1933G>A	p.Ala645Thr	0.461	180	83	T	B	TPS
MCL18	*ATM*	c.5251G>T	p.Glu1751Ter	0.351	74	26			TPS
MCL18	*ZRSR2*	c.356_359delGAGA	p.Arg119LysfsTer45	0.585	41	24			TPS
MCL18	*TBL1XR1*	c.497G>A	p.Arg166Gln	0.206	107	22	T	B	TPS
MCL18	*NOTCH2*	c.6982T>C	p.Cys2328Arg	0.500	174	87	T	B	TPS
MCL18	*KCNC2*	c.1579G>A	p.Gly527Ser	0.473	55	26	T	B	TPS
MCL18	*FAT1*	c.11818A>G	p.Thr3940Ala	0.435	23	10	T	B	TPS
MCL18	*CARD11*	c.3260+3A>G		0.598	107	64			TPS
MCL18	*ACTB*	c.883G>A	p.Ala295Thr	0.108	83	9		B	WES
MCL18	*ANK2*	c.8240G>A	p.Arg2747His	0.385	52	20	D	B	WES
MCL18	*ANK2*	c.6023C>T	p.Thr2008Ile	0.160	25	4	D	B	WES
MCL18	*APC*	c.4485_4555dup	p.Asp1519ValfsTer12	0.182	22	4			WES
MCL18	*ATM*	c.5251G>T	p.Glu1751Ter	0.287	115	33			WES
MCL18	*ATM*	c.7528A>T	p.Lys2510Ter	0.477	44	21			WES
MCL18	*ATRX*	c.6301G>A	p.Glu2101Lys	0.118	34	4	T	D	WES
MCL18	*BRCA2*	c.2668_2700del	p.Phe890_Asn900del	0.125	32	4			WES
MCL18	*CASP8*	c.1289G>A	p.Arg430Gln	0.133	30	4	D	D	WES
MCL18	*CCDC15*	c.2296G>A	p.Asp766Asn	0.103	39	4	T	B	WES
MCL18	*CEBPB*	c.51_56del	p.Pro18_Pro19del	0.111	36	4			WES
MCL18	*CHEK1*	c.448_512del	p.Gly150ThrfsTer19	0.143	42	6			WES
MCL18	*COL16A1*	c.1939C>T	p.Arg647Cys	0.118	34	4	T	D	WES
MCL18	*CREBBP*	c.3644G>A	p.Gly1215Glu	0.103	39	4	D	D	WES
MCL18	*DLC1*	c.762_763delinsCG	p.GlnAsn254HisAsp	0.872	47	41			WES

MCL18	*FAT1*	c.11818A>G	p.Thr3940Ala	0.339	62	21	T	B	WES
MCL18	*GON4L*	c.4504C>T	p.Arg1502Cys	0.122	49	6	D	B	WES
MCL18	*HEPH*	c.716_790del	p.Asn239_Glu263del	0.138	29	4			WES
MCL18	*IGLL5*	c.563A>G	p.Lys188Arg	0.111	36	4	T	B	WES
MCL18	*KCNC2*	c.1579G>A	p.Gly527Ser	0.481	77	37	T	B	WES
MCL18	*KMT2C*	c.6499C>T	p.Arg2167Trp	0.103	39	4	D	D	WES
MCL18	*KMT2D*	c.13885A>C	p.Thr4629Pro	0.316	19	6	D	D	WES
MCL18	*MAP3K14*	c.2281G>A	p.Gly761Arg	0.563	16	9		D	WES
MCL18	*MKI67*	c.5627_5630delinsAGCT	p.ArgPro1876GlnLeu	0.952	63	60			WES
MCL18	*MPDZ*	c.4919G>A	p.Gly1640Asp	0.121	33	4	D	D	WES
MCL18	*NIN*	c.3748_3749ins	p.Lys1249_Leu1250ins	0.139	36	5			WES
MCL18	*NOTCH2*	c.6982T>C	p.Cys2328Arg	0.482	27	13	T	B	WES
MCL18	*NOTCH2*	c.2042T>A	p.Ile681Asn	0.490	51	25	T	B	WES
MCL18	*NOTCH3*	c.4348G>A	p.Ala1450Thr	0.386	83	32	T	D	WES
MCL18	*PCLO*	c.8582C>T	p.Ala2861Val	0.143	28	4	T	B	WES
MCL18	*PCLO*	c.1123_1124ins	p.Gln374_Gln375ins	0.281	32	9			WES
MCL18	*PLXNB3*	c.4259C>T	p.Thr1420Met	0.125	32	4	D	D	WES
MCL18	*RELN*	c.6142G>A	p.Gly2048Arg	0.114	35	4	T	D	WES
MCL18	*RIMS2*	c.162_163ins	p.Gln55LysfsTer64	0.143	28	4			WES
MCL18	*ROBO2*	c.73_75delinsATC	p.Val25Ile	0.234	184	43			WES
MCL18	*RYR1*	c.14039G>A	p.Arg4680Gln	0.148	27	4	D	D	WES
MCL18	*RYR1*	c.3161C>T	p.Pro1054Leu	0.108	37	4	D	B	WES
MCL18	*RYR1*	c.13836G>A	p.Trp4612Ter	0.108	37	4			WES
MCL18	*SETD2*	c.1270C>T	p.Arg424Ter	0.172	29	5			WES

MCL18	*SMC1A*	c.3406G>A	p.Ala1136Thr	0.154	26	4	D	D	WES
MCL18	*SP140*	c.1712G>A	p.Arg571Gln	0.174	23	4	T	P	WES
MCL18	*SPEN*	c.3410G>A	p.Arg1137His	0.118	34	4	T	B	WES
MCL18	*SYNE1*	c.298G>A	p.Glu100Lys	0.154	26	4	T	D	WES
MCL18	*TBC1D26*	c.481C>A	p.Gln161Lys	0.114	35	4	D	B	WES
MCL18	*TBL1XR1*	c.497G>A	p.Arg166Gln	0.224	67	15	T	B	WES
MCL18	*TERT*	c.1589C>T	p.Pro530Leu	0.154	26	4	D	D	WES
MCL18	*TNRC6B*	c.2654G>A	p.Arg885Gln	0.111	36	4	D	D	WES
MCL18	*TRAF3*	c.1283G>A	p.Arg428Gln	0.138	29	4	D	D	WES
MCL18	*UBR5*	c.8275_8345del	p.Asp2759SerfsTer10	0.106	47	5			WES
MCL18	*WHSC1*	c.3211G>A	p.Asp1071Asn	0.125	32	4	T	B	WES
MCL18	*YLPM1*	c.4381_4382del	p.Gln1461ArgfsTer21	0.103	39	4			WES
MCL18	*ZRSR2*	c.356_359del	p.Arg119LysfsTer45	0.750	20	15			WES
MCL19	*KMT2A*	c.838_839insT	p.Pro280LeufsTer5	0.143	42	6			TPS
MCL19	*PDCD1LG2*	c.584G>A	p.Trp195Ter	0.583	96	56			TPS
MCL19	*SYNE1*	c.16339C>T	p.Arg5447Trp	0.484	126	61	D	D	TPS
MCL19	*BTG2*	c.443C>G	p.Pro148Arg	0.479	292	140	D	P	TPS
MCL19	*MUC16*	c.12154A>T	p.Thr4052Ser	0.500	110	55	D	P	TPS
MCL19	*PRDM15*	c.1382A>G	p.Lys461Arg	0.280	50	14	D	D	TPS
MCL19	*KIAA1671*	c.4795G>A	p.Val1599Met	0.307	127	39	D	D	TPS
MCL19	*SI*	c.3875A>G	p.Tyr1292Cys	0.610	82	50	D	D	TPS
MCL19	*ACTB*	c.985-9C>T		0.640	175	112			TPS
MCL19	*COL14A1*	c.4621G>A	p.Val1541Ile	0.615	65	40	T	B	TPS
MCL19	*NOTCH1*	c.3553G>A	p.Asp1185Asn	0.471	444	209	T	B	TPS

MCL20*	*ANK2*	c.10900G>A	p.Val3634Ile	0.509	165	84	T	B	TPS
MCL20*	*SLC3A2*	c.128G>C	p.Cys43Ser	0.389	72	28	T	P	TPS
MCL20*	*ATM*	c.8839A>T	p.Thr2947Ser	0.242	248	60	D	D	TPS
MCL20*	*ARID2*	c.2806G>T	p.Gly936Cys	0.528	233	123	D	D	TPS
MCL20*	*LRIG3*	c.2431G>A	p.Val811Met	0.390	310	121	D	D	TPS
MCL20*	*CXCR4*	c.685T>A	p.Ser229Thr	0.472	2547	1202	D	B	TPS
MCL20*	*MAP1B*	c.2738A>C	p.Glu913Ala	0.471	342	161	D	D	TPS
MCL20*	*ATP10B*	c.1237A>G	p.Ile413Val	0.440	193	85	T	B	TPS
MCL20*	*SYNE1*	c.12616G>A	p.Ala4206Thr	0.538	158	85	D	D	TPS
MCL20*	*SYNE1*	c.4215C>A	p.Asn1405Lys	0.505	204	103	T	P	TPS
MCL21†	*TP53*	c.488A>G	p.Tyr163Cys	0.945	182	172	D	D	TPS
MCL21†	*ZNF429*	c.1978A>T	p.Arg660Ter	0.238	63	15			TPS
MCL21†	*RELN*	c.1984_1985delAT	p.Met662ValfsTer4	0.386	70	27			TPS
MCL21†	*DLGAP2*	c.371G>T	p.Arg124Leu	0.551	287	158	D	D	TPS
MCL21†	*CDKN2A*	c.-33G>C		0.615	78	48			TPS
MCL21†	*NOTCH2*	c.3943A>T	p.Asn1315Tyr	0.462	130	60	D	D	TPS
MCL21†	*KMT2D*	c.11331G>A	p.Met3777Ile	0.310	29	9	D	B	TPS
MCL21†	*SH2B3*	c.1703T>C	p.Ile568Thr	0.390	77	30	D	D	TPS
MCL21†	*TCF3*	c.1948C>A	p.Pro650Thr	0.473	91	43	T	D	TPS
MCL21†	*CACNA2D3*	c.1066G>A	p.Gly356Arg	1.000	40	40	T	B	TPS
MCL21†	*MAP1B*	c.3001G>A	p.Glu1001Lys	0.328	125	41	D	D	TPS
MCL21†	*KMT2C*	c.11074G>A	p.Ala3692Thr	0.423	130	55	T	B	TPS
MCL22†	*ETV6*	c.33+1G>A		0.374	195	73			TPS
MCL22†	*FAT1*	c.1delA	p.Met1?	0.351	37	13			TPS

MCL22†	*TMSB4X*	c.97G>T	p.Glu33Ter	0.518	139	72			TPS
MCL22†	*PDLIM3*	c.317A>C	p.Glu106Ala	0.473	74	35	T	D	TPS
MCL22†	*ATRX*	c.1964G>A	p.Arg655Lys	0.203	79	16	D	D	TPS
MCL22†	*MEF2B*	c.248A>C	p.Asp83Ala	0.424	403	171	D	D	TPS
MCL22†	*DSEL*	c.887C>A	p.Ser296Tyr	0.219	96	21	T	D	TPS
MCL22†	*IGLL5*	c.161C>T	p.Ala54Val	0.462	481	222	D	B	TPS
MCL22†	*SYK*	c.1054T>C	p.Tyr352His	0.693	150	104	D	D	TPS
MCL22†	*ABCA6*	c.2195C>A	p.Pro732His	0.514	105	54	D	D	TPS
MCL22†	*PHLPP1*	c.1864A>G	p.Thr622Ala	0.300	90	27	T	D	TPS
MCL22†	*DNMT3B*	c.1603G>A	p.Val535Ile	0.443	567	251	T	B	TPS
MCL22†	*CRYBG3*	c.568T>A	p.Ser190Thr	0.607	135	82		D	TPS
MCL22†	*FAT1*	c.12670G>A	p.Ala4224Thr	0.487	76	37	T	B	TPS
MCL22†	*FAT1*	c.367C>T	p.Leu123Phe	0.611	72	44	D	B	TPS
MCL22†	*SYNE1*	c.13903G>C	p.Val4635Leu	0.508	63	32	T	B	TPS
MCL22†	*SYNE1*	c.10060G>A	p.Val3354Ile	0.685	73	50	D	B	TPS
MCL22†	*EZH2*	c.1546+7A>G		0.855	69	59			TPS
MCL23*	*FAS*	c.3G>A	p.Met1?	0.327	615	201	D	B	TPS
MCL23*	*FBXW7*	c.1418+1G>A		0.367	463	170			TPS
MCL23*	*PIM1*	c.379C>T	p.Gln127Ter	0.286	971	278			TPS
MCL23*	*CCND3*	c.811dupC	p.Arg271ProfsTer53	0.248	750	186			TPS
MCL23*	*PRDM1*	c.291+1G>A		0.515	262	135			TPS
MCL23*	*ABCA7*	c.3148-5C>G		0.528	388	205			TPS
MCL23*	*FLG*	c.2431G>A	p.Gly811Arg	0.256	618	158	D	D	TPS
MCL23*	*SLC16A7*	c.1308G>T	p.Arg436Ser	0.327	627	205	T	B	TPS

MCL23*	*B2M*	c.67+4A>T		0.511	309	158			TPS
MCL23*	*IGLL5*	c.26G>C	p.Gly9Ala	0.340	1117	380	T	B	TPS
MCL23*	*ROBO2*	c.3064A>C	p.Asn1022His	0.235	616	145	T	D	TPS
MCL23*	*IRF4*	c.-2G>T		0.338	999	338			TPS
MCL23*	*PIM1*	c.72G>C	p.Lys24Asn	0.350	635	222	T	B	TPS
MCL23*	*PRDM1*	c.62C>T	p.Ser21Phe	0.540	352	190	D	B	TPS
MCL23*	*CARD11*	c.1159G>T	p.Asp387Tyr	0.634	388	246	D	D	TPS
MCL23*	*KMT2C*	c.8069A>T	p.Asp2690Val	0.409	470	192	D	D	TPS
MCL23*	*MYC*	c.475C>T	p.Leu159Phe	0.289	761	220	D	D	TPS
MCL23*	*FIGN*	c.1531G>A	p.Ala511Thr	0.442	231	102	T	D	TPS
MCL23*	*IGLL5*	c.173G>A	p.Ser58Asn	0.349	459	160	D	B	TPS
MCL23*	*IGLL5*	c.182C>T	p.Ser61Phe	0.399	439	175	T	B	TPS
MCL23*	*OR5H1*	c.454G>C	p.Gly152Arg	0.184	515	95	D	D	TPS
MCL23*	*SI*	c.4999G>A	p.Val1667Ile	0.337	552	186	T	B	TPS
MCL23*	*ANK2*	c.6073G>A	p.Gly2025Ser	0.515	571	294	T	B	TPS
MCL23*	*ANK2*	c.10019A>G	p.Asp3340Gly	0.437	389	170	T	B	TPS
MCL23*	*PDLIM3*	c.14T>C	p.Val5Ala	0.475	448	213	D	D	TPS
MCL23*	*MAP1B*	c.4198A>G	p.Ser1400Gly	0.526	310	163	D	P	TPS
MCL23*	*PCLO*	c.3539T>C	p.Leu1180Pro	0.452	651	294	T	B	TPS
MCL24†	*ATM*	c.2296A>T	p.Lys766Ter	0.583	24	14			TPS
MCL24†	*NFKBIE*	c.762C>A	p.Tyr254Ter	0.386	874	337			TPS
MCL24†	*SENP6*	c.1604dupG	p.Val536SerfsTer3	0.121	33	4			TPS
MCL24†	*FLG*	c.5368C>T	p.Gln1790Ter	0.495	764	378			TPS
MCL24†	*ZNF429*	c.778A>G	p.Lys260Glu	0.172	29	5	T	B	TPS

MCL24†	*CRYBG3*	c.1211C>T	p.Thr404Ile	0.535	86	46		B	TPS
MCL24†	*PKHD1L1*	c.12083C>G	p.Ala4028Gly	0.548	135	74	D	D	TPS
MCL24†	*YLPM1*	c.3004C>T	p.Arg1002Trp	0.577	71	41		B	TPS
MCL24†	*ANKRD44*	c.1766C>T	p.Ser589Leu	0.392	278	109	T	P	TPS
MCL24†	*COL6A3*	c.6592-3C>T		0.465	256	119			TPS
MCL24†	*ESX1*	c.1013_1039dupGTGTGCCACCCGGGCCGCCCATGGCGC	p.Arg338_Ala346dup	0.888	250	222			TPS
MCL25†	*CD79A*	c.546_548delTGA	p.Tyr182_Glu183delinsTer	0.282	71	20			TPS
MCL25†	*SP140*	c.2112T>A	p.Cys704Ter	0.421	423	178			TPS
MCL25†	*EWSR1*	c.1834C>T	p.Arg612Ter	0.736	802	590			TPS
MCL25†	*KCNH6*	c.2213_2216delTTCT	p.Phe738SerfsTer48	0.365	211	77			TPS
MCL25†	*NFKBIE*	c.139delC	p.Arg47AlafsTer2	0.473	514	243			TPS
MCL25†	*COL11A2*	c.539G>A	p.Arg180His	0.413	143	59	T	B	TPS
MCL25†	*BLM*	c.1044G>A	p.Met348Ile	0.427	117	50	D	B	TPS
MCL25†	*FAT4*	c.13285G>T	p.Asp4429Tyr	0.379	850	322	D	P	TPS
MCL25†	*MAP3K14*	c.1868C>T	p.Pro623Leu	0.502	1309	657			TPS
MCL25†	*U2AF2*	c.691G>A	p.Asp231Asn	0.371	224	83	D	D	TPS
MCL25†	*MAP2*	c.4651C>T	p.Arg1551Trp	0.450	209	94	D	D	TPS
MCL25†	*KMT2C*	c.2698G>C	p.Gly900Arg	0.383	120	46	D	D	TPS
MCL25†	*TYK2*	c.2098C>T	p.Arg700Trp	0.486	1250	608	D	D	TPS
MCL25†	*PKHD1L1*	c.11590C>T	p.Arg3864Cys	0.534	73	39	D	D	TPS
MCL25†	*ARID1A*	c.6707G>A	p.Arg2236His	0.369	1459	538	D	D	TPS
MCL25†	*CDH23*	c.3352G>A	p.Gly1118Ser	0.532	725	386		B	TPS
MCL25†	*MMP8*	c.378G>C	p.Glu126Asp	0.458	166	76	D	B	TPS

MCL25†	*ETV6*	c.572G>T	p.Arg191Leu	0.459	628	288	D	B	TPS
MCL25†	*SRRM2*	c.5834G>A	p.Arg1945His	0.457	819	374		D	TPS
MCL25†	*ABCA6*	c.1830C>A	p.Asn610Lys	0.571	42	24	T	B	TPS
MCL25†	*TCF3*	c.1948C>A	p.Pro650Thr	0.487	1593	775	T	D	TPS
MCL25†	*MAP1B*	c.3166G>A	p.Gly1056Ser	0.460	315	145	T	B	TPS
MCL25†	*HEPH*	c.1490+6G>C		0.805	77	62			TPS
MCL25†	*TP53*	c.524G>A	p.Arg175His	0.867	1470	1274	D	D	TPS
MCL25†	*BIRC3*	c.1325-1G>A		0.333	12	4			TPS
MCL25†	*ZNF608*	c.3956delC	p.Pro1319LeufsTer2	0.390	172	67			TPS
MCL25†	*CDH23*	c.4663C>T	p.Arg1555Cys	0.457	854	390		D	TPS
MCL25†	*ATM*	c.5063T>C	p.Ile1688Thr	0.836	61	51	D	B	TPS
MCL25†	*PRDM15*	c.3533G>A	p.Ser1178Asn	0.380	440	167	T	D	TPS
MCL25†	*PCDHGA12*	c.275G>A	p.Arg92Gln	0.517	786	406	D	D	TPS
MCL25†	*CARD11*	c.1079T>C	p.Met360Thr	0.491	613	301	D	B	TPS
MCL25†	*POT1*	c.259C>G	p.Gln87Glu	0.148	81	12	T	P	TPS
MCL25†	*KMT2D*	c.14581G>A	p.Asp4861Asn	0.392	334	131	D	D	TPS
MCL25†	*ZFHX3*	c.197C>T	p.Ala66Val	0.429	730	313	T	B	TPS
MCL25†	*WHSC1*	c.3295G>A	p.Glu1099Lys	0.461	380	175	D	D	TPS
MCL25†	*RELN*	c.8212C>T	p.Arg2738Trp	0.488	203	99	D	D	TPS
MCL26†	*ATM*	c.4176dupT	p.Ile1393TyrfsTer6	0.733	30	22			TPS
MCL26†	*HOXB2*	c.569G>A	p.Trp190Ter	0.425	1077	458			TPS
MCL26†	*PRDM1*	c.351delA	p.Ile117MetfsTer46	0.493	71	35			TPS
MCL26†	*BRCA2*	c.572A>T	p.Asp191Val	0.348	69	24	D	D	TPS

MCL26†	*ESX1*	c.1013_1039dupGTGTGCCACCCGGGCCGCCCATGGCGC	p.Arg338_Ala346dup	0.709	117	83			TPS
MCL26†	*ATRX*	c.6026C>T	p.Ala2009Val	0.182	22	4	T	P	TPS
MCL26†	*SMARCA4*	c.3713C>T	p.Ser1238Phe	0.363	2244	815	D	D	TPS
MCL26†	*CRYBG3*	c.7394C>T	p.Pro2465Leu	0.548	31	17		P	TPS
MCL26†	*TRAF2*	c.797C>T	p.Ser266Leu	0.480	1512	726	T	B	TPS
MCL26†	*ZFHX3*	c.10445G>T	p.Ser3482Ile	0.461	1021	471	D	B	TPS
MCL26†	*ABCA7*	c.805G>A	p.Glu269Lys	0.485	981	476	T	B	TPS
MCL26†	*IKBKB*	c.1031C>T	p.Thr344Met	0.412	798	329	D	D	TPS
MCL26†	*ABL1*	c.1678G>A	p.Asp560Asn	0.431	520	224	T	B	TPS
MCL26†	*PCLO*	c.1124_1125delinsCTCTTGGTCCTGCTACCTCCCTCCAC	p.Gln375delinsProLeu	0.179	106	19			WES
MCL26†	*ABCA7*	c.805G>A	p.Glu269Lys	0.501	515	258	T	B	WES
MCL26†	*ABL1*	c.1735G>A	p.Asp579Asn	0.370	81	30	T	B	WES
MCL26†	*ATM*	c.4176dup	p.Ile1393TyrfsTer6	0.500	14	7			WES
MCL26†	*BRCA2*	c.572A>T	p.Asp191Val	0.604	53	32	D	D	WES
MCL26†	*CRYBG3*	c.7394C>T	p.Pro2465Leu	0.500	22	11		P	WES
MCL26†	*DLC1*	c.762_763delinsCG	p.GlnAsn254HisAsp	0.964	28	27			WES
MCL26†	*HOXB2*	c.569G>A | .	p.Trp190Ter	0.388	338	131			WES
MCL26†	*IKBKB*	c.1031C>T	p.Thr344Met	0.403	72	29	D	D	WES
MCL26†	*KMT2C*	c.1181G>A	p.Cys394Tyr	0.113	124	14	D	D	WES
MCL26†	*LRP1B*	c.9191G>A	p.Arg3064Gln	0.133	30	4	T	D	WES
MCL26†	*MAP3K14*	c.763C>T	p.Arg255Cys	0.505	101	51		B	WES
MCL26†	*PCLO*	c.8780_8781ins	p.Asp2926_Glu2927ins	0.351	37	13			WES

MCL26†	*PRDM1*	c.351del	p.Ile117MetfsTer46	0.394	33	13			WES
MCL26†	*RIMS2*	c.476C>A	p.Pro159Gln	0.167	24	4	D	P	WES
MCL26†	*ROBO2*	c.73_75delinsATC	p.Val25Ile	0.129	85	11			WES
MCL26†	*S1PR1*	c.851_862del	p.Val284_Cys287del	0.313	96	30			WES
MCL26†	*SMARCA4*	c.3713C>T	p.Ser1238Phe	0.353	383	135	D	D	WES
MCL26†	*SYNE1*	c.12149_12150delinsGT	p.Lys4050Ser	0.546	22	12			WES
MCL26†	*TCF3*	c.1291_1293delinsAGT	p.Gly431Ser	0.427	150	64			WES
MCL26†	*TRAF2*	c.797C>T	p.Ser266Leu	0.468	284	133	T	B	WES
MCL26†	*UBR5*	c.619C>T	p.Arg207Ter	0.167	24	4			WES
MCL26†	*YLPM1*	c.961G>A	p.Val321Met	0.108	37	4	D	B	WES
MCL26†	*ZFHX3*	c.10445G>T	p.Ser3482Ile	0.446	413	184	D	B	WES
MCL27	*CHEK2*	c.538C>T	p.Arg180Cys	0.458	48	22	D	P	TPS
MCL27	*CDH23*	c.8401T>G	p.Phe2801Val	0.525	566	297	T	B	TPS
MCL27	*ESX1*	c.1013_1039dupGTGTGCCACCCGGGCCGCCCATGGCGC	p.Arg338_Ala346dup	0.750	52	39			TPS
MCL27	*ATM*	c.7280T>G	p.Leu2427Arg	0.548	31	17	D	D	TPS
MCL27	*CREBBP*	c.3060+5G>A		0.258	256	66			TPS
MCL27	*SMARCA4*	c.1679A>G	p.Tyr560Cys	0.359	1114	400	D	D	TPS
MCL27	*ZNF90*	c.1601C>A	p.Ala534Glu	0.360	25	9	T	B	TPS
MCL27	*LRP1B*	c.12515-7T>A		0.167	12	2			TPS
MCL27	*WHSC1*	c.3448A>G	p.Thr1150Ala	0.310	387	120	D	D	TPS
MCL27	*PCLO*	c.7220C>T	p.Pro2407Leu	0.409	137	56	T	B	TPS
MCL27	*COL4A2*	c.5033C>T	p.Thr1678Ile	0.409	1337	547	D	D	TPS
MCL27	*MKI67*	c.3254T>A	p.Met1085Lys	0.495	109	54	T	B	TPS

MCL27	*LRRK1*	c.261+6G>A		0.511	1894	967			TPS
MCL27	*TCF3*	c.1564A>C	p.Lys522Gln	0.547	519	284	T	B	TPS
MCL27	*CARD6*	c.372G>C	p.Gln124His	0.421	19	8	D	B	TPS
MCL27	*ACTB*	c.124-7T>A		0.390	336	131			TPS
MCL28*	*ATM*	c.6404dupT	p.Arg2136LysfsTer10	0.638	552	352			TPS
MCL28*	*NFKBIE*	c.759_762delTTAC	p.Tyr254SerfsTer13	0.401	968	388			TPS
MCL28*	*SYNE1*	c.7734-12_7734-11delCC		0.275	40	11			TPS
MCL28*	*LRRK1*	c.763G>A	p.Ala255Thr	0.523	511	267	T	B	TPS
MCL28*	*CARD11*	c.1202A>T	p.Asp401Val	0.405	437	177	D	D	TPS
MCL28*	*PKHD1L1*	c.6329C>T	p.Thr2110Ile	0.468	410	192	D	P	TPS
MCL28*	*NOTCH2*	c.710G>A	p.Arg237Gln	0.506	245	124	T	P	TPS
MCL28*	*MKI67*	c.9544C>G	p.Leu3182Val	0.491	285	140	T	P	TPS
MCL28*	*KLHL14*	c.1099G>A	p.Val367Ile	0.451	566	255	T	D	TPS
MCL28*	*CRYBG3*	c.899C>A	p.Pro300Gln	0.469	565	265		B	TPS
MCL28*	*TNFAIP3*	c.1274C>T	p.Pro425Leu	0.323	1152	372	T	B	TPS
MCL28*	*NOTCH1*	c.4046C>T	p.Ala1349Val	0.484	514	249	T	P	TPS
MCL29†	*SMARCA4*	c.3403C>T	p.Arg1135Trp	0.432	889	384	D	D	TPS
MCL29†	*ARID1A*	c.1903delA	p.Arg635AspfsTer12	0.360	275	99			TPS
MCL29†	*NFKBIE*	c.759_762delTTAC	p.Tyr254SerfsTer13	0.293	819	240			TPS
MCL29†	*CHEK2*	c.538C>T	p.Arg180Cys	0.437	199	87	D	P	TPS
MCL29†	*CHEK2*	c.886G>T	p.Asp296Tyr	0.657	35	23	D	D	TPS
MCL29†	*CDH23*	c.8401T>G	p.Phe2801Val	0.538	1014	546	T	B	TPS
MCL29†	*ATM*	c.7280T>G	p.Leu2427Arg	0.740	96	71	D	D	TPS
MCL29†	*MKI67*	c.3254T>A	p.Met1085Lys	0.471	399	188	T	B	TPS

MCL29†	*LRRK1*	c.261+6G>A		0.306	3688	1127			TPS
MCL29†	*GRIN2A*	c.418C>G	p.Pro140Ala	0.461	258	119	T	B	TPS
MCL29†	*TCF3*	c.1564A>C	p.Lys522Gln	0.515	482	248	T	B	TPS
MCL29†	*CARD6*	c.372G>C	p.Gln124His	0.489	90	44	D	B	TPS
MCL29†	*ACTB*	c.124-7T>A		0.488	689	336			TPS
MCL29†	*ESX1*	c.1013_1039dupGTGTGCCACCCGGGCCGCCCATGGCGC	p.Arg338_Ala346dup	0.653	98	64			TPS
MCL29†	*PCLO*	c.1124_1125delinsCTCTTGGTCCTGCTACCTCCCTCCAC	p.Gln375delinsProLeuGlyProAlaLysProProAlaGlnHis	0.181	177	32			WES
MCL29†	*ABCC9*	c.1657G>T	p.Ala553Ser	0.103	39	4	D	B	WES
MCL29†	*ANK2*	c.7267G>A	p.Ala2423Thr	0.769	26	20	D	B	WES
MCL29†	*ARID1A*	c.1903del	p.Arg635AspfsTer12	0.250	20	5			WES
MCL29†	*ATM*	c.7280T>G	p.Leu2427Arg	0.929	14	13	D	D	WES
MCL29†	*ATM*	c.3349C>A	p.Gln1117Lys	0.108	37	4	T	B	WES
MCL29†	*ATRX*	c.6236G>A	p.Arg2079Gln	0.114	35	4	D	D	WES
MCL29†	*CDH23*	c.1681T>G	p.Phe561Val	0.521	703	366	T	B	WES
MCL29†	*CHEK2*	c.667C>T	p.Arg223Cys	0.476	42	20	D	P	WES
MCL29†	*DLC1*	c.762_763delinsCG	p.GlnAsn254HisAsp	0.971	35	34			WES
MCL29†	*E2F1*	c.1171_1174delinsCC	p.Phe391ProfsTer6	0.430	286	123			WES
MCL29†	*FAT1*	c.6276_6277ins	p.Val2092_Asp2093ins	0.375	32	12			WES
MCL29†	*GRIN2A*	c.418C>G	p.Pro140Ala	0.707	41	29	T	B	WES
MCL29†	*MKI67*	c.4547_4550delinsAGCT	p.ArgPro1516GlnLeu	0.961	51	49			WES
MCL29†	*MKI67*	c.2174T>A	p.Met725Lys	0.427	82	35	T	B	WES
MCL29†	*MPDZ*	c.5242C>A	p.Arg1748Ser	0.342	41	14	D	D	WES

MCL29†	*NFKBIE*	c.759_762del	p.Tyr254SerfsTer13	0.403	300	121			WES
MCL29†	*NOTCH3*	c.4552C>A	p.Leu1518Met	0.497	330	164	D	D	WES
MCL29†	*PCDHB2*	c.2279_2280delinsAA	p.Gly760Glu	0.324	34	11			WES
MCL29†	*PCDHB2*	c.2021_2022delinsCT	p.Leu674Pro	0.266	218	58			WES
MCL29†	*PCLO*	c.7220C>T	p.Pro2407Leu	0.455	11	5	T	B	WES
MCL29†	*PKD1*	c.2102C>A	p.Thr701Asn	0.250	20	5	T	P	WES
MCL29†	*RELN*	c.7370G>A	p.Arg2457His	0.143	28	4	D	D	WES
MCL29†	*ROBO2*	c.73_75delinsATC	p.Val25Ile	0.163	92	15			WES
MCL29†	*RYR1*	c.10966A>G	p.Ser3656Gly	0.479	242	116	T	B	WES
MCL29†	*SMARCA4*	c.3403C>T	p.Arg1135Trp	0.354	311	110	D	D	WES
MCL29†	*TCF3*	c.1291_1293delinsAGT	p.Gly431Ser	0.946	186	176			WES
MCL29†	*TCF3*	c.1564A>C	p.Lys522Gln	0.472	180	85	T	B	WES
MCL29†	*ZNF117*	c.1247T>C	p.Leu416Pro	0.113	62	7	T	B	WES
MCL30†	*SP140*	c.2112T>A	p.Cys704Ter	0.431	332	143			TPS
MCL30†	*EWSR1*	c.1834C>T	p.Arg612Ter	0.684	500	342			TPS
MCL30†	*NFKBIE*	c.139delC	p.Arg47AlafsTer2	0.468	509	238			TPS
MCL30†	*COL11A2*	c.539G>A	p.Arg180His	0.399	158	63	T	B	TPS
MCL30†	*BLM*	c.1044G>A	p.Met348Ile	0.314	51	16	D	B	TPS
MCL30†	*FAT4*	c.13285G>T	p.Asp4429Tyr	0.413	560	231	D	P	TPS
MCL30†	*DIS3*	c.1462G>T	p.Asp488Tyr	0.429	28	12	D	D	TPS
MCL30†	*MAP3K14*	c.1868C>T	p.Pro623Leu	0.477	870	415			TPS
MCL30†	*U2AF2*	c.691G>A	p.Asp231Asn	0.382	144	55	D	D	TPS
MCL30†	*ATP6V1B2*	c.705+3A>G		0.174	23	4			TPS
MCL30†	*TYK2*	c.2098C>T	p.Arg700Trp	0.471	782	368	D	D	TPS

MCL30†	*PKHD1L1*	c.11590C>T	p.Arg3864Cys	0.333	36	12	D	D	TPS
MCL30†	*ARID1A*	c.6707G>A	p.Arg2236His	0.343	1068	366	D	D	TPS
MCL30†	*CDH23*	c.3352G>A	p.Gly1118Ser	0.479	495	237		B	TPS
MCL30†	*MMP8*	c.378G>C	p.Glu126Asp	0.529	34	18	D	B	TPS
MCL30†	*ETV6*	c.572G>T	p.Arg191Leu	0.472	527	249	D	B	TPS
MCL30†	*SRRM2*	c.5834G>A	p.Arg1945His	0.410	502	206		D	TPS
MCL30†	*ABCA6*	c.1830C>A	p.Asn610Lys	0.520	25	13	T	B	TPS
MCL30†	*TCF3*	c.1948C>A	p.Pro650Thr	0.499	1059	528	T	D	TPS
MCL30†	*MAP1B*	c.3166G>A	p.Gly1056Ser	0.484	281	136	T	B	TPS
MCL30†	*HEPH*	c.1490+6G>C		0.945	55	52			TPS
MCL31	*KMT2D*	c.11347C>T	p.Gln3783Ter	0.439	1232	541			TPS
MCL31	*ABCC9*	c.3669+10T>C		0.506	89	45			TPS
MCL31	*COL6A3*	c.92-6330C>T		0.483	2728	1318			TPS
MCL31	*DNAH5*	c.-8C>A		0.578	64	37			TPS
MCL31	*ABCA6*	c.1052T>C	p.Leu351Pro	0.518	83	43	D	D	TPS
MCL31	*ZNF521*	c.3906+5T>C		0.400	40	16			TPS
MCL31	*DLGAP2*	c.960G>C	p.Lys320Asn	0.502	1967	987	T	D	TPS
MCL31	*UBR5*	c.6631-11T>A		0.211	19	4			TPS
MCL31	*SPEN*	c.8399C>T	p.Ala2800Val	0.482	4602	2219	D	B	TPS
MCL31	*BCL10*	c.485C>T	p.Thr162Met	0.385	52	20	D	D	TPS
MCL31	*CDH23*	c.3352G>A	p.Gly1118Ser	0.479	1479	708		B	TPS
MCL31	*KMT2A*	c.218G>A	p.Gly73Glu	0.503	547	275	D	B	TPS
MCL31	*NIN*	c.1260-8C>T		0.383	47	18			TPS
MCL31	*MYBBP1A*	c.3203G>A	p.Arg1068His	0.501	1785	894	D	P	TPS

MCL31	*DNAH5*	c.4855A>G	p.Thr1619Ala	0.527	146	77	T	B	TPS
MCL31	*PLEKHG1*	c.2027G>A	p.Arg676Gln	0.423	352	149	D	D	TPS
MCL32†	*ATM*	c.1402_1403delAA	p.Lys468GlufsTer18	0.845	328	277			TPS
MCL32†	*NOTCH1*	c.7378G>T	p.Glu2460Ter	0.334	356	119			TPS
MCL32†	*NFKBIE*	c.139delC	p.Arg47AlafsTer2	0.414	309	128			TPS
MCL32†	*APC*	c.757G>A	p.Gly253Ser	0.483	971	469	T	B	TPS
MCL32†	*COL11A2*	c.4229A>C	p.Lys1410Thr	0.485	266	129	D	D	TPS
MCL32†	*PLXNB3*	c.2453G>A	p.Arg818His	0.436	353	154	D	P	TPS
MCL32†	*CDH23*	c.8394C>G	p.Ile2798Met	0.534	324	173	T	B	TPS
MCL32†	*MKI67*	c.655A>C	p.Lys219Gln	0.482	691	333	D	D	TPS
MCL32†	*ARID2*	c.2806G>T	p.Gly936Cys	0.489	364	178	D	D	TPS
MCL32†	*LRP1B*	c.2281A>G	p.Ile761Val	0.475	375	178	T	B	TPS
MCL32†	*EP300*	c.5897G>T	p.Gly1966Val	0.453	256	116	T	P	TPS
MCL32†	*ANK2*	c.7159G>C	p.Ala2387Pro	0.437	357	156	T	B	TPS
MCL32†	*ANK2*	c.11320G>A	p.Val3774Met	0.558	217	121	D	D	TPS
MCL32†	*CD14*	c.586C>T	p.Leu196Phe	0.458	719	329	D	D	TPS
MCL33*	*FBXW7*	c.1418+1G>A		0.176	170	30			TPS
MCL33*	*CCND3*	c.811dupC	p.Arg271ProfsTer53	0.188	256	48			TPS
MCL33*	*PRDM1*	c.291+1G>A		0.435	246	107			TPS
MCL33*	*ABCA7*	c.3148-5C>G		0.482	139	67			TPS
MCL33*	*IRF4*	c.-2G>T		0.332	340	113			TPS
MCL33*	*PIM1*	c.72G>C	p.Lys24Asn	0.260	296	77	T	B	TPS
MCL33*	*CARD11*	c.1159G>T	p.Asp387Tyr	0.750	96	72	D	D	TPS
MCL33*	*KMT2C*	c.8069A>T	p.Asp2690Val	0.267	161	43	D	D	TPS

MCL33*	*FIGN*	c.1531G>A	p.Ala511Thr	0.419	74	31	T	D	TPS
MCL33*	*IGLL5*	c.173G>A	p.Ser58Asn	0.457	267	122	D	B	TPS
MCL33*	*IGLL5*	c.182C>T	p.Ser61Phe	0.398	256	102	T	B	TPS
MCL33*	*SI*	c.4999G>A	p.Val1667Ile	0.350	117	41	T	B	TPS
MCL33*	*ANK2*	c.6073G>A	p.Gly2025Ser	0.422	161	68	T	B	TPS
MCL33*	*ANK2*	c.10019A>G	p.Asp3340Gly	0.471	157	74	T	B	TPS
MCL33*	*PDLIM3*	c.14T>C	p.Val5Ala	0.402	117	47	D	D	TPS
MCL33*	*MAP1B*	c.4198A>G	p.Ser1400Gly	0.459	135	62	D	P	TPS
MCL33*	*PCLO*	c.3539T>C	p.Leu1180Pro	0.444	99	44	T	B	TPS
MCL34	*SPEN*	c.3508C>T	p.Arg1170Ter	0.345	238	82			TPS
MCL34	*TRAF2*	c.637C>T	p.Arg213Ter	0.363	113	41			TPS
MCL34	*WDR66*	c.25C>T	p.Arg9Ter	0.353	156	55			TPS
MCL34	*TP53*	c.821T>C	p.Val274Ala	0.581	172	100	D	P	TPS
MCL34	*BCL9*	c.808C>A	p.Pro270Thr	0.453	106	48	T	B	TPS
MCL34	*BCL9*	c.1255G>C	p.Gly419Arg	0.378	82	31	D	P	TPS
MCL34	*CCND1*	c.139T>A	p.Cys47Ser	0.558	319	178	T	B	TPS
MCL34	*ARID1B*	c.1738-29732T>C		0.353	133	47	T	B	TPS
MCL34	*KMT2C*	c.11575G>A	p.Glu3859Lys	0.573	286	164	D	D	TPS
MCL34	*CTCF*	c.1519-8C>T		0.459	61	28			TPS
MCL35†	*MPEG1*	c.445C>T	p.Arg149Ter	0.246	69	17			TPS
MCL35†	*ABCC9*	c.2857G>A	p.Glu953Lys	0.366	41	15	T	P	TPS
MCL35†	*KMT2D*	c.7490C>T	p.Ala2497Val	0.197	61	12	D	P	TPS
MCL35†	*MEF2B*	c.68A>G	p.Lys23Arg	0.225	89	20	D	D	TPS
MCL35†	*MTOR*	c.5614-10G>C		0.298	84	25			TPS

MCL35†	*MMP8*	c.1177G>A	p.Asp393Asn	0.273	11	3	T	B	TPS
MCL35†	*DSC1*	c.770C>T	p.Ser257Phe	0.563	16	9	T	B	TPS
MCL35†	*ZNF117*	c.899G>C	p.Ser300Thr	0.130	23	3	T	B	TPS
MCL35†	*COL14A1*	c.3466G>A	p.Glu1156Lys	0.194	36	7	T	D	TPS
MCL35†	*HEATR1*	c.5355+10G>A		0.214	42	9			TPS
MCL35†	*ZFHX3*	c.10445G>T	p.Ser3482Ile	0.317	63	20	D	B	TPS
MCL35†	*MUC16*	c.10543C>T	p.His3515Tyr	0.527	55	29	D	B	TPS
MCL35†	*NSD1*	c.11C>A	p.Thr4Asn	0.365	63	23	D	B	TPS
MCL35†	*ABL1*	c.172C>T	p.Pro58Ser	0.281	64	18	D	B	TPS
MCL35†	*NOTCH1*	c.5870A>C	p.Gln1957Pro	0.384	99	38	D	B	TPS
MCL36†	*S1PR1*	c.10dupA	p.Thr4AsnfsTer15	0.269	104	28			TPS
MCL36†	*ATP11C*	c.3055C>T	p.Arg1019Ter	0.261	23	6			TPS
MCL36†	*COL4A2*	c.5068G>A	p.Ala1690Thr	0.327	101	33	D	P	TPS
MCL36†	*ZNF85*	c.1520C>G	p.Thr507Arg	0.339	56	19	D	D	TPS
MCL36†	*ARID1B*	c.787A>T	p.Met263Leu	0.409	269	110	D	B	TPS
MCL36†	*SLC29A2*	c.475T>G	p.Tyr159Asp	0.496	117	58	D	D	TPS
MCL36†	*NAV3*	c.3880G>A	p.Gly1294Ser	0.349	83	29	T	D	TPS
MCL36†	*WHSC1*	c.3295G>A	p.Glu1099Lys	0.208	96	20	D	D	TPS
MCL36†	*FAT4*	c.14929G>A	p.Ala4977Thr	0.410	39	16	T	B	TPS
MCL36†	*PALLD*	c.1354G>A	p.Val452Met	0.477	86	41	D	D	TPS
MCL36†	*ABCA13*	c.5888A>C	p.Asn1963Thr	0.455	55	25	D	P	TPS
MCL36†	*ABCA13*	c.10327G>A	p.Asp3443Asn	0.679	56	38	T	D	TPS
MCL36†	*PKHD1L1*	c.3848A>G	p.Gln1283Arg	0.478	46	22	T	B	TPS
MCL36†	*SCRIB*	c.641C>T	p.Pro214Leu	0.159	88	14	D	D	TPS

MCL37†	*ARID1B*	c.1650dupT	p.Gly551TrpfsTer102	0.254	401	102			TPS
MCL37†	*NOTCH1*	c.6849_6850insGACG	p.Thr2284AspfsTer71	0.283	237	67			TPS
MCL37†	*TP53*	c.91G>A	p.Val31Ile	0.444	54	24	T	B	TPS
MCL37†	*CCND1*	c.142G>A	p.Val48Met	0.376	636	239	D	D	TPS
MCL37†	*CCND1*	c.149A>G	p.Lys50Arg	0.379	625	237	T	B	TPS
MCL37†	*SETD2*	c.4715+9T>C		0.450	222	100			TPS
MCL37†	*KCNC2*	c.512C>A	p.Thr171Asn	0.427	583	249	T	B	TPS
MCL37†	*PHLPP1*	c.1864A>G	p.Thr622Ala	0.459	185	85	T	D	TPS
MCL37†	*DLC1*	c.1349-3T>C		0.460	139	64			TPS
MCL37†	*COL14A1*	c.4637G>A	p.Arg1546His	0.228	114	26	T	D	TPS
MCL37†	*KDM4C*	c.1304C>G	p.Ala435Gly	0.459	364	167	T	B	TPS
MCL37†	*PLXNB3*	c.4861G>A	p.Val1621Ile	0.459	194	89	T	B	TPS
MCL37†	*ARID1A*	c.3999_4001dup	p.Gln1334dup	0.438	290	127			WES
MCL37†	*ARID1B*	c.1650dup	p.Gly551TrpfsTer89	0.261	153	40			WES
MCL37†	*CARD11*	c.383C>T	p.Thr128Met	0.182	77	14	D	D	WES
MCL37†	*CCND1*	c.142G>A	p.Val48Met	0.292	178	52	D	D	WES
MCL37†	*CCND1*	c.149A>G	p.Lys50Arg	0.272	180	49	T	B	WES
MCL37†	*COL14A1*	c.4637G>A	p.Arg1546His	0.186	140	26	T	D	WES
MCL37†	*COL14A1*	c.2771A>G	p.Asn924Ser	0.464	239	111	D	B	WES
MCL37†	*DLC1*	c.762_763delinsCG	p.GlnAsn254HisAsp	0.942	104	98			WES
MCL37†	*KCNC2*	c.512C>A	p.Thr171Asn	0.432	37	16	T	B	WES
MCL37†	*KDM4C*	c.1304C>G	p.Ala435Gly	0.506	81	41	T	B	WES
MCL37†	*KMT2D*	c.13885A>C	p.Thr4629Pro	0.208	24	5	D	D	WES
MCL37†	*NOTCH1*	c.6849_6850ins	p.Thr2284AspfsTer71	0.295	95	28			WES

MCL37†	*ROBO2*	c.73_75delinsATC	p.Val25Ile	0.129	286	37			WES
MCL37†	*SYNE1*	c.8578A>G	p.Thr2860Ala	0.648	88	57	D	B	WES
MCL37†	*SYNE1*	c.12149_12150delinsGT	p.Lys4050Ser	0.989	92	91			WES
MCL38	*CHEK2*	c.1111C>T	p.His371Tyr	0.405	121	49	D	P	TPS
MCL38	*TNFRSF13B*	c.17G>A	p.Arg6Gln	0.431	218	94	D	B	TPS
MCL38	*FBXO21*	c.202A>T	p.Ser68Cys	0.152	297	45	D	P	TPS
MCL38	*DCAF4L2*	c.139C>T	p.Arg47Cys	0.179	196	35	D	D	TPS
MCL38	*FGFR1*	c.2201G>A	p.Arg734Gln	0.491	216	106	D	D	TPS
MCL38	*PRDM2*	c.3190T>A	p.Phe1064Ile	0.529	34	18	T	B	TPS
MCL38	*DNAJC6*	c.1367A>G	p.Asn456Ser	0.383	209	80	T	B	TPS
MCL38	*NCOR2*	c.6532A>G	p.Ser2178Gly	0.436	179	78	T	B	TPS
MCL38	*WHSC1*	c.3295G>A	p.Glu1099Lys	0.162	291	47	D	D	TPS
MCL38	*APC*	c.1145G>A	p.Arg382Lys	0.498	217	108	T	B	TPS
MCL38	*ARID1B*	c.993_1007delAGGAGGAGCAGGAGC	p.Gly333_Gly337del	0.244	90	22			TPS
MCL38	*ACTB*	c.124-7T>A		0.464	151	70			TPS
MCL39	*ACTN2*	c.1040C>T	p.Thr347Met	0.539	345	186	D	D	TPS
MCL39	*CSF1R*	c.1606C>G	p.Leu536Val	0.455	165	75	T	D	TPS
MCL39	*ESX1*	c.1013_1039dupGTGTGCCACCCGGGCCGCCCATGGCGC	p.Arg338_Ala346dup	0.725	51	37			TPS
MCL39	*LRP1B*	c.7179T>G	p.Asp2393Glu	0.530	236	125	D	D	TPS
MCL39	*CRYBG3*	c.116G>A	p.Ser39Asn	0.461	456	210		P	TPS
MCL39	*NOTCH1*	c.6652C>G	p.Leu2218Val	0.481	707	340	T	P	TPS
MCL39	*PLXNB3*	c.4582G>A	p.Gly1528Arg	0.521	817	426	T	B	TPS
MCL39	*LRRK1*	c.439T>C	p.Cys147Arg	0.488	293	143	T	B	TPS

MCL39	*TCF3*	c.148C>T	p.Leu50Phe	0.493	217	107	D	D	TPS
MCL39	*MUC16*	c.10543C>T	p.His3515Tyr	0.564	195	110	D	B	TPS
MCL39	*EP300*	c.1283-6C>G		0.520	100	52			TPS
MCL39	*WHSC1*	c.3295G>A	p.Glu1099Lys	0.263	308	81	D	D	TPS
MCL39	*CD83*	c.220C>T	p.Arg74Cys	0.529	272	144	D	B	TPS
MCL39	*CARD11*	c.2081C>T	p.Ser694Leu	0.580	572	332	T	B	TPS
MCL39	*ABCA13*	c.3821A>G	p.Glu1274Gly	0.531	177	94	D	B	TPS
MCL39	*MYC*	c.1163G>A	p.Ser388Asn	0.252	361	91	D	D	TPS
MCL40	*WDR90*	c.4057C>T	p.Arg1353Ter	0.267	15	4			TPS
MCL40	*KMT2D*	c.6007C>A	p.Leu2003Ile	0.273	22	6	D	D	TPS
MCL40	*SF1*	c.1501A>G	p.Met501Val	0.417	12	5	T	B	TPS
MCL40	*NCOR2*	c.4073-6C>T		0.385	13	5			TPS
MCL40	*TBC1D26*	c.167A>C	p.Glu56Ala	0.182	11	2	D	P	TPS
MCL40	*PCSK2*	c.1879G>T	p.Val627Leu	0.724	29	21	T	P	TPS
MCL40	*ABCA13*	c.10327G>A	p.Asp3443Asn	0.533	15	8	T	D	TPS
MCL41†	*KMT2D*	c.8727_8730delAAGT	p.Ser2910ArgfsTer32	0.324	37	12			TPS
MCL41†	*ATM*	c.1802+2T>C		0.188	32	6			TPS
MCL41†	*PASK*	c.1811dupG	p.Ser605GlnfsTer12	0.500	34	17			TPS
MCL41†	*RP1L1*	c.235C>T	p.Arg79Cys	0.322	118	38	D	D	TPS
MCL41†	*DNAH5*	c.6956C>T	p.Thr2319Met	0.423	26	11	D	D	TPS
MCL41†	*PRDM2*	c.4826G>A	p.Arg1609Gln	0.232	56	13	D	D	TPS
MCL41†	*IKBKB*	c.-18-10T>A		0.381	21	8			TPS
MCL41†	*KMT2A*	c.7070C>G	p.Pro2357Arg	0.542	48	26	D	B	TPS
MCL41†	*EIF2AK4*	c.3529A>G	p.Ile1177Val	0.484	128	62	T	B	TPS

MCL41†	*FLYWCH1*	c.962G>A	p.Arg321Gln	0.371	202	75	T	D	TPS
MCL41†	*CDH8*	c.1247G>A	p.Arg416His	0.436	39	17	T	B	TPS
MCL41†	*CDH11*	c.932A>G	p.Asp311Gly	0.565	124	70	T	D	TPS
MCL41†	*KLHL14*	c.1841C>T	p.Ala614Val	0.523	44	23	D	P	TPS
MCL41†	*ATR*	c.3637A>G	p.Ser1213Gly	0.525	61	32	D	D	TPS
MCL41†	*BCL6*	c.1550C>T	p.Ala517Val	0.338	80	27	T	B	TPS
MCL41†	*BCL6*	c.1549G>A	p.Ala517Thr	0.316	76	24	T	B	TPS
MCL41†	*PKHD1L1*	c.10813C>T	p.Leu3605Phe	0.647	51	33	T	P	TPS
MCL41†	*ATM*	c.7310_7321dup	p.Lys2440_Val2441ins	0.360	25	9			WES
MCL41†	*COL11A1*	c.3727G>T	p.Gly1243Trp	0.114	35	4	D	D	WES
MCL41†	*KMT2D*	c.8727_8730del	p.Ser2910ArgfsTer32	0.422	121	51			WES
MCL41†	*PRDM2*	c.4826G>A	p.Arg1609Gln	0.500	14	7	D	D	WES
MCL41†	*ROBO2*	c.2066C>A	p.Ala689Asp	0.129	31	4	D	B	WES
MCL42	*KMT2D*	c.15597delT	p.His5200IlefsTer43	0.293	1871	549			TPS
MCL42	*GPR37*	c.1564delG	p.Val522PhefsTer57	0.284	841	239			TPS
MCL42	*BRCA2*	c.4854T>A	p.Asp1618Glu	0.657	527	346	T	B	TPS
MCL42	*ABCA7*	c.946G>A	p.Glu316Lys	0.478	694	332	D	P	TPS
MCL42	*ANK2*	c.8015A>C	p.Gln2672Pro	0.514	729	375	D	P	TPS
MCL42	*ATM*	c.6096A>C	p.Arg2032Ser	0.474	504	239	D	D	TPS
MCL42	*BIRC3*	c.1802C>A	p.Thr601Lys	0.283	336	95	D	D	TPS
MCL42	*SYNE1*	c.1710G>C	p.Leu570Phe	0.369	739	273	D	D	TPS
MCL42	*SLC29A2*	c.112-9A>G		0.487	973	474			TPS
MCL42	*DTX1*	c.1318C>T	p.Arg440Cys	0.642	1980	1272	T	B	TPS
MCL42	*CDH8*	c.1686T>G	p.Asn562Lys	0.518	500	259	T	B	TPS

MCL42	*LRP1B*	c.2396G>A	p.Arg799Gln	0.512	736	377	T	P	TPS
MCL42	*ATP10B*	c.1388G>A	p.Arg463Gln	0.300	787	236	D	D	TPS
MCL42	*PLEKHG1*	c.289G>A	p.Gly97Arg	0.606	1346	816	T	B	TPS
MCL43	*CDH23*	c.5747G>A	p.Arg1916His	0.410	1233	505		D	TPS
MCL43	*ASXL1*	c.1465C>G	p.Arg489Gly	0.450	1115	502	D	P	TPS
MCL43	*SYNE1*	c.16688T>C	p.Met5563Thr	0.490	290	142	D	D	TPS
MCL43	*SPEN*	c.9032G>A	p.Arg3011Gln	0.465	1413	657	D	D	TPS
MCL43	*FLG*	c.8888C>T	p.Ala2963Val	0.167	30	5	D	B	TPS
MCL43	*ARID5B*	c.333G>C	p.Lys111Asn	0.501	533	267	D	D	TPS
MCL44	*ATM*	c.1333C>T	p.Gln445Ter	0.191	618	118			TPS
MCL44	*ULK4*	c.1797G>A	p.Trp599Ter	0.430	632	272			TPS
MCL44	*ATM*	c.7355T>C	p.Leu2452Pro	0.166	566	94	D	D	TPS
MCL44	*ABCA7*	c.1570C>T	p.Arg524Trp	0.525	2262	1187	D	D	TPS
MCL44	*CCND1*	c.106G>C	p.Glu36Gln	0.282	3919	1104	D	P	TPS
MCL44	*KIAA1671*	c.5343-9T>C		0.512	592	303			TPS
MCL44	*DNAH5*	c.6589G>A	p.Asp2197Asn	0.463	309	143	D	D	TPS
MCL44	*RIMS2*	c.1676C>T	p.Ala559Val	0.575	193	111	T	P	TPS
MCL44	*DNAJC6*	c.1367A>G	p.Asn456Ser	0.490	1338	656	T	B	TPS
MCL44	*ARID1B*	c.5405G>A	p.Arg1802Gln	0.550	1377	758	T	P	TPS
MCL45	*PRF1*	c.1090_1091delCT	p.Leu364GlufsTer93	0.470	736	346			TPS
MCL45	*ABCA3*	c.873+10C>T		0.439	526	231			TPS
MCL45	*LAMA2*	c.2288C>T	p.Ala763Val	0.461	1326	611	D	D	TPS
MCL45	*ATP10B*	c.1459G>C	p.Ala487Pro	0.489	1037	507	T	B	TPS
MCL45	*EP300*	c.1283-6C>G		0.489	186	91			TPS

MCL45	*CARD6*	c.2012C>T	p.Ser671Phe	0.480	893	429	D	P	TPS
MCL45	*ARID1B*	c.6017C>T	p.Ala2006Val	0.456	1204	549	T	B	TPS
MCL45	*HEPH*	c.2309G>C	p.Ser770Thr	0.996	505	503	T	B	TPS
MCL46	*SYNE1*	c.17654G>A	p.Arg5885His	0.430	374	161	D	D	TPS
MCL46	*COL11A1*	c.1669G>A	p.Gly557Arg	0.626	254	159	T	P	TPS
MCL46	*CDH23*	c.8062A>G	p.Ser2688Gly	0.527	552	291	T	B	TPS
MCL46	*TCF12*	c.484A>G	p.Thr162Ala	0.432	398	172	T	P	TPS
MCL46	*FAT1*	c.755A>C	p.Glu252Ala	0.512	642	329	T	B	TPS
MCL46	*CARD11*	c.2081C>T	p.Ser694Leu	0.475	1833	870	T	B	TPS
MCL46	*ABL1*	c.1678G>A	p.Asp560Asn	0.404	552	223	T	B	TPS
MCL47	*DNAH5*	c.6884C>T	p.Ala2295Val	0.496	284	141	D	D	TPS
MCL47	*PTPN11*	c.148G>A	p.Ala50Thr	0.460	139	64	T	B	TPS
MCL47	*CDC123*	c.717+7A>G		0.435	138	60			TPS
MCL47	*CASP10*	c.1045G>T	p.Asp349Tyr	0.473	419	198	D	D	TPS
MCL47	*COL6A3*	c.5419G>A	p.Glu1807Lys	0.441	805	355	D	D	TPS
MCL47	*SPEN*	c.8161G>A	p.Ala2721Thr	0.492	695	342	T	B	TPS
MCL47	*CSF3R*	c.1096G>A	p.Gly366Arg	0.418	538	225	D	D	TPS
MCL47	*MMP8*	c.379G>A	p.Ala127Thr	0.448	310	139	T	B	TPS
MCL47	*TBC1D26*	c.547-4G>A		0.483	300	145			TPS
MCL47	*TCF3*	c.1592C>T	p.Thr531Met	0.435	301	131		D	TPS
MCL47	*ANKRD44*	c.1458-10T>C		0.459	207	95			TPS
MCL47	*PDGFRB*	c.11C>T	p.Pro4Leu	0.492	311	153	T	B	TPS
MCL47	*ABCA13*	c.10327G>A	p.Asp3443Asn	0.464	362	168	T	D	TPS
MCL47	*COL14A1*	c.1195G>A	p.Val399Met	0.444	205	91	D	D	TPS

MCL48*	*KMT2D*	c.4877dupT	p.Leu1626PhefsTer5	0.670	887	594			TPS
MCL48*	*NAV3*	c.694C>T	p.Gln232Ter	0.407	639	260			TPS
MCL48*	*NLRC5*	c.5062C>T	p.Gln1688Ter	0.400	80	32			TPS
MCL48*	*SI*	c.4693-2A>T		0.387	419	162			TPS
MCL48*	*CCND3*	c.831_832insT	p.Pro278SerfsTer46	0.578	656	379			TPS
MCL48*	*TNFAIP3*	c.771dupT	p.Val258CysfsTer12	0.578	941	544			TPS
MCL48*	*DOCK4*	c.2770C>T	p.Arg924Ter	0.310	287	89			TPS
MCL48*	*FLG*	c.3321delA	p.Gly1109GlufsTer13	0.885	200	177			TPS
MCL48*	*KMT2D*	c.6640G>A	p.Ala2214Thr	0.276	417	115	T	B	TPS
MCL48*	*IKZF1*	c.59G>T	p.Ter20TyrextTer?	0.367	1160	426			TPS
MCL48*	*CREBBP*	c.1319G>A	p.Arg440Gln	0.365	572	209	D	D	TPS
MCL48*	*MEF2B*	c.163T>C	p.Phe55Leu	0.413	579	239	D	D	TPS
MCL48*	*SMC3*	c.998T>G	p.Leu333Arg	0.379	501	190	D	D	TPS
MCL48*	*CHEK1*	c.1417C>T	p.Leu473Phe	0.330	561	185	T	B	TPS
MCL48*	*BCL7A*	c.70G>C	p.Ala24Pro	0.345	1131	390	D	P	TPS
MCL48*	*BCL7A*	c.82G>A	p.Val28Met	0.346	1011	350	D	D	TPS
MCL48*	*CD70*	c.175G>A	p.Glu59Lys	0.397	131	52	D	D	TPS
MCL48*	*IGLL5*	c.154C>T	p.Pro52Ser	0.204	294	60	D	B	TPS
MCL48*	*IGLL5*	c.206+8G>C		0.272	158	43			TPS
MCL48*	*FAT4*	c.1774G>T	p.Val592Leu	0.315	391	123	T	B	TPS
MCL48*	*HIST1H1C*	c.516G>C	p.Lys172Asn	0.606	792	480	D	D	TPS
MCL48*	*ARID1B*	c.347A>G	p.Gln116Arg	0.347	1087	377	D	B	TPS
MCL48*	*PCLO*	c.5111T>A	p.Leu1704Gln	0.395	157	62	D	D	TPS
MCL48*	*ESX1*	c.1015G>C	p.Val339Leu	0.540	359	194	T	B	TPS

MCL48*	*NOTCH2*	c.1454-6G>T		0.414	374	155			TPS
MCL48*	*CDH23*	c.1990G>A	p.Glu664Lys	0.556	135	75		P	TPS
MCL48*	*ZNF521*	c.1546C>T	p.Pro516Ser	0.592	382	226	T	P	TPS
MCL48*	*LRP1B*	c.12956G>A	p.Arg4319Lys	0.363	182	66	T	B	TPS
MCL48*	*IGLL5*	c.140G>C	p.Ser47Thr	0.512	344	176	D	P	TPS
MCL48*	*GNAT1*	c.508G>A	p.Val170Met	0.235	519	122	D	D	TPS
MCL48*	*FBXW7*	c.1236+6T>A		0.466	491	229			TPS
MCL48*	*ESX1*	c.1013G>C	p.Arg338Pro	0.519	349	181	T	B	TPS
MCL49†	*PKD1*	c.9136C>T	p.Arg3046Cys	0.293	242	71	D	D	TPS
MCL49†	*SYNE1*	c.8726C>T	p.Ser2909Leu	0.235	153	36	T	B	TPS
MCL49†	*TRPM6*	c.4591C>T	p.Arg1531Cys	0.326	95	31	T	B	TPS
MCL49†	*PDCD1LG2*	c.131T>C	p.Phe44Ser	0.200	110	22	D	D	TPS
MCL49†	*MTOR*	c.3401C>T	p.Ala1134Val	0.321	84	27	T	B	TPS
MCL49†	*BCL10*	c.485C>T	p.Thr162Met	0.525	80	42	D	D	TPS
MCL49†	*ZNF296*	c.1393C>T	p.Arg465Trp	0.370	284	105	D	P	TPS
MCL49†	*CREG2*	c.502A>G	p.Thr168Ala	0.258	93	24	D	D	TPS
MCL49†	*TNFAIP3*	c.1397G>T	p.Ser466Ile	0.167	198	33	D	D	TPS
MCL50*	*ULK4*	c.1797G>A	p.Trp599Ter	0.406	96	39			TPS
MCL50*	*RB1*	c.1861C>A	p.Arg621Ser	0.536	56	30	T	B	TPS
MCL50*	*BRIP1*	c.2854A>G	p.Ile952Val	0.420	100	42	T	B	TPS
MCL50*	*ATR*	c.190A>G	p.Thr64Ala	0.600	35	21	T	B	TPS
MCL50*	*SYNE1*	c.13511G>T	p.Cys4504Phe	0.575	73	42	D	D	TPS
MCL50*	*CCND1*	c.139T>C	p.Cys47Arg	0.484	2220	1074	D	B	TPS
MCL50*	*TCTN2*	c.1724C>T	p.Ala575Val	0.409	477	195	D	P	TPS

MCL50*	*SLITRK6*	c.302C>T	p.Ala101Val	0.455	88	40	T	B	TPS
MCL50*	*U2AF2*	c.691G>A	p.Asp231Asn	0.414	512	212	D	D	TPS
MCL50*	*ATR*	c.1481C>G	p.Ala494Gly	0.494	85	42	T	B	TPS
MCL50*	*ZNF117*	c.899G>C	p.Ser300Thr	0.194	36	7	T	B	TPS
MCL50*	*PRDM2*	c.5060G>A	p.Arg1687Gln	0.468	1064	498	D	D	TPS
MCL50*	*HEATR1*	c.5355+10G>A		0.402	876	352			TPS
MCL50*	*ZEB1*	c.242A>G	p.Asn81Ser	0.500	108	54	T	B	TPS
MCL50*	*MKI67*	c.4589G>A	p.Arg1530Gln	0.401	521	209	T	P	TPS
MCL50*	*IRF8*	c.19G>A	p.Gly7Ser	0.354	390	138	T	D	TPS
MCL50*	*MAP3K14*	c.2281G>A	p.Gly761Arg	0.441	1007	444		D	TPS
MCL50*	*ABCA7*	c.4590G>A	p.Met1530Ile	0.458	629	288	T	B	TPS
MCL50*	*BCR*	c.1402A>G	p.Ser468Gly	0.518	1522	788	D	B	TPS
MCL50*	*ANK2*	c.2900+5165A>G		0.465	71	33	D	D	TPS
MCL50*	*MAP1B*	c.2768T>C	p.Ile923Thr	0.489	319	156	T	B	TPS
MCL50*	*CCND1*	c.139T>C	p.Cys47Arg	0.416	202	84	D	B	WES
MCL50*	*KMT2D*	c.1940dup	p.Pro648ThrfsTer2	0.154	52	8			WES
MCL50*	*MEF2B*	c.68A>G	p.Lys23Arg	0.438	240	105	D	D	WES
MCL50*	*U2AF2*	c.691G>A	p.Asp231Asn	0.427	241	103	D	D	WES
MCL51	*PTEN*	c.521_522delAT	p.Tyr174CysfsTer5	0.551	207	114			TPS
MCL51	*ATRX*	c.2806G>C	p.Val936Leu	0.496	234	116	T	B	TPS
MCL51	*ACTN2*	c.1040C>T	p.Thr347Met	0.479	1015	486	D	D	TPS
MCL51	*CCND1*	c.131A>G	p.Tyr44Cys	0.608	2120	1290	D	D	TPS
MCL51	*ATM*	c.449T>C	p.Leu150Pro	0.786	304	239	D	D	TPS
MCL51	*PKHD1L1*	c.11590C>T	p.Arg3864Cys	0.504	365	184	D	D	TPS

MCL51	*MKI67*	c.1927T>C	p.Cys643Arg	0.315	336	106	D	D	TPS
MCL51	*TCF3*	c.1592C>T	p.Thr531Met	0.380	353	134		D	TPS
MCL51	*ZNF85*	c.32T>C	p.Ile11Thr	0.482	361	174	D	D	TPS
MCL51	*TNRC6B*	c.871G>A	p.Asp291Asn	0.483	588	284	T	P	TPS
MCL51	*ATR*	c.265A>G	p.Ser89Gly	0.588	434	255	D	B	TPS
MCL51	*ZNF608*	c.1388G>T	p.Gly463Val	0.696	230	160	D	D	TPS
MCL51	*ATP10B*	c.2246G>A	p.Arg749His	0.453	909	412	T	D	TPS
MCL51	*SYNE1*	c.20293C>T	p.Arg6765Cys	0.322	304	98	D	D	TPS
MCL51	*ACTB*	c.985-9C>T		0.292	620	181			TPS
MCL52	*TP53*	c.711G>T	p.Met237Ile	0.305	932	284	D	D	TPS
MCL52	*SP140*	c.1365dupC	p.Cys456LeufsTer20	0.352	1096	386			TPS
MCL52	*TP53*	XR_243566.1:n.850G>T		0.305	932	284	D	D	TPS
MCL52	*ATM*	c.7358G>C	p.Arg2453Pro	0.560	405	227	T	P	TPS
MCL52	*TET2*	c.2440C>T	p.Arg814Cys	0.500	806	403	D	B	TPS
MCL52	*BIRC3*	c.244C>T	p.Pro82Ser	0.392	477	187	D	D	TPS
MCL52	*SMARCA4*	c.3547G>A	p.Asp1183Asn	0.356	775	276	D	D	TPS
MCL52	*ACTN4*	c.913-7C>G		0.433	409	177			TPS
MCL52	*DNAH5*	c.10658G>A	p.Arg3553Gln	0.420	509	214	D	P	TPS
MCL52	*SF1*	c.855-5T>C		0.470	251	118			TPS
MCL52	*NRXN3*	c.1085G>A	p.Arg362His	0.260	538	140	D	D	TPS
MCL52	*ANKRD44*	c.2144A>G	p.His715Arg	0.495	562	278	T	D	TPS
MCL52	*TNRC6B*	c.1966G>A	p.Ala656Thr	0.499	805	402	D	B	TPS
MCL52	*DLC1*	c.1042G>C	p.Asp348His	0.347	449	156	D	D	TPS
MCL53	*ZFHX3*	c.9583_9584insT	p.Pro3195LeufsTer44	0.532	523	278			TPS

MCL53	*ABCA3*	c.1669T>C	p.Tyr557His	0.472	644	304	D	D	TPS
MCL53	*COL11A1*	c.3853-10C>A		0.222	90	20			TPS
MCL53	*ANK2*	c.10682-8T>C		0.462	238	110			TPS
MCL53	*CARD6*	c.2326G>A	p.Ala776Thr	0.419	227	95	T	B	TPS
MCL53	*PCLO*	c.12217C>G	p.Leu4073Val	0.563	334	188	T	B	TPS
MCL53	*PKD1*	c.6928G>A	p.Gly2310Arg	0.480	371	178	T	D	TPS
MCL53	*MKI67*	c.2720G>A	p.Arg907Lys	0.386	254	98	T	B	TPS
MCL53	*CDH8*	c.2084T>C	p.Leu695Ser	0.573	302	173	T	B	TPS
MCL53	*IRF8*	c.19G>A	p.Gly7Ser	0.416	807	336	T	D	TPS
MCL53	*ABCA6*	c.647A>C	p.Asn216Thr	0.480	321	154	T	B	TPS
MCL53	*BCL2L11*	c.*30T>C		0.388	245	95			TPS
MCL53	*PRDM1*	c.89C>G	p.Ala30Gly	0.494	640	316	T	P	TPS
MCL53	*KDM4C*	c.1304C>G	p.Ala435Gly	0.501	495	248	T	B	TPS

Supplementary Table S7. Genetic variants identified in 53 patients with mantle cell lymphoma. VAF, variant allele frequency; D, damaging; T, tolerated; P, possibly damaging; B, benign; TPS, targeted panel sequencing; WES, whole-exome sequencing. [*]Initial sample. [†]Relapse sample.
